# Alternative low-populated conformations prompt phase transitions in polyalanine repeat expansions

**DOI:** 10.1038/s41467-024-46236-5

**Published:** 2024-03-02

**Authors:** Rosa Antón, Miguel Á. Treviño, David Pantoja-Uceda, Sara Félix, María Babu, Eurico J. Cabrita, Markus Zweckstetter, Philip Tinnefeld, Andrés M. Vera, Javier Oroz

**Affiliations:** 1 Instituto de Química Física Blas Cabrera (IQF), CSIC, E-28006 Madrid, Spain; 2 Associate Laboratory i4HB – Institute for Health and Bioeconomy, NOVA School of Science and Technology, Universidade NOVA de Lisboa, 2819-516 Caparica, Portugal; 3 UCIBIO, Department of Chemistry, NOVA School of Science and Technology, Universidade NOVA de Lisboa, 2819-516 Caparica, Portugal; 4 German Center for Neurodegenerative Diseases (DZNE), 37075 Göttingen, Germany; 5 Department for NMR-based Structural Biology, Max Planck Institute for Multidisciplinary Sciences, 37077 Göttingen, Germany; 6 Department of Chemistry and Center for NanoScience, Ludwig-Maximilians-Universität München, München, 81377 Germany

**Keywords:** Solution-state NMR, Supramolecular assembly, Protein aggregation, Intrinsically disordered proteins

## Abstract

Abnormal trinucleotide repeat expansions alter protein conformation causing malfunction and contribute to a significant number of incurable human diseases. Scarce structural insights available on disease-related homorepeat expansions hinder the design of effective therapeutics. Here, we present the dynamic structure of human PHOX2B C-terminal fragment, which contains the longest polyalanine segment known in mammals. The major α-helical conformation of the polyalanine tract is solely extended by polyalanine expansions in PHOX2B, which are responsible for most congenital central hypoventilation syndrome cases. However, polyalanine expansions in PHOX2B additionally promote nascent homorepeat conformations that trigger length-dependent phase transitions into solid condensates that capture wild-type PHOX2B. Remarkably, HSP70 and HSP90 chaperones specifically seize PHOX2B alternative conformations preventing phase transitions. The precise observation of emerging polymorphs in expanded PHOX2B postulates unbalanced phase transitions as distinct pathophysiological mechanisms in homorepeat expansion diseases, paving the way towards the search of therapeutics modulating biomolecular condensates in central hypoventilation syndrome.

## Abstract (web summary)

Here, the authors show that pathogenic mutations in the polyalanine expansions of PHOX2B promote nascent structural conformations that trigger irreversible phase transitions that arrest wild-type PHOX2B, disrupting function.

## Introduction

Eukaryotic proteomes contain abundant stretches of sequentially repeated aminoacids or homorepeats, being polyglutamine, polyasparagine and polyalanine the most prevalent^1^. Although the function of homorepeats is still unclear, they are presumed to play roles in protein structure and interactomes, acting as polymorphic adaptors in a length- and boundaries-dependent manner^2^. Recombination between two mispaired alleles can cause expansions as well as contractions in homorepeats^3^. Indeed, the aberrant increments of consecutive glutamine residues by trinucleotide repeat expansion (TRE) mutations were linked to multiple human diseases more than 30 years ago^4^. Significantly less is known about polyalanine-containing proteins, which are enriched in transcription factors and RNA-binding proteins^2^. Expansions of the polyalanine segments above certain thresholds are involved in at least nine different human diseases, including developmental defects and neurological disorders^5^. Several mechanisms were proposed to explain the pathogenicity of TREs, such as protein de-regulation or loss-of-function, cellular mislocalization, dominant-negative effects or a toxic gain of function^4–6^. Considering that the structural conversions induced by the TREs have a profound impact in protein malfunction, intense research was dedicated to describe the structure of TRE mutants^7–10^. However, due to their challenging nature^10^, structural descriptions of homorepeat-containing proteins are currently scarce. Yet, it is essential to understand the molecular mechanisms of TRE-based diseases to develop efficient therapeutics^2^.

Congenital Central Hypoventilation Syndrome (CCHS or Ondine´s curse) is a rare neuropathy characterized by a decreased sensitivity to hypoxia and hypercapnia, which is paralleled by the inadequate control of breathing, particularly during sleep^11^. Additionally, CCHS shows impaired heart rate, blood pressure and body temperature regulation. CCHS is a chronic disorder affecting mainly children, with autosomal dominant transmission and has no effective treatment^12^. 90% of CCHS patients show mutations in the vital developmental transcription factor PHOX2B (Paired mesoderm homeobox protein 2B)^13,14^. PHOX2B is a master gene in charge of the development of autonomic nervous system regions responsible for medullary control reflexes of autonomic functions^12,15^. In particular, heterozygous in-frame duplications within a 20 alanine stretch located in the C-terminus of PHOX2B, leading to expansions from +4 to +13 alanine residues, being +5, +6 and +7 the most common, were associated with CCHS^13,14,16^. Remarkably, the length of the polyalanine expansions (also called polyalanine repeat mutations or PARMs) in PHOX2B correlates with the severity of the disease phenotype in CCHS^3,14,17^. In parallel, PHOX2B showed a polyalanine (polyAla) length-dependent cytoplasmic aggregation when overexpressed in culture cells^18–20^. Consequently, loss of PHOX2B transcription function due to its cytosolic aggregation has been postulated as a possible pathogenic mechanism in CCHS^5,12,18^. However, the role of PHOX2B aggregates in neuronal failure in CCHS remains controversial, since cytoplasmic deposits are only observed upon high overexpression of PHOX2B carrying the longer PARMs^18,21^ and is not observed in a knock-in mouse carrying a + 7 PARM^22^. Alternatively, polyAla tracts were shown to form abnormal soluble oligomers in vitro^1^ and in cells^23^, and a recent study demonstrated that PARMs in the paralog protein HOXD13 cause hereditary synpolydactyly in humans due to altered liquid-liquid phase separation (LLPS)^24^. Interestingly, protein misfolding within membraneless compartments formed by phase separation and percolation^25^ can overpower the refolding capacity of molecular chaperones, triggering liquid-to-solid transitions in aged condensates which could be the basis for protein loss-of-function in degenerative diseases^26^. Therefore, unbalanced phase transitions due to proteostasis misregulation could be a key molecular process triggering PHOX2B malfunction in CCHS, by means of a conformational pathway in PHOX2B that would be preferentially routed by the longer PARMs. Hence, the structural polymorphism induced by the PARMs could promote nascent misfolded conformations in PHOX2B that prompt liquid-to-solid transitions in the cytosol^27,28^. How the PARMs, or even the TREs in general, promote protein misfolded conformations that elude chaperone control, remains a mystery.

The highly repetitive nature of homorepeats makes them particularly challenging for structural studies. Still, Nuclear Magnetic Resonance (NMR) spectroscopy is the technique of choice to characterize the structure of homorepeats, due to their inherent dynamics. However, only limited structural information has been obtained so far for polyglutamine peptides by NMR^9,10^. Besides a central DNA-binding homeobox domain, sequence analysis reveals flanking transcriptional activation regions composed of disordered segments and putative structured motifs in PHOX2B. Therefore, the large disordered regions and the particular content of aromatic residues could contribute to PHOX2B de-mixing in the cell^25^. Liquid-liquid and liquid-solid phase transitions are expected to account with protein structural rearrangements towards β-sheeted structural motifs which self-assemble through steric zipper-type stickers^27–32^. However, proof of structural polymorphism within condensates is scarce^28^, and several lines of evidence proposed that proteins remained mainly disordered and dynamic in the dense phases^33–35^.

Here, we present the solution structure of the C-terminal domain of human PHOX2B containing the longest polyAla fragment known in mammals (20 consecutive alanines). Pathogenic PARMs relevant in CCHS promote nascent structural conformations that trigger fast phase transitions into solid condensates that arrest wild-type PHOX2B. Remarkably, these incipient conformations are precluded upon chaperone recognition, which blocks PHOX2B de-mixing. We propose that fast phase transitions promoted by PARMs disrupt PHOX2B function, and advance that preventing the nascent polymorphism could restore the normal function of expanded PHOX2B in CCHS.

## Results

### PHOX2B polyAla tract populates a rigid α-helix in solution

**Fig. 1:**
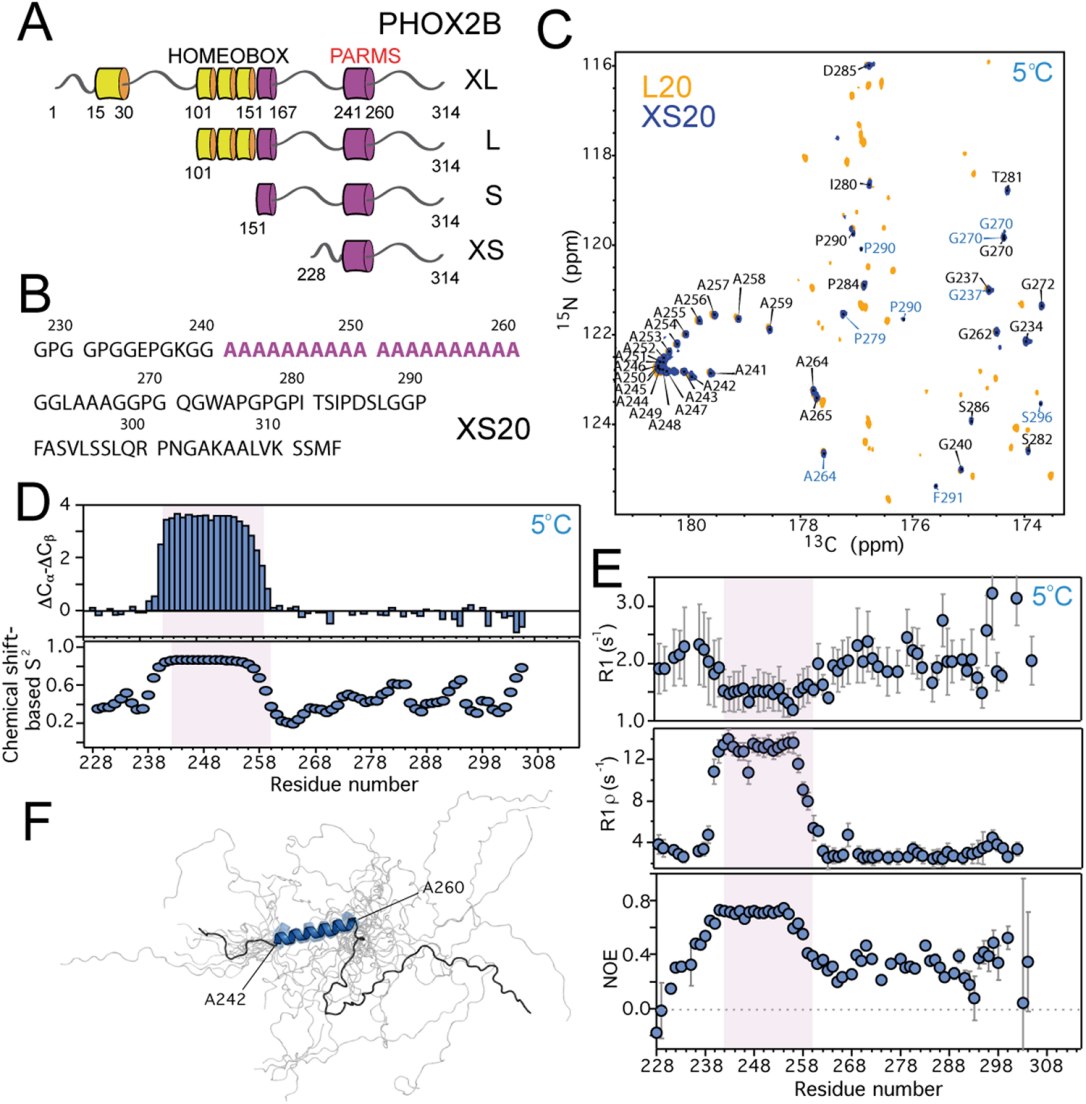
PHOX2B polyAla forms a stable α-helix in solution. **A** Different constructs containing PHOX2B domains. α-helical regions are represented by cylinders, and the polyAla regions colored in purple. PARMS are located in the polyAla segment covering residues 241–260. **B** XS20 primary structure. The polyAla is highlighted in purple. **C** Overlaid CON NMR spectra for L20 (orange) and XS20 (blue) at 5 °C. Assignments for XS20 are shown. Blue assignments correspond to alternative isomers. **D** Secondary chemical shifts (top) and chemical shift-based S^2^ order parameters (bottom) for XS20 at 5 °C. **E** ^15^N spin relaxation parameters for XS20 at 5 °C. Error bars were calculated by fitting an exponential decay to the data. **F** Structural ensemble of the 20 XS20 conformers with the lowest conformational energy at 5 °C. Structures were aligned onto the structured elements. The boundaries of the α-helix (in blue) are indicated. The purple shadows in the plots in **D** and **E** limit the polyAla region. Protein concentration was 0.5 mM. Source data are provided as a Source Data file.

The structural changes induced by the PARMs in PHOX2B may alter PHOX2B´s interactome and transcription function, leading to protein malfunction. We employed solution NMR spectroscopy to understand the structural impact of PARMs in PHOX2B. The polyAla tract present in the C-terminal domain of human PHOX2B is singularly conserved among mammals, ranging from 16 consecutive alanines in horse to 21 in cat. Sequence analysis and structural prediction reveal short structured elements flanked by long disordered regions in human PHOX2B (Fig. 1A, B). Several constructs of PHOX2B were generated containing different protein regions to enhance solubility and minimize spectral complexity (Fig. 1A). We focused on the fragment 228-314 (termed XS20, for the number of consecutive alanines contained in the tract) of PHOX2B because its NMR spectra mirrored that of the longer PHOX2B fragments (Fig. 1C). Production of high-purity, highly soluble samples enabled us to acquire 3D NMR experiments on XS20 which follow backbone connectivities with high spectral resolution (Methods), circumventing the significant signal overlap from the polyAla repetitive region and enabling us to obtain unambiguous assignments (Fig. 1C, Supplementary Fig. 1A).

The polyAla region (residues 241–260, Fig. 1B) displayed a remarkable arrangement in the 2D CON NMR spectra (Fig. 1C), where the consecutive alanine moieties mostly follow an N-to-C termini trail. NMR secondary chemical shift analysis and chemical shift-derived S^2^ order parameters calculated by TALOS-N^36^ indicate that the polyAla tract particularly populates α-helical conformations, while the rest of the protein remains largely disordered (Fig. 1D). J-coupling analysis showed values close to 4 Hz for the polyAla tract (Supplementary Fig. 1B), which is in agreement with a strong tendency to adopt α-helical structures^26^. Close inspection of the spectra reveals multiple minor conformations in the disordered regions due to significant *cis-trans* isomerization of the Pro residues in the numerous GPG motifs of PHOX2B (Fig. 1B, Supplementary Fig. 1A, C). ^15^N spin relaxation experiments for XS20 at 5 °C show a significant deviation of the longitudinal (*R*_1_) and rotating-frame (*R*_1ρ_) relaxation rates and the heteronuclear nuclear Overhauser effect (NOE) values for the polyAla region, indicating local rigidity due to the formation of secondary structure (Fig. 1E). The C-terminal region of the polyAla fragment appears less rigid, which would agree with the lower secondary chemical shift values observed (Fig. 1D). Overall correlation time (τ_C_) obtained from polyAla relaxation parameters^37^ (6,7 ns, Supplementary Table 1) indicates that XS20 may establish intermolecular associations through the polyAla segment at 5 °C.

A significant set of ^1^H-^1^H NOE contacts were observed in ^1^H-^15^N and ^1^H-^13^C heteronuclear NOESY experiments which, in combination with chemical shifts and torsion angle restraints, defined distance restraints for XS20 structure calculation (Supplementary Table 2). A set of 100 conformers were calculated by CYANA, and the 20 conformers with the lowest target function were selected and assessed by PROCHECKNMR (Fig. 1F). Almost the whole polyAla segment is contained in the α-helix (residues 242-260), which shows a significant structural convergence within all the conformers assessed (RMSD of 1.41 Å, Supplementary Table 2). The rest of XS20 is highly dynamic, in close agreement with the chemical shifts, J-couplings and relaxation data (Fig. 1D, E, Supplementary Fig. 1B).

### PHOX2B undergoes a temperature-dependent de-mixing

**Fig. 2:**
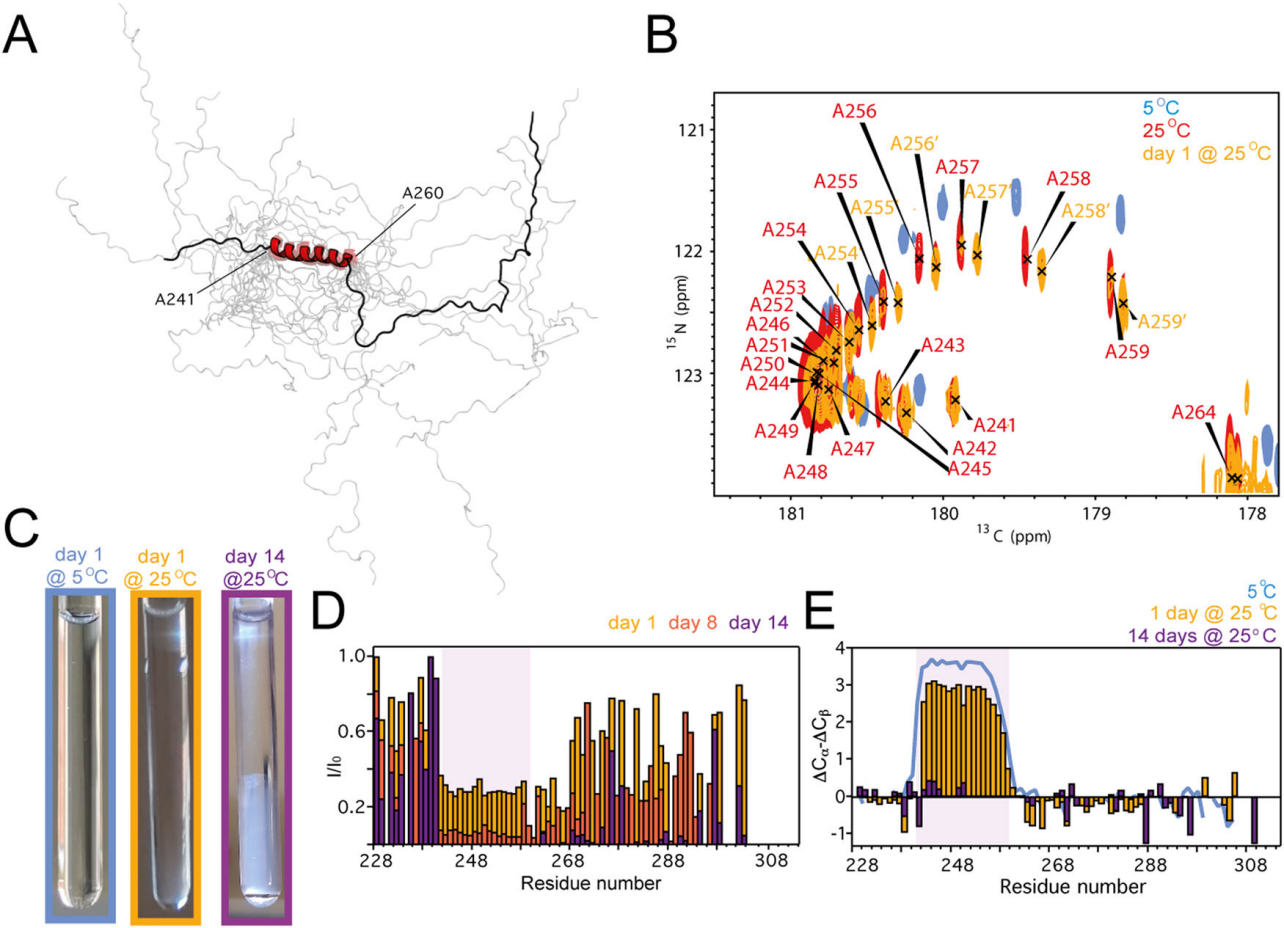
PHOX2B polyAla is coupled to temperature-dependent phase transitions. **A** Structural ensemble of XS20 20 lowest energy conformers at 25 °C. The lowest energy structure is displayed on top of the ensemble. Boundaries of the α-helix (in red) are indicated. **B** Overlaid CON spectra for XS20 α-helical region at 5 °C (blue), 25 °C (red) and after 1 day at 25 °C (orange). Alternative conformations appearing upon incubation are labeled in orange. This region disappears with longer incubation times. **C** XS20 NMR samples following the indicated incubations. **D** Decay in intensity plot for XS20 at day 1 (orange), 8 (dark orange) and 14 (purple) of incubation at 25 °C. **E** Secondary chemical shift plots for XS20 at 5 °C (blue line), and after 1 day (orange bars) and 14 days (purple bars) of incubation at 25 °C. Purple shadows in **D** and **E** limit the α-helix. Protein concentration was 0.5 mM. Source data are provided as a Source Data file.

The solution structure of XS20 at 25 °C was also calculated on the basis of experimental restraints (Fig. 2A, Supplementary Table 3). The structure presents a well-delimited α-helix, covering the entire polyAla segment, flanked by highly disordered regions. Remarkably, the CON NMR spectra revealed the appearance of secondary conformations in XS20 samples upon incubation for 1 day at 25 °C, particularly evident in the C-terminal region of the polyAla α-helix (Ala255-Ala259; Fig. 2B). These conformations showed lower values in the CO dimension, indicative of decreased helical content^38^. In addition, continued incubation of XS20 at 25 °C promoted turbidity in the NMR samples (Fig. 2C). Analysis of the ^15^N HSQC NMR spectra for the incubated XS20 samples indicated significant signal decay, with further intensity decay in the polyAla α-helix (Fig. 2D, Supplementary Fig. 2). Because an increase in turbidity is associated with protein de-mixing or aggregation processes^39^, we wondered whether the NMR signal decay observed in the polyAla α-helical segment was due to intermolecular associations involved in PHOX2B multimerization or rather to structural conversions coupled to phase transitions^28^. Analysis of the secondary chemical shifts indeed revealed that incubated XS20 showed a time-dependent decrease in the α-helical content of the polyAla region (Fig. 2E), which suggests that PHOX2B displays metamorphism towards disordered structures upon de-mixing or aggregation. Line broadening analysis and diffusion NMR revealed that the observable NMR moieties (Fig. 2B) belong to species of similar assembly order, since large oligomer assembly lay NMR signals beyond detection (Supplementary Fig. 3A, C). Importantly, XS20 showed no significant degradation during long incubations at 25 °C (Supplementary Figs. 3 and 4). Therefore, increase in order parameters or protein degradation are not responsible for the deepened signal decay detected in the polyAla α-helix upon incubation (Fig. 2D), but it is rather due to slow conformational exchange towards disordered conformations (Fig. 2B, E).

**Fig. 3:**
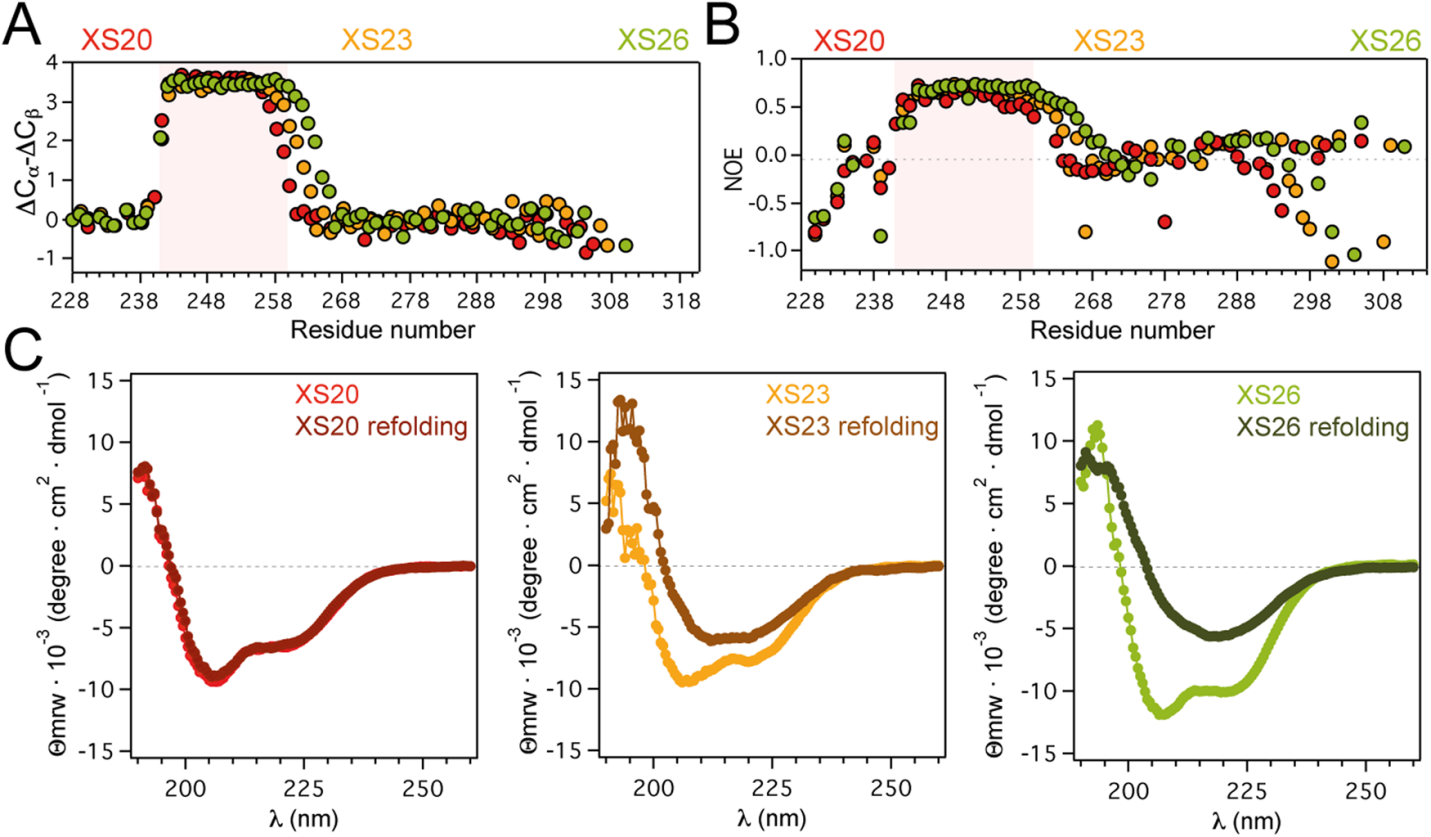
PARMS extend the α-helix promoting irreversible unfolding. **A** Secondary chemical shifts for XS20 (red), XS23 (orange) and XS26 (green) at 25 °C. **B** ^15^N heteronuclear NOEs for XS20 (red), XS23 (orange) and XS26 (green) at 25 °C. Errors bars (<0.4) are not included for simplicity. The red shadows in the plots in **A** and **B** limit the α-helix in XS20. **C** CD spectra at 25 °C for XS20 (red), XS23 (orange) and XS26 (green) before and after heat denaturing at 95 °C (darker spectra in the plots). Protein concentration was 0.5 mM (**A**, **B**) and 0.017 mM (for XS23) and 0.036 mM (for XS20 and XS26) in **C**. Source data are provided as a Source Data file.

J-coupling assessment and chemical shift-based order parameters confirmed that the structure of XS20 is largely identical at 5 °C and 25 °C (Supplementary Fig. 5). Moreover, the ^15^N spin relaxation parameters for XS20 at 25 °C indicate that the protein maintains the main rigid element covering the polyAla segment, with increased flexibility in the C-terminal region of the α-helix (Fig. 3A, B, Supplementary Fig. 6). τ_C_ calculations based on experimental relaxation data indicates that XS20 may also self-assemble through the polyAla tract at 25 °C (Supplementary Table 1). Remarkably, these self-assembly and structured patterns are maintained at considerably lower protein concentrations, (0.05 mM, Supplementary Fig. 6), revealing that XS20 in solution is not monomeric and displays time-dependent assembly into large oligomers at high protein concentrations (Supplementary Fig. 3). Considering that the major structures of XS20 at 5 °C and 25 °C are fairly identical (Supplementary Fig. 5C, D), the remarkable propensity of PHOX2B to self-associate into large oligomers or aggregates upon incubation at higher temperatures could be attributed to the increased contact potential of hydrophobic Ala sidechains at 25 °C^40^ or to temperature-dependent structural rearrangements that trigger multimerization.

### PARMs extend the major polyAla α-helical conformation

Pathogenic PARMs are expected to alter protein conformation impacting interactomes^5^. To determine the effect of PARMs on PHOX2B structure, we generated a + 3 Ala variant (adding 3 alanine residues to the 20 polyAla tract, named XS23) and a + 6 Ala variant (named XS26). While a + 3 Ala expansion would not be strongly correlated to CCHS, XS26 would be representative of the most abundant PARMs observed in the disease^13,14,16^. NMR secondary chemical shifts showed that the Ala inclusions in XS23 and XS26 extend the α-helix covering the polyAla segment (Fig. 3A). In addition, ^15^N spin relaxation data indicated that the expanded α-helices from XS23 and XS26 are also rigid (Fig. 3B) and show a stronger propensity to establish length-dependent intermolecular associations at 25 °C, particularly evident for XS26 (Supplementary Figs. 3, 7, Supplementary Table 1). Therefore, PARMs do not alter the structural and dynamic properties of the major α-helical conformation of the polyAla tract but increase its self-assembly potential.

At low temperatures, polyAla repeats can form coiled-coil structures that increase in stability with polyAla length^7^. However, PARMs were also reported to decrease PHOX2B thermal stability favoring amyloid aggregation^6^. Circular dichroism (CD) measurements determined that both XS20 and the expanded variants display a high content in α-helical structure (Fig. 3C). However, spectral analysis revealed no indication of coiled-coil formation for any PHOX2B variant at 25 °C, in agreement with previous data obtained on model polyAla sequences^7^. Strikingly, although XS20 showed a fully reversible refolding after heat unfolding, both XS23 and XS26 showed irreversible unfolding. Indeed, the spectra revealed an α-to-β switch similar to that reported elsewhere for designed peptides^41^. Therefore, we wondered whether PARMs modulate the equilibrium between conformational states giving rise to nascent conformations which could promote associations into higher order species.

### PARMs promote alternative minor conformations

**Fig. 4:**
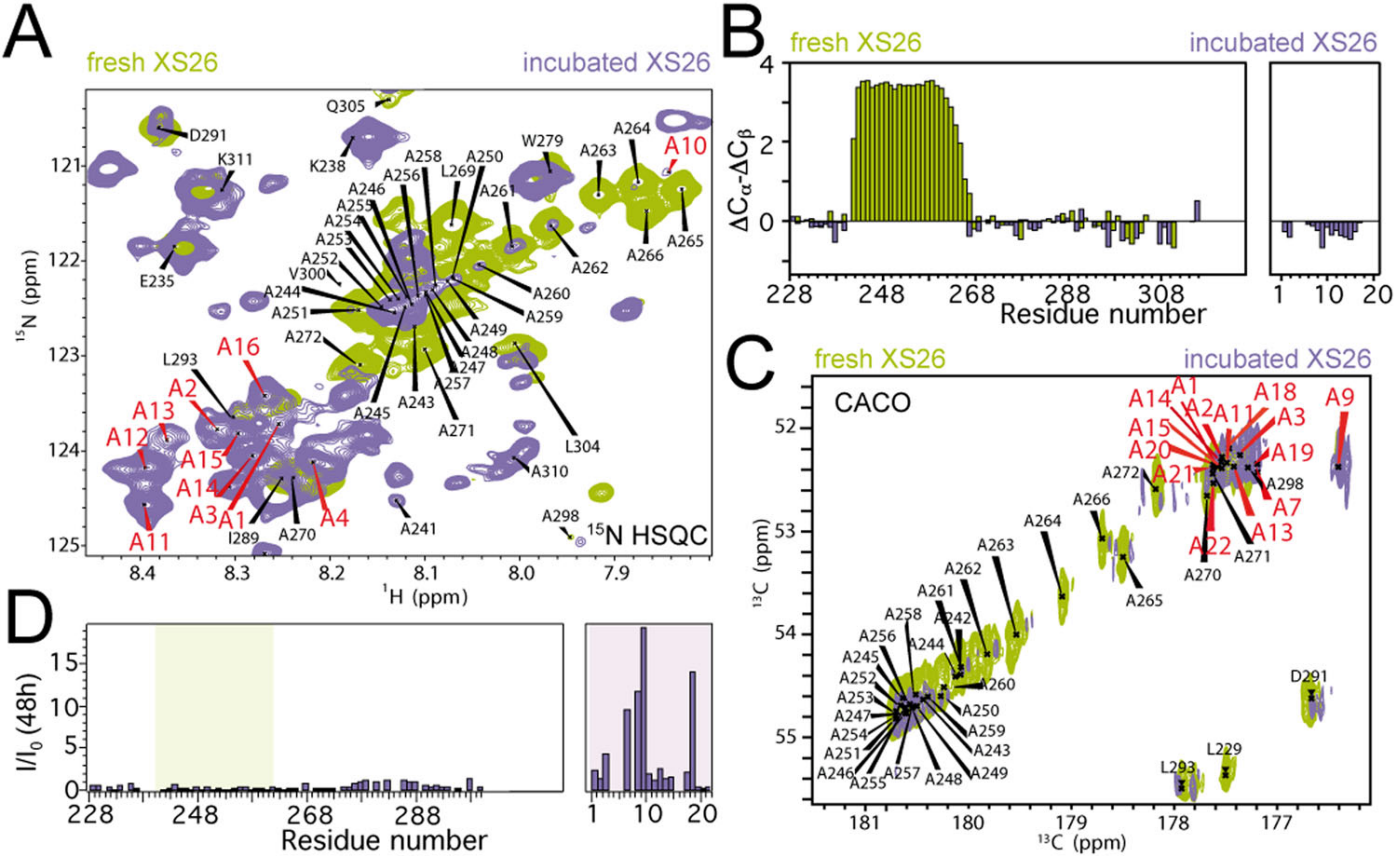
PARMs promote nascent conformations. **A** Detailed ^15^N-HSQC spectra at 25 °C for fresh XS26 (green) and XS26 incubated 7 days at 25 °C (navy). **B** Secondary chemical shifts for fresh XS26 (green) and XS26 incubated 7 days at 25 °C (navy). Values for new alanine moieties are plotted on the right. **C** Detailed CACO spectra at 25 °C for fresh XS26 (green) and XS26 incubated for 2 days at 25 °C (navy). **D** CACO signal intensity for XS26 after 2 days at 25 °C. Green shadow limits XS26 polyAla α-helix, while purple shadow limits nascent disordered alanine moieties (plotted on the right). Nascent alanine moieties (red in **A** and **C**) follow identical numbering and are plotted correlatively in **B** and **D**. Protein concentration was 0.5 mM in **A** and **B** and 0.225 mM in **C** and **D**. Source data are provided as a Source Data file.

The 2D CON spectra showed shifts towards higher values in the CO dimension for the expanded polyAla fragments, which is indicative of a stronger tendency to populate α-helical conformations (Supplementary Fig. 8). However, incubation of XS26 and XS23 at 25 °C promoted considerable sample turbidity, triggering significant changes in the ^15^N HSQC NMR spectra (Fig. 4A). Besides the disappearance of the polyAla α-helical region, as described for XS20 after prolonged incubations (Supplementary Fig. 2), new crosspeaks appeared in the spectra which were not attributable to protein degradation or large oligomer assembly (Supplementary Figs. 3, 4), but rather to slow conformational exchange in low assembly order species. Sequential assignments corroborated that the new crosspeaks belong to Ala residues which display disordered conformations, clearly identified by the characteristic chemical shifts for alanine´s Cα and Cβ moieties^38^ (Fig. 4A, B). The signal intensity of these new Ala resonances increase with the incubation time, and is highly reproducible among different protein batches (Supplementary Fig. 9). ^15^N spin relaxation measurements for the incubated samples confirmed that the new polyAla crosspeaks are highly dynamic, in stark contrast with the major, rigid α-helical conformation for the polyAla segment in fresh samples (Supplementary Fig. 10, Supplementary Table 1).

In addition, comparison of the 3D NMR CBCA(CO)NH spectra for XS20, XS23 and XS26 showed that the region where central polyAla crosspeaks appear additionally contained emerging crosspeaks with chemical shifts which are characteristic for alanines in disordered (or random coil; 52,67+/− 1,76 ppm) or β-strand (50,86+/− 1,28 ppm) conformations^38^ (Supplementary Fig. 11). Remarkably, only the expanded XS23 and XS26 variants showed the nascent crosspeaks, with higher signal intensity for XS26. However, strong signal overlap in this region of the spectra could lead to dubious spectral processing. To discard misassignment due to artifactual spectral processing, the presence of the alternative conformations in the PARMs was validated in 2D NMR spectra with direct ^13^C detection (CACO spectra), in addition to 2D ^13^C HSQC spectra (Fig. 4C, Supplementary Fig. 12). CACO spectra provided also direct evidence of the emergence of polyAla disordered conformations upon incubation (Fig. 4D). Therefore, PARMs promote alternative minor conformations in PHOX2B, which could be the basis for their reported aggregation propensity^18–20,42^. However, a direct correlation between the abundance of the alternative polyAla conformations in PHOX2B and its propensity to aggregate, is missing.

### Pathogenic PARMs trigger rapid phase transitions

**Fig. 5:**
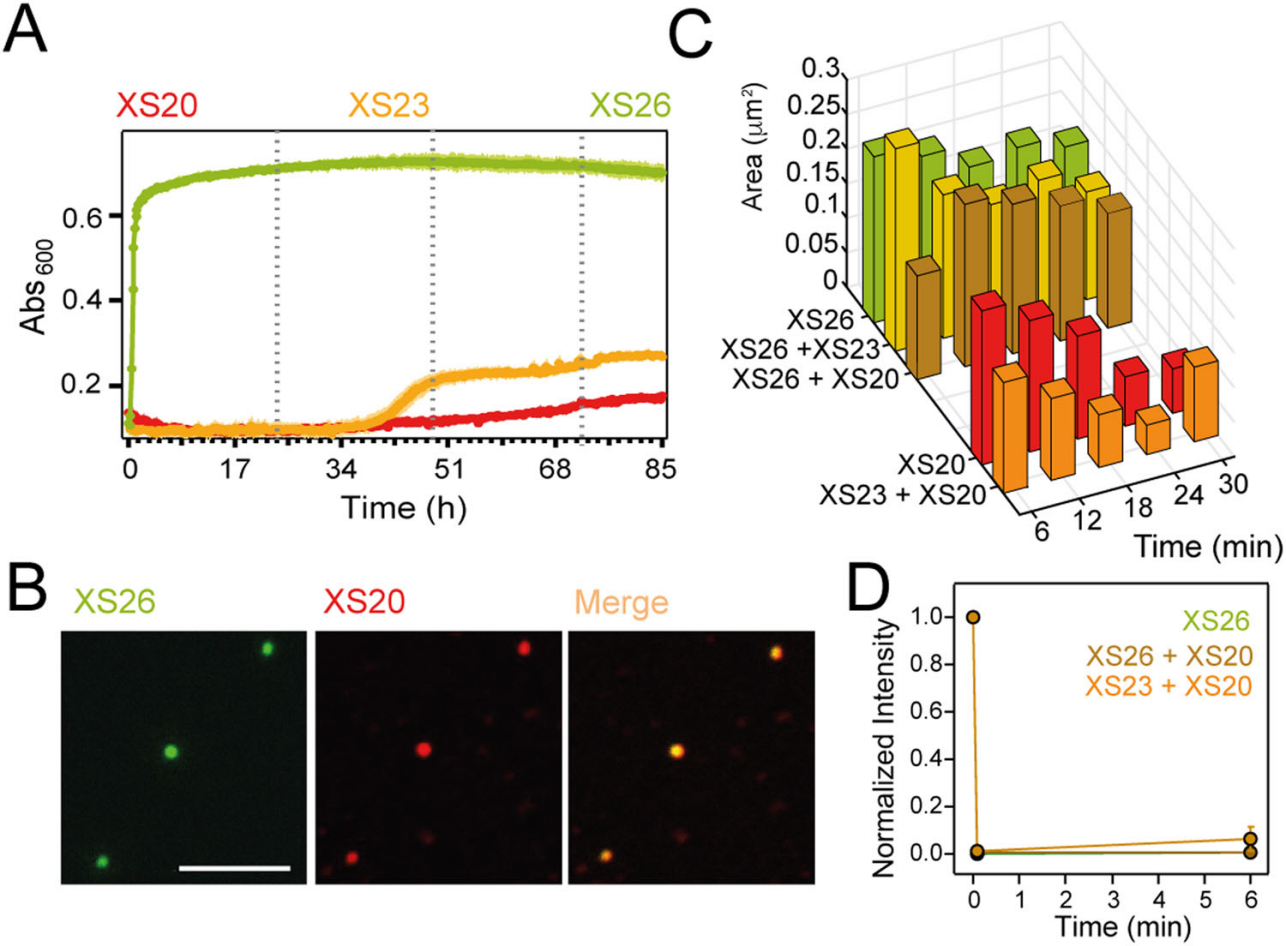
Pathogenic PARMs promote rapid phase transitions. **A** Turbidity at 25 °C for 0.225 mM XS20 (red), XS23 (orange) and XS26 (green). Broken lines represent consecutive days of incubation. **B** Confocal fluorescent microscopy images showing co-localization of XS20 in XS26 condensates. XS26 was labeled with ATTO-488 (green) and XS20 with ATTO-565 (red). Scale bar: 5 μm. Data were biologically reproduced at least three times (see Supplementary Fig. 13). **C** Histogram distribution of the area (in μm^2^) of the condensates formed by the protein mixtures with respect to the time spent for the microscopic observations. Error bars (<0.3 in μm^2^) are not included for clarity. **D** TIRF-FRAP data for XS26 condensates (green), and for mixtures of XS26 + XS20 (brown) and XS23 + XS20 (orange) incubated for 6-18 minutes at 25 °C. FRAP data obtained at different incubation times was combined. Condensates showed marginal fluidity in all conditions tested and no fluorescence was recovered after bleaching. n_XS26_ = 64; n_XS26+XS20_ = 50; n_XS23+XS20_ = 16 (*n*: number of condensates). Data are presented as mean values +/−SD. Samples in **B**–**D** contained 0.1 mM XS23 and XS26 and 0.15-0.25 mM XS20. Source data are provided as a Source Data file.

Light dispersion measurements showed a length-dependent increase in turbidity for PHOX2B, with a rapid response for XS26 (Fig. 5A). Confocal microscopy confirmed that XS26 promoted phase de-mixing rather than fibrillar aggregation^42^ (Fig. 5B). Interestingly, the condensates triggered by XS26 de-mixing incorporated XS20, which correlates with the dominant negative effect of the pathogenic PARMs in PHOX2B^18,19,21^. Fluorescence microscopy validated the minimal de-mixing for XS20 (Supplementary Fig. 13, Supplementary Fig. 14A). In addition, XS20 is arrested into XS26 condensates at a slower rate (Supplementary Fig. 13), in agreement with the reported slower kinetics of aggregation for the shortest PHOX2B variants^6^ (Fig. 5A, Supplementary Fig. 15).

A recent study reported that PARMs in the protein HOXD13 alter its de-mixing properties and its capacity to co-condense with transcriptional co-activators, presenting dysregulated phase separation as the potential basis for human synpolydactyly^24^. To determine the phase transition properties of XS26, we performed fluorescent microscopy experiments on individual condensates (Fig. 5C, D). Recruitment of XS20 into heterotypic condensates promoted a time-dependent reduction in condensate size and fluorescence intensity (Fig. 5C, Supplementary Fig. 14B), which is indicative of lower protein content due to diffusion in the aged condensates containing XS20. The considerable small size of PHOX2B condensates (<1 μm, Fig. 5B, C, Supplementary Fig. 13, Supplementary Fig. 14C) makes the acquisition of fluorescent recovery after photobleaching (FRAP) measurements by confocal microscopy highly challenging (Supplementary Fig. 16). Thus, FRAP measurements were performed on total internal reflection fluorescence (TIRF). TIRF-FRAP rates revealed minimal mobility inside the homotypic and heterotypic PHOX2B condensates irrespective of the incubation time (Fig. 5D), consistent with confocal-based FRAP measurements (Supplementary Fig. 16). Therefore, PARMS promote a rapid transition into solid condensates, which can retain wild type PHOX2B. Interestingly, concentration-dependent PHOX2B phase transitions are not affected by increased ionic strength (Supplementary Figs. 15, 17), suggesting that, while hydrophobicity may promote small oligomers via polyAla coiled coils at low temperatures^7^, phase de-mixing is not driven by polyAla´s hydrophobicity. However, increasing temperatures accelerated phase transition kinetics (Supplementary Figs. 15, 17B), which may be explained by enhanced thermal destabilization of polyAla major helical conformation^6^. Therefore, we wondered whether the structural disorder induced by PARMs is coupled to phase transitions^28^.

### Chaperones arrest PHOX2B nascent conformations

**Fig. 6:**
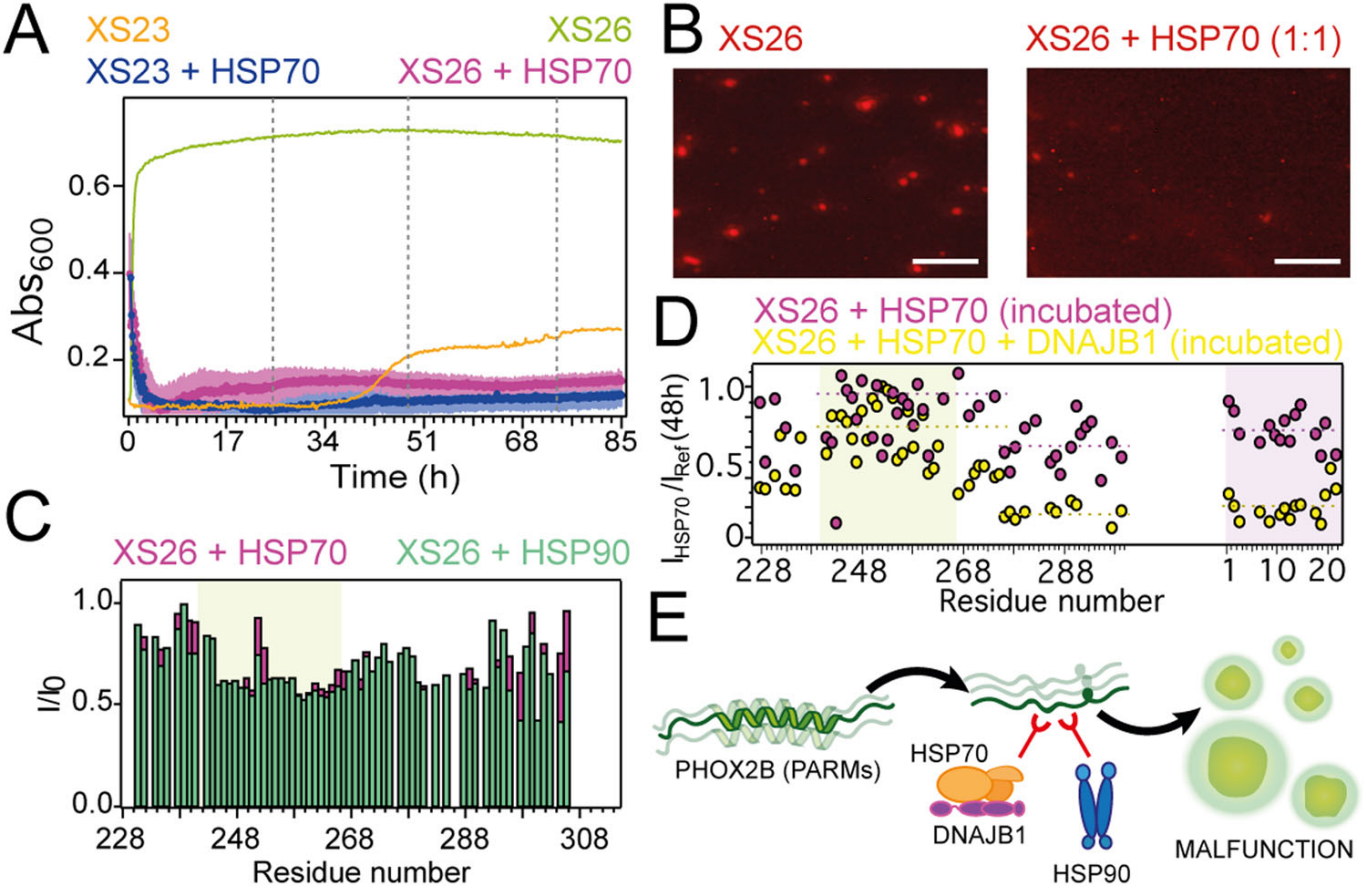
Chaperones suppress PHOX2B de-mixing arresting nascent conformations. **A** Turbidity at 25 °C for 0.225 mM XS23 or XS26 in presence of 0.1 mM HSP70 (blue and magenta, respectively). Plots for the unaccompanied PHOX2B constructs are included. **B** Fluorescence microscopy images for XS26 (0.1 mM, labeled with ATTO-565) in the absence and presence of HSP70. Scale bars = 10 μm. Data were biologically reproduced three times. **C** ^15^N-HSQC-based signal intensity plots for XS26 major conformation (0.07 mM) in complex with HSP70 (magenta) and HSP90 (green), all in 1:2 molar ratios. Green shadow limits polyAla α-helix. **D** XS26 (0.225 mM) CACO signal intensity decay upon incubation (2 days at 25 °C) in presence of HSP70 (1:2 equivalents, in magenta) and HSP70:DNAJB1 (1:2:1 equivalents, in yellow). Green shadow limits polyAla α-helix, while purple shadow limits nascent disordered alanine moieties (plotted on the right). Broken lines mark the average intensity for the different segments. **E** PARMs promote disorder in PHOX2B polyAla segment triggering fast phase transitions. Chaperones arrest nascent disordered conformations blocking phase separation. Source data are provided as a Source Data file.

Molecular chaperones are essential for neutralizing protein misfolding and malfunction. Heat shock proteins HSP70 and HSP40 co-localize with cellular PHOX2B aggregates, and are directly involved in their clearance^19,43,44^. Turbidimetry and fluorescence microscopy revealed that HSP70 minimized XS26 phase transitions (Fig. 6A, B). Because phase transitions are coupled to rearrangements within the polyAla α-helix (Figs. 2–4), we reasoned that chaperones diminish PHOX2B homo-associations by binding the polyAla α-helix in high affinity. This hypothesis would agree with the chaperone capacity to recognize hydrophobic, structured motifs in disordered clients^26^. Surprisingly, NMR titrations showed that HSP70 and HSP90 molecular chaperones are not preferentially bound to PHOX2B polyAla α-helix (Fig. 6C). However, since there is a patent correlation between the emergence of polyAla alternative conformations and the propensity to phase separate by PHOX2B (Figs. 4 and 5), we considered that chaperones could target specifically polyAla alternative conformations rather than the major α-helical structure. 2D ^13^C HSQC and CACO spectra provide convenient templates to confirm this specific recognition because they expose the whole diversity of conformations in the polyAla tract and are short time consuming. Moreover, because chaperone binding will result in signal broadening beyond detection^26^, increased difference in signal intensity in presence of chaperones is an indication of preferential chaperone targeting. Analysis of signal intensity of the different polyAla conformations in XS26 revealed that HSP70 and HSP90 molecular chaperones predominantly target disordered conformations (Fig. 6D, Supplementary Fig. 18). In brief, the signal intensity of XS26 polyAla tract in α-helical conformation diminishes up to 20% in presence of freshly added HSP70 and 40% for HSP90, while the intensity of the polyAla moieties in disordered conformation are reduced above 50% in the presence of chaperones (Supplementary Fig. 18). In addition, the population of alternative disordered alanines that emerge upon incubation are selectively targeted by HSP70, and diminish about 30 % after 48 h of incubation (Fig. 6D). Remarkably, addition of the HSP40 co-chaperone DNAJB1, which is known to target disordered clients^26^, significantly blocked 80% of the population of XS26 disordered polyAla conformations (Fig. 6D). To confirm that polyAla disorder promote phase transitions, we generated a Gly-Pro mutant to induce increased disorder in PHOX2B´s polyAla α-helix (Supplementary Fig. 19). The Gly-Pro mutant (termed XS_GP_) disrupts polyAla α-helix increasing disordered conformations, which significantly accelerates phase transition kinetics. Therefore, the data clearly indicate that chaperones target disordered nascent conformations induced by PARMs, which are the template structures priming phase transitions^28^(Fig. 6E).

## Discussion

About 15% of eukaryotic proteins contain homorepeats^2^. Polyalanine, polyserine and polyglutamine segments primarily adopt α-helical conformations^2^, reportedly promoting coiled coil associations triggering length-dependent aggregation^7^. Interestingly, polyAla coiled coils showed a higher tendency to aggregate compared to polyQ and polyS^45^, explaining why polyAla homorepeats are usually shorter and less polymorphic than polyQ repeats^46^ and display lower thresholds for disease-causing TREs^47^. PHOX2B contains the longest polyAla segment known in mammals and one of the shortest thresholds for disease-causing expansions^13,19^. Long PARMs in PHOX2B cause shifts in localization and cytoplasmic aggregation^18–21^. Therefore, a loss-of-function mechanism triggered by aggregation has been postulated as the basis for mutated PHOX2B malfunction in CCHS^5,12,18^. Similar mechanisms were proposed for the impact of PARMs in SOX3, RUNX2, FOXL2, HOXA13 and HOXD13^43,48,49^. However, clear connections between expanded homorepeats aggregation and cellular toxicity are still missing^5^. In addition, the nature of PARM aggregates remained controversial, since both amyloid^42^ and α-helical-rich aggregates^23^ have been described for polyAla fragments. Most of the PARMs are related to the origin of human developmental diseases, which would be in agreement with PARM-induced protein chronic dysfunction, rather than with amyloid aggregation^23^. Indeed, lack of detection of PHOX2B aggregates in mouse models^22^ is consistent with the assumption that PARMs may cause toxicity triggering aberrant oligomers^23,50^. Frameshift mutations in PHOX2B promote nucleolar mispartitioning and cytotoxicity by aberrant phase transitions^51^. In addition, PARMs in HOXD13 dysregulate LLPS, favoring homotypic phase separation and reducing condensate fluidity^24^. This aberrant de-mixing lessens the blending of HOXD13 with transcription factors in heterotypic condensates, potentially inhibiting transactivation and ultimately promoting human synpolydactyly. Our data indicate that a similar mechanism could underly expanded PHOX2B loss-of-function in CCHS (Supplementary Fig. 20). Pathogenic PARMs in PHOX2B trigger fast phase transitions into small, solid condensates which seclude the wild-type form. Loss of PHOX2B function is therefore previous to aggregation into large protein deposits, consistent with the lack of protein aggregation in mice models of human CCHS^22^. Still, PHOX2B condensates evolve into irregular clusters (Supplementary Fig. 13, Supplementary Fig. 17C, D) rather than fibrillar aggregates^23^, explaining why expanded PHOX2B is deposited in the cell cytoplasm upon over-expression^18,21^. These features are also present in other PARMs, in agreement with a common mechanism underlying polyAla human diseases^5,23,24^. Interestingly, a recent report revealed cytosolic condensates triggered by polyS motifs which recruit and promote tau aggregation^52^. Therefore, condensate formation could represent distinct targets for therapeutic intervention in TRE human diseases, towards the discovery of compounds that modify the properties or prevent biomolecular condensates^24,51^.

Basu and colleagues showed that condensate fluidity decreased dramatically with the length of the expanded PARM^24^. In particular, 23-alanine HOXD13 showed minimal fluidity, and 25-alanine HOXA13 and 27-alanine RUNX2 showed absence of FRAP, indicating the solid properties of the condensates formed. Here, we observed fast solid condensate formation in PHOX2B promoted by a + 6 alanine expansion (26 alanines). This reduced condensate fluidity is not alleviated by the recruitment of wt PHOX2B (20 alanines) into the condensates. Interestingly, the solid condensates formed by a + 3 alanine expansion showed a remarkable delayed phase transition. Because a + 3 PARM in PHOX2B is not significantly related with CCHS, we could postulate that fast phase transitions triggered by long PARMs would promote protein malfunction, and solid condensates should therefore be considered the relevant therapeutic targets. Similar to HOXD13^24^, malignancy of PHOX2B condensates could result from the absence of additional partners in heterotypic condensates due to the fast de-mixing of expanded PHOX2B. Indeed, because of its ability to directly detect the abundance of alternative conformations responsible for phase transitions, NMR spectroscopy is a useful screening platform for structure-based design of phase transitions inhibitors. Importantly, because imbalanced phase transitions could well be the basis for protein misfunction in PARMs^24,51^, the structural approach presented here could be useful to target several human diseases. Yet, revealing the protein structures and interactions that drive aberrant phase transitions is indeed highly advantageous to design compounds that decrease the population of these active states.

Here, we report PHOX2B C-terminal atomic structures including the polyAla fragments, and reveal that PARMs alter the conformational transitions promoting nascent structures. Previous studies detected conformational changes induced by homorepeat expansions^53^ which could be modulated by the flanking regions and protonation states^8^. Indeed, flanking sequences modulate conformational transitions and aggregation propensities in homorepeats^2,9^. However, a direct observation of the co-existence of different conformations in expanded homorepeats in steady state was lacking. Taking advantage of the increased NMR signal intensity of the core of the polyAla tract moieties, in addition to the characteristic alanine chemical shifts, we observed the emergence of alternative conformations in PARMS coupled to phase transitions. In brief, we detected abundant conversions from α-helical to disordered conformations for the polyAla segment, in agreement with measurements of distant proteins in the dense phases^33–35^. This increased disorder in de-mixed phases is also in agreement with a recent study that described the different compaction of protein chains in the dilute and dense phases^28^. We additionally observed β-strand conformations, which appear exclusive for expanded PHOX2B. This α- to β-sheet interconversion by peptide plane flipping, postulated as a key step in amyloid formation^54^, has never been observed in condensed milieu to date. Intriguingly, despite the established relevance of β-sheet structures in protein deposits^29–32,55^, formation of polyAla β-sheet aggregates have only been observed under extreme experimental conditions^1,23,42,56^. Indeed, whereas the abundance of β-strand conformations correlates with the affluence and persistence of expanded PHOX2B condensates, remains to be established.

Phase separation predictors suggest that the regions flanking PHOX2B polyAla tract (rich in GPG motifs) show high tendency to phase separate (stickers^25^), while the hydrophobic polyAla tract would act as a spacer^25^. Therefore, it follows that conformational changes and increased disorder in the hydrophobic polyAla spacer would facilitate contacts within the stickers in the dense phases^28^. This is somehow in disagreement with the premise stating that polyAla expansions trigger protein aggregation through coiled coil stabilization^7^, but is in accordance with the role of disordered polyQ segments supporting Whi3 phase separation^57^. While coiled coils are prevalent in proteins that phase separate, no evidence for coiled coil formation in the polyAla tract was inferred from our CD data. Interestingly, NMR relaxation data showed that polyAla segments trigger small oligomeric assembly regardless of the concentration (probably through coiled-coil formation). Although this property may ultimately lead to protein aggregation, it is not sufficient to explain the temperature-dependent phase transitions observed. Indeed, increasing the ionic strength have no effect on phase separation, but rather promote aberrant aggregation by hydrophobic collapse^40^. Relaxation data also showed a stronger tendency of the PARMs to assemble into small oligomers, which could not be explained on the basis of the major α-helical conformation of the extended polyAla tract. In addition, significant α-helix to disordered conversions are observed coupled to phase transitions in expanded PHOX2B, in accordance with the decrease in thermal stability induced by PARMs^6^. Disruption of the α-helix indeed accelerated phase transitions. Disordered to α-helix conversions are established as early steps in amyloid aggregation^58^. However, the switch from α-helix to disordered conformations observed for PARMs coupled to phase transitions could represent the polymorphism that is expected to occur in the condensate interface^27,28^. Therefore, the remarkable disorder in addition to the reduced condensate fluidity indicates that PHOX2B phase transitions are triggered by phase separation coupled to networking or percolation transitions^25^. Considering that the polyAla-flanking stickers in PHOX2B display significant polymorphism due to proline *cis-trans* isomerization, enriched disorder in the polyAla tract would increase networking during phase transitions. Interestingly, there is a significant abundance of Gly and Pro residues flanking polyAla tracts in humans^59^, suggesting that the sticking mechanism proposed here could be general in PARMs. Therefore, our data indicate that the search of compounds that stabilize the major α-helical structures and deter PARM-promoted nascent conformations would represent a reasonable rationale for the search of therapeutics for CCHS and other polyAla disorders. In particular, our data imply that the C-terminal region of polyAla α-helix is significantly more dynamic, and could be the region to target the design of stabilizing compounds.

HSP40, HSP70 and HSP90 chaperones primarily identify nascent conformations and impede PHOX2B phase transitions, providing a direct link between conformational transitions and phase de-mixing. We recently identified a chaperone tendency to recognize short structured motifs in disordered clients^60^. Interestingly, here we show the selective chaperone arrest of minor disordered conformations in an otherwise structured segment of mutated PHOX2B. In particular, chaperones seize disordered conformations which could serve as secondary-structure templates priming fast phase transitions^28,61^. This selective identification is in agreement with the co-localization of HSP70 with the cytoplasmic aggregates formed by expanded PHOX2B, while it remained disperse in the cytosol in the presence of transfected wild-type PHOX2B^19^. In addition, up-regulation of HSP70-HSP40 machinery promotes the clearance of PARM aggregates^43,44,49^. Liquid-solid phase transitions are usually driven by the acquisition of β-sheet structure^29–32,55^. Remarkably, the minor β metamorphic population observed in expanded PHOX2B is consistent with ThT negative, α-helical PHOX2B aggregates^23^ which are adhered by minimal β-sheet steric zippers^7,45^. This peculiar sticking procedure would not be shared by other proteins undergoing phase separation and promoting ThT positive amyloid aggregates^26^. For instance, chaperones promote TDP-43 LLPS and further amyloid aggregation to protect the cell against the prion-like behavior of soluble TDP-43^62^, but inhibit PHOX2B phase transitions since it is the basis of its malfunction. Although we still do not fully understand the underlying mechanistic basis, realizing how chaperones display this variety of roles towards cellular protection is indeed fascinating.

## Methods

### Sample preparation

All the different human PHOX2B (UniProt KB Q99453, Origene) cDNA constructs were subcloned into modified pET28a vectors (Novagen) containing Thioredoxin (TXA) as a fusion protein followed by a six-histidine tag for Ni^2+^ affinity purification and a Tobacco Etch Virus (TEV) protease cleavage site. Construct nomenclature refers to the length of the contained sequence (XL, L, S and XS; being XL the full-length protein) followed by the number of alanines in the polyAla tract (20, 23 and 26). All cloning procedures were performed following the Gibson Assembly method (New England Biolabs). PolyAla expanded mutants were generated by mutagenic PCR. XS20 and XS26 were subsequently used as templates for the addition of a C-terminal cysteine residue (C315) for the covalent attachment of a fluorescent tag (detailed below). XS26 was used as template for the generation of the XS_GP_ variant (GenScript). Human HSP72, HSP90β (named HSP70 and HSP90, respectively, for simplicity) and DNAJB1 cDNA sequences were cloned into respective pET28a vectors (GenScript). All sequences were verified by DNA sequencing. Cloning procedures were performed with the following oligonucleotides in both DH5α and XL1 *E. coli* strains (Table 1).

**Table 1:** List of oligonucleotides used for cloning

	Forward	Reverse
XL20 insert	5’ATTTCCAGGGATCCATGTATAAAATGGAATATTC3’	5’GTGGTGCTCGAGTTAGAACATACTGCTC3’
XL20 vector	5´CTCGAGCACCACCACCACCACTG3´	5´GACATGGATCCCTGGAAATACAGGTTTTC3´
L20 insert	5’ATTTCCAGGGATCCATCCGCACCACCTTCAC3’	5’GTGGTGCTCGAGTTAGAACATACTGCTC3’
S20 insert	5’ATTTCCAGGGATCCGCTAAGTTTCGCAAGC3’	5’GTGGTGCTCGAGTTAGAACATACTGCTC3’
S20 vector	5´CTCGAGCACCACCACCACCACTG3´	5’CTTAGCGGATCCCTGGAAATACAGGTTTTC3’
XS20 insert	5’ATTTCCAGGGATCCGGCCCGGGGGGCCCGGG3’	5’GTGGTGCTCGAGTTAGAACATACTGCTC3’
XS20 vector	5´CTCGAGCACCACCACCACCACTG3´	5’GGGCCGGATCCCTGGAAATACAGGTTTTC3’
XS23	5’GCAAGGGCGGTGCAGCAGCAGCGGCTGCGGCTG3’	5’CAGCCGCAGCCGCTGCTGCTGCACCGCCCTTGC3’
XS26	5’GCAAGGGCGGTGCTGCAGCAGCAGCAGCAGCG3’	5’CGCTGCTGCTGCTGCTGCAGCACCGCCCTTGC3’
C315	5’GAGCAGTATGTTCTGTTAACTCGAGCACCACC3’	5´GGTGGTGCTCGAGTTAACAGAACATACTGCTC’3’

PHOX2B fragments (including WT, PARMs and XS_GP_ variant) were produced in BL21 star (DE3) *E. coli* strain. Cells were cultured in rich media until OD_600nm_ = 0.6. For ^15^N/^13^C isotopic labeling, cultures were then harvested by a 20-min centrifugation at 5,300 g and the pellets resuspended in ¼ of the initial volume in M9 medium supplemented with 4 g/l of ^13^C-glucose and 1 g/l of ^15^NH_4_Cl as the sole sources of C and N, respectively^63^. Cultures were incubated at 37 °C during 1–1.5 h for the adaption to the new media until the optical density increased one more unit than the initial value after resuspension. Protein expression (both in rich and minimal media) was induced by the addition of 0.5 mM (0.1 mM for L20 production) isopropyl β-d-1-thiogalactopyranoside (IPTG) for 16–20 h at 25 °C. Cells were harvested by centrifugation at 5,300 g during 25 minutes at 4 °C and resuspended in 30 ml/L of lysis buffer (50 mM KPi/500 mM NaCl/10 mM imidazole/2 mM β-mercaptoethanol [pH 8]), including 2 μl of protease inhibitors (ThermoFisher Scientific), 0.08 mg/ml of DNAse I (Sigma-Aldrich) and 1 mg/ml lysozyme (Sigma-Aldrich) and sonicated. Subsequently, a clarification step at 4000 g for 20 minutes at 4 °C in an Optima XPN-90 Ultracentrifuge (Beckman Coulter) was performed to remove the insoluble debris.

The clarified lysates were loaded onto pre-equilibrated Ni^2+^ affinity columns (Cytiva), adding 500 mM imidazole to the buffer for the elution (under the application of gradient elutions). TEV digestion was performed in 5 mM KPi/10 mM NaCl/10 mM imidazole/2 mM β-mercaptoethanol [pH 8], by an overnight incubation at 4 °C with 0.5–1 mg/ml of TEV protein (produced in house) per 15 mg of fusion protein. Cleaved PHOX2B protein constructs were directly cleared with a Ni^2+^ affinity purification as previously detailed, where PHOX2B was present in the flowthrough fractions. Samples were further purified by a serial anion and cation exchange chromatography using Hitrap Q HP and SP HP columns (Cytiva), respectively, using 5 mM KPi/10 mM NaCl/2 mM β-mercaptoethanol [pH 6.8]. Up to 1 M NaCl was added in gradient to the buffer for sample elution. Highly pure samples were concentrated below 0.06 mM using Vivaspin® Turbo 15 (3 MWCO) ultrafiltration tubes (Sartorius) in 5 mM KPi/10 mM NaCl/2 mM β-mercaptoethanol/0,03 % sodium azide [pH 6.8] (NMR buffer) and snap-frozen at −80 °C.

DNAJB1, HSP70 and HSP90 molecular chaperones were produced in BL21 (DE3) and Rosseta 2 (DE3) star *E. coli* strains, using 1 mM IPTG for induction at OD_600_ = 0.8–0.9 during 4 h at 37 °C. Cells were harvested and lysed in 20 mM Tris-HCl/500 mM NaCl/10 mM imidazole [pH 8] as explained previously. Recombinant proteins in the soluble fraction were purified by Ni^2+^ affinity chromatography using high density NiSO_4_ agarose beads (ABT), 20 mM Tris-HCl/500 mM NaCl/10 mM imidazole [pH 8] as binding buffer and binding buffer including 500 mM imidazole for elution. Proteins were further purified by size exclusion chromatography using HiLoad^TM^ 26/60 Superdex^TM^ 75 pg columns (Cytiva) in 10 mM Hepes/500 mM KCl/5 mM DTT [pH 7.5]. Fractions containing pure monomeric protein were pooled, concentrated using Vivaspin® 20 ultrafiltration tubes (10 MWCO, Sartorius) and stored at −80 °C. Chaperone and co-chaperone samples were buffer exchanged before the experiments into 20 mM Hepes/5 mM MgCl_2_/10 mM KCl/1 mM DTT [pH 6.8] using Zeba spin desalting columns (ThermoFisher).

XS20 and XS26 C315 mutants were tagged with ATTO-488 and ATTO-565 (ATTO-TEC GmbH) following the manufacturer protocol for fluorescent microscopy assays. After tagging, the excess of dye was removed with a size exclusion chromatography, using a Superdex ^TM^ 75 10/300 GL column (Cytiva). An average 45% tagging efficiency was confirmed spectrophotometrically.

### Turbidimetry assays

Protein samples of pure XS20, XS23, XS26 and L20 were prepared at 0.05, 0.125, 0.225 and 0.5 mM concentration in 5 mM KPi/10 mM NaCl/1 mM DTT/1 mM benzamidine/0,03% sodium azide [pH 6.8] (incubation buffer). XS_GP_ was measured at 0.165 mM. To monitor turbidity in presence of HSP70 and HSP90, 0.225 mM of PHOX2B proteins were incubated with 0.1 mM of the chaperones in 20 mM Hepes/10 mM NaCl/5 mM MgCl_2_/2 mM ATP/1 mM DTT/0,03% sodium azide [pH 6.8]. Turbidity was measured at 25 °C using Abs_600_ in a FLUOstar Omega (BMG LABTECH) multiwell plate reader using 96-well flat-bottom plates (Porvair Sciences). Measurements at 37 °C, including 10, 150 and 300 mM NaCl and 10% (w/v) PEG 4000 (Fisher Scientific) were performed using the same buffer and protein concentration as mentioned above. Samples were incubated for 84 h with 60 s of agitation at 100 rpms before each measurement point (10 minutes between measurement points). All data was technically and biologically replicated, and presented as the average between replicates with the standard deviation.

### Fluorescent microscopy

Fluorescent microscopy images were obtained in a Nikon Eclipse TE2000-U inverted microscope, a Nikon Ti2e-AXR-DUX-ST superresolution confocal microscopy, a Leica AF6000 LX and a Leica TCS SP8 STED confocal microscope. Protein samples were incubated in NMR buffer ranging from 0.1-0.25 mM protein concentration. Samples were first incubated at 37 °C for 30 seconds, placed for 3 minutes at 4 °C and finally incubated again at 37 °C for the corresponding period of time. Fluorescent labeled XS20 and XS26 (both labeled with ATTO-488 and ATTO-565) were mixed with unlabeled samples at a molar ratio 1:400-1:600 before exchange into NMR buffer. Colocalization experiments were performed using 0.1 mM (1:400) XS26-ATTO-488 and 0.15 mM (1:600) XS20-ATTO-565 incubated at 37 °C for 3.5 min. To study PHOX2B condensates in presence of chaperones, 0.1 mM PHOX2B proteins were incubated with 0.1 mM chaperones (1:1 ratio) in 20 mM Hepes/10 mM NaCl/5 mM MgCl_2_/2 mM ATP/1 mM DTT/0,03 % sodium azide [pH 6.8] buffer. Samples were spotted onto glass slides (ThermoFisher scientific) and imaged upright.

Confocal FRAP experiments were performed on a home-built confocal microscope based on an Olympus IX-71 inverted microscope. The ATTO-488 labeled samples were excited by a 532 nm pulsed laser (LDH-P-FA-530B, PicoQuant GmbH) at a repetition rate of 40 MHz. The laser is coupled into a single mode fiber (P3-488PM-FC, Thorlabs GmbH) to obtain a Gaussian beam profile. A linear polarizer (LPVISE100-A, Thorlabs GmbH) and a quarter-wave plate (AQWP05M- 600, Thorlabs GmbH) is used to obtain Circular polarized light. The light is focused onto the sample using an oil-immersion objective (UPLSAPO100XO, NA 1.40, Olympus Deutschland GmbH) and the position of the sample is adjusted using a piezo stage (P-517.3CD, Physik Instrumente (PI) GmbH & Co. KG) and controller (E-727.3CDA, Physik Instrumente GmbH & Co. KG). The emitted light is separated from the excitation beam by a dichroic beamsplitter (zt532/640rpc, Chroma) and focused onto a 50 μm diameter pinhole (Thorlabs GmbH). After the pinhole, the emission signal is directed by a dichroic beamsplitter (640 LPXR, Chroma) into a green (Brightline HC582/75, AHF; RazorEdge LP 532, Semrock) detection channel. Emission is focused onto avalanche photodiodes (SPCMAQRH- 14-TR, Excelitas) and the signals are registered by a time-correlated single photon counting (TCSPC) unit (HydraHarp400, PicoQuant). The setup is controlled by a commercial software package (SymPhoTime64, Picoquant GmbH). The surface was scan using a power of 1 µW (as measured at the entrance of the microscope body) to detect the condensates. To bleach part of the condensate, the confocal spot was focused nearby the condensate and the power increased to 175 µW for 2 seconds. For the preparation of the microscopy chamber, SecureSeal Hybridization chambers (2.6 mm depth, Grace Bio-Labs, USA) were glued on UV-Ozone cleaned (PSD-UV4, Novascan Technologies, USA) microscope coverslips. The surface was further cleaned with 1 M KOH for 15 min. After that, the chambers were washed three times with water and three times with PBS buffer. The surface was finally passivated with BSA-biotin for at least 1 h. Right before the experiments, the BSA was removed and the chamber washed three times with NMR buffer. Protein samples were incubated as mentioned below.

TIRF-based FRAP measurements and time evolution observation of the condensates were performed on a commercial Nanoimager S microscope (ONI Ltd., UK) under TIRF illumination. Blue excitation was applied at 488 nm to excite the ATTO-488 labeled proteins. The auto focus of the setup was used to maintain the imaged samples on focus. Protein sample concentration, mixtures and incubation protocols were as mentioned above. Incubated samples were diluted 1/30 with NMR buffer in the microscopy chamber. Measurements were performed at 30 °C. In order to standardize the measurements, data acquisition was started 6 min after the last 37 °C incubation step. To study the time evolution of the condensates, observations (two frame video with 100 ms integration time per frame and 0.2 mW blue excitation) were performed at 6, 12, 18, 24 and 30 min. In each observation, 25 field of views were acquired in a rectangular pattern (5 × 5) covering 117 µm × 117 µm. Duplicates were done for all the conditions tested, and averaged data is presented including standard deviation. To avoid being biased by diffraction limit, only condensates equal or larger than 2 pixels (each pixel being 117 nm) were included in the analysis. The following light program was set for FRAP measurements: i) Pre-bleaching observation (two frame video with 100 ms integration time per frame and 0.2 mW of blue excitation). ii) Bleaching (2.5 s of blue excitation at 169 mW power). iii) After-bleaching observation (two frame video with 100 ms integration time per frame and 0.2 mW blue excitation). iv) 360 s of recovery step without light excitation. v) Recovery after photobleaching observation (two frame video with 100 ms integration time per frame and 0.2 mW blue excitation).

TIRF videos were analyzed using ImajeJ^64^. Only the first frame of the video was analyzed to avoid significant photobleaching of the field of view during the observation. The background of the images was subtracted using a rolling ball of 50 pixels radius. Condensates were analyzed using the Analyze Particles routine, which automatically identifies the condensates and calculates their intensity and size. For the quantification, the images were binarised (16-bit) and thresholded using the Otsu algorithm^64^. Only properly focused condensates were included in the analyses, which contained information on particle area and fluorescence intensity.

### NMR assignments, ^15 ^N relaxation and structure calculation

All the NMR spectra were acquired in an 800 MHz (^1^H) Bruker AVNEO spectrometer equipped with a Z-gradient cryoprobe. ^1^H chemical shifts were referenced to the internal reference sodium trimethylsilylpropanesulfonate (DSS), and ^15^N and ^13^C chemical shifts were referenced indirectly to ^1^H using the corresponding gyromagnetic ratios^65^. NMR assignments for XS20 were obtained at 5 °C and 25 °C using 0.5 mM of ^15^N/^13^C-labeled XS20 in NMR buffer. The following NMR spectra were acquired to obtain unambiguous assignments at both temperatures: 2D ^15^N- and ^13^C-HSQCs, 2D CON, 3D HNCO, HN(CA)CO, HNCA, CBCA(CO)NH, hCC(CO)NH (15 ms mixing time) and HBHA(CO)NH. Sequential connectivities were obtained by high-resolution 3D HNNH spectra (using >90 points in F1 dimension), which enabled the unambiguous assignment of the polyAla segments even for the highly overlapped regions of the spectra. The C-terminal residues from the XS constructions (namely residues 307-314) were not assigned in the spectra. HNHA and ^15^N-NOESY-HSQC and ^13^C-NOESY-HSQC (using 100 and 120 ms mixing time, respectively) were used to obtain J-couplings and NOEs for structure calculation. Assignments for XS20 at both temperatures were deposited in the BMRB database (codes 51978 and 51979, respectively). Additional XS20 samples were incubated up to 14 days at 25 °C with no agitation in incubation buffer before the NMR experiments. Only minor sample degradation within NMR incubation times was detected by mass spectrometry and gel electrophoresis. Assignment of 0.5 mM ^15^N/^13^C-labeled XS23 and XS26 in NMR buffer were obtained at 25 °C by means of 2D ^15^N- and ^13^C-HSQCs, 2D CON, 3D HNCO, HN(CA)CO, HNCA, CBCA(CO)NH and HNNH spectra. Additional samples of XS23 and XS26 were incubated for 14 and 7 days at 25 °C in incubation buffer with no agitation, respectively. Sequential connectivities were lost for the new crosspeaks appearing in the ^15^N-HSQC XS23 and XS26 spectra upon incubation. However, 3D CBCA(CO)NH spectra revealed that the large majority of those peaks belonged to residues preceded by Ala residues in disordered conformation, due to their characteristic Cα and Cβ chemical shifts^38^. Therefore, the new crosspeaks in the ^15^N-HSQC were assigned following arbitrary numbering, which was maintained in the nascent crosspeaks detected in ^13^C-HSQC and CACO spectra. In addition, alanine residues from the central section of the polyAla α-helix (residues 253-258 in particular) showed strong signal overlap in the H-N correlations, which enabled to observe nascent crosspeaks in the 3D CBCA(CO)NH spectra with chemical shifts corresponding to alanines in alternative conformations. These disordered polyAla crosspeaks were also observed in 2D ^13^C-HSQC spectra, which displays the whole palette of conformation-dependent C-H correlations. The nascent alanine moieties were reproduced in different protein batches upon incubation. 0.5 mM ^15^N/^13^C-labeled L20 was used to validate the structure of XS20 using 2D ^15^N- and ^13^C-HSQCs, 2D CON, 3D HNCO and CBCA(CO)NH spectra at 5 °C. 0.5 mM ^15^N/^13^C-labeled XS_GP_ was assigned at 25 °C by means of 2D ^15^N- and ^13^C-HSQCs, 2D CON, 3D CBCA(CO)NH and HNNH spectra.

Loss of NMR signal intensity was calculated comparing the ^15^N-HSQC NMR signal intensities for freshly prepared XS20 samples at 0.5 mM and 25 °C *vs*. the NMR signal intensity for XS20 samples which were incubated at 25 °C. For CACO spectra, signal intensity for 0.225 mM XS26 incubated in incubation buffer for 48 h was compared to that for fresh XS26. Secondary chemical shifts were calculated as ΔCα-ΔCβ, where ΔCα and ΔCβ are the differences between experimentally obtained Cα and Cβ chemical shifts at the specific protein concentrations and computed shifts for disordered PHOX2B constructs at the same temperature and pH (https://spin.niddk.nih.gov/bax/nmrserver/Poulsen_rc_CS/)^66,67^. ^3^J_HNHA_ coupling constants were calculated on the basis of 3D HNHA experiments^68^. ^15^N longitudinal (*R*_1_), rotating frame (*R*_1ρ_) and (^1^H)-^15^N NOE relaxation data for 0.05 and 0.5 mM XS20, 0.5 mM XS23 and 0.5 mM XS26 samples in NMR buffer were obtained using standard Bruker pulse sequences acquired at 25 °C (or 5 °C and 25 °C for XS20) in the 800 MHz spectrometer. In all relaxation experiments the spectral width was 12500 Hz for ^1^H and 2027 Hz for ^15^N dimensions. Eight relaxation delays (20; 60; 100; 240; 600; 1000; 1600 and 2400 ms) were used for ^15^N *R*_1_ measurements, as well as to measure ^15^N *R*_1ρ_ values (using 8.00; 35.99; 75.99; 100.00; 156.00; 200.00; 347.99 and 699.99 ms). Relaxation values and uncertainties were calculated by fitting an exponential decay to the data. Het-NOEs were calculated from the ratio of cross peak intensities in spectra collected with and without amide proton saturation during the recycle delay. Uncertainties in peak heights were determined from the standard deviation of the intensity distribution in signal-less spectral regions. All NMR spectra were processed in Topspin 4.1.1 and analyzed in Sparky 3.19^69^.

The S^2^ order parameter was derived from the chemical shifts obtained by TALOS-N^70^. The overall correlation time (τ_c_) was estimated from the ratios of the mean values of *T*_1_ and *T*_2_ as τ_c_ ≃1/(4 *π* ^∗ 15^N frequency in Hz) ^∗^ ((6^∗^ *R*_2_/*R*_1_) – 7))^½^ which was derived from eqn. 8 of Kay et al. ^37^ that considers J(0) and J(ω) spectral densities and discounts terms of higher frequencies from a subset of residues with little internal motion and no significant exchange broadening. This subset excluded residues with *T*_2_ values lower than the average minus one standard deviation, unless their corresponding *T*_1_ values were larger than the average plus one standard deviation^71^. Experimental τ_c_ values for XS20 at 5 °C and 25 °C were compared to the correlation time calculated on the basis of its size and shape in solution, using HydroNMR^72^. The atomic structures for the fragment 241–260 of PHOX2B at both temperatures (pure α-helix) were considered in the calculations, and τ_c_ values were averaged for the ensemble structures. Temperature-dependent solvent viscosity was considered in the calculations, applying 3.0 Å of atomic element radius. 3D coordinates of canonical α-helices for 20, 23 and 26 alanines were also generated using the builder module in PyMOL (PyMOL Molecular Graphics System, Version 1.8.x Schrödinger, LLC.). HydroNMR was applied to the generated α-helices, which reported the following τ_c_ averaged values (in ns): 1.85 (XS20 at 5 °C), 1.05 (XS20 at 25 °C), 1.24 (XS23 at 25 °C) and 1.43 (XS26 at 25 °C). These values indicate an increase of 0.19 ns in τ_c_ every 3 additional alanines, which is significantly smaller than the experimental values obtained for the extended variants. These discrepancies, in addition to the difference between experimental and calculated τ_c_ for XS20 structure, indicate that PHOX2B has a tendency to assemble in solution through the polyAla helix, and that PARMs increase these associations. Relaxation and correlation time values are shown in Supplementary Table 1.

The NMR structure of XS20 at 5 °C and 25 °C was calculated with the program CYANA v3.98.13^73^ based on experimental NOE-derived distance constraints and TalosN-derived dihedral constraints^36^ following the standard 7-cycle iterative process and a final annealing using the list of restraints obtained in the last cycle. One hundred structures were generated using the mentioned procedure. Structural statistics for the calculated structures are summarized in Supplementary Tables 2 and 3. α-helical boundaries were experimentally validated by the observation of *i* + 3 and *i* + 4 H-H NOEs. The 20 conformers with the lowest target function values were selected and deposited in the Protein Data Bank under the accession numbers 8PTL and 8PUI. The structural ensembles were visualized and examined using MolMol^74^ and Pymol v2.0 (PyMOL Molecular Graphics System, Version 2.0 Schrödinger, LLC.). PROCHECK-NMR^75^ version 3.4.4 was used to analyze the quality of the refined structures. Ramachandran statistics showed 77.5% most favored regions and 22.5% for the additionally allowed favored regions for the structure at 5 °C, while the statistics were 76.3% of most favored regions and 23.7% of additionally allowed favored regions for the structure at 25 °C.

NMR titrations were based on 2D ^15^N-HSQC, ^13^C-HSQC and CACO spectra measured at 25 °C in the 800 MHz spectrometer in 20 mM Hepes/10 mM NaCl/5 mM MgCl_2_/2 mM ATP/1 mM DTT/0,03 % sodium azide [pH 6.8] buffer. 0.07 mM (for the ^15^N-HSQC spectra), 0.1 mM (for the ^13^C-HSQC spectra) and 0.225 mM (for the CACO spectra) ^15^N/^13^C-labeled XS26 were mixed with fresh 0.14 mM unlabeled (therefore, NMR invisible) HSP70 and HSP90 (for the ^15^N-HSQC-based titrations) or 0.1 mM fresh HSP70 (for the ^13^C-HSQC-based titrations), while CACO samples were incubated 48 h with no agitation at 25 °C with 0.45 mM HSP70. CACO-based titrations with the co-chaperone DNAJB1 were done mixing 0.14 mM of ^15^N/^13^C XS26, 0.28 mM of unlabeled HSP70 and 0.14 mM of unlabeled DNAJB1 in the abovementioned buffer and incubating for 48 h with no agitation at 25 °C. The data shown are the signal intensity decay for the interactions, which is obtained comparing the normalized NMR signal intensity for the complex ratios and the intensity for XS26 alone at the same concentration. For the ^13^C-HSQC-based titrations, values shown are averaged between the Cα and Cβ crosspeak intensities, and include alternative conformations both for polyAla and nascent alanine moieties.

Diffusion experiments were acquired at 25 °C in NMR buffer in the 800 MHz spectrometer using a pseudo 2D ^1^H NMR pulse sequence using stimulated echo with bipolar gradient pulses. Diffusion time (160 ms) and length of DOSY gradient pulse (1.4 ms) were optimized to obtain a correct signal decay using an exponential gradient ramp. Both XS20 and XS26 were measured at 0.05, 0.1 and 0.5 mM at different incubation times (7 days at 25 °C). Gradients were calibrated before measuring DOSY experiments using a “doped water” sample (1 % H_2_O + 0.1 mg GdCl_3_/ml D_2_O + 0.1 % ^13^CH_3_OH) following the Bruker protocol. The pseudo 2D DOSY dataset was processed in Topspin 4.3.0 and analyzed with Dynamics Center (Bruker). Diffusion coefficient in 8 M urea was corrected applying a viscosity coefficient of 1.663 compared to water^76^.

### Circular Dichroism (CD)

CD spectra were recorded at 25 °C before and after thermal denaturation at 95 °C in a J-810 Jasco polarimeter using a 0.1 cm cuvette and 0.265 mg/ml (for XS20 and XS26) and 0.132 mg/ml (for XS23) protein concentration. The sample buffer was 5 mM KPi/10 mM NaCl /1 mM DTT/0,01% azide [pH 6.8]. Samples were serially diluted in water to minimize signal intensity. Thermal unfolding was induced by heating the samples at 95 °C for 15 min followed by a 6 min cool down to 25 °C. For each measurement, ten scans were averaged and, after correction for the buffer contribution, transformed into mean residue weight ellipticity (Θ · 10^−3^ (degree · cm^2^ · dmol^−1^)) as follows (Eq. (1)):

1 $${Molar}\,{Ellipticity}=\frac{{{{{{\rm{\theta }}}}}}/10x\,{Optical}\,{path}\,{lenght} \, \left({cm}\right) \, x\,{Concentration} \, \left(M\right)}{{residue}\,{number}-1}$$

### Reporting summary

Further information on research design is available in the Nature Portfolio Reporting Summary linked to this article.

### Supplementary information

Supplementary Information

Peer Review File

Reporting Summary

### Source data

Source Data

## Data Availability

All materials are readily available from the corresponding author upon request. Source data are provided with this paper. The structural data generated in this study have been deposited in the PDB database under the accession codes 8PTL and 8PUI and the chemical shifts data in the BMRB database under the accession codes 51978 and 51979. Source data are provided as a Source Data file. Source data are provided with this paper.

## References

[1] Bernacki JP, Murphy RM (2011). Length-dependent aggregation of uninterrupted polyalanine peptides. Biochemistry.

[2] Chavali S, Singh AK, Santhanam B, Babu MM (2020). Amino acid homorepeats in proteins. Nat. Rev. Chem..

[3] Matera I (2004). PHOX2B mutations and polyalanine expansions correlate with the severity of the respiratory phenotype and associated symptoms in both congenital and late onset Central Hypoventilation syndrome. J. Med. Genet..

[4] Cummings CJ, Zoghbi HY (2000). Fourteen and counting: unraveling trinucleotide repeat diseases. Hum. Mol. Genet..

[5] Messaed C, Rouleau GA (2009). Molecular mechanisms underlying polyalanine diseases. Neurobiol. Dis..

[6] Pirone L (2019). Molecular insights into the role of the polyalanine region in mediating PHOX2B aggregation. FEBS J..

[7] Pelassa I (2014). Association of polyalanine and polyglutamine coiled coils mediates expansion disease-related protein aggregation and dysfunction. Hum. Mol. Genet..

[8] Baias M (2017). Structure and Dynamics of the Huntingtin Exon-1 N-Terminus: A Solution NMR Perspective. J. Am. Chem. Soc..

[9] Escobedo A (2019). Side chain to main chain hydrogen bonds stabilize a polyglutamine helix in a transcription factor. Nat. Commun..

[10] Elena-Real CA (2023). The structure of pathogenic huntingtin exon 1 defines the bases of its aggregation propensity. Nat. Struct. Mol. Biol..

[11] Coleman M, Boros SJ, Huseby TL, Brennom WS (1980). Congenital central hypoventilation syndrome. A report of successful experience with bilateral diaphragmatic pacing. Arch. Dis. Child.

[12] Di Lascio S, Benfante R, Cardani S, Fornasari D (2020). Research Advances on Therapeutic Approaches to Congenital Central Hypoventilation Syndrome (CCHS). Front. Neurosci..

[13] Amiel J (2003). Polyalanine expansion and frameshift mutations of the paired-like homeobox gene PHOX2B in congenital central hypoventilation syndrome. Nat. Genet..

[14] Di Lascio S (2018). Structural and functional differences in PHOX2B frameshift mutations underlie isolated or syndromic congenital central hypoventilation syndrome. Hum. Mutat..

[15] Brunet JF, Pattyn A (2002). Phox2 genes - from patterning to connectivity. Curr. Opin. Genet. Dev..

[16] Sasaki A (2003). Molecular analysis of congenital central hypoventilation syndrome. Hum. Genet..

[17] Patwari PP (2010). Congenital central hypoventilation syndrome and the PHOX2B gene: a model of respiratory and autonomic dysregulation. Respir. Physiol. Neurobiol..

[18] Bachetti T (2005). Distinct pathogenetic mechanisms for PHOX2B associated polyalanine expansions and frameshift mutations in congenital central hypoventilation syndrome. Hum. Mol. Genet..

[19] Trochet D (2005). Molecular consequences of PHOX2B missense, frameshift and alanine expansion mutations leading to autonomic dysfunction. Hum. Mol. Genet.

[20] Di Zanni E (2012). In vitro drug treatments reduce the deleterious effects of aggregates containing polyAla expanded PHOX2B proteins. Neurobiol. Dis..

[21] Di Lascio S (2013). Transcriptional dysregulation and impairment of PHOX2B auto-regulatory mechanism induced by polyalanine expansion mutations associated with congenital central hypoventilation syndrome. Neurobiol. Dis..

[22] Dubreuil V (2008). A human mutation in Phox2b causes lack of CO2 chemosensitivity, fatal central apnea, and specific loss of parafacial neurons. Proc. Natl Acad. Sci. USA.

[23] Polling S (2015). Polyalanine expansions drive a shift into α-helical clusters without amyloid-fibril formation. Nat. Struct. Mol. Biol..

[24] Basu S (2020). Unblending of Transcriptional Condensates in Human Repeat Expansion Disease. Cell.

[25] Mittag T, Pappu RV (2022). A conceptual framework for understanding phase separation and addressing open questions and challenges. Mol. Cell.

[26] Carrasco J (2023). Metamorphism in TDP-43 prion-like domain determines chaperone recognition. Nat. Commun..

[27] Ma B, Nussinov R (2002). Molecular dynamics simulations of alanine rich beta-sheet oligomers: Insight into amyloid formation. Protein Sci..

[28] Farag M (2022). Condensates formed by prion-like low-complexity domains have small-world network structures and interfaces defined by expanded conformations. Nat. Commun..

[29] Murray DT (2017). Structure of FUS Protein Fibrils and Its Relevance to Self-Assembly and Phase Separation of Low-Complexity Domains. Cell.

[30] Hughes MP (2018). Atomic structures of low-complexity protein segments reveal kinked β sheets that assemble networks. Science.

[31] Luo F (2018). Atomic structures of FUS LC domain segments reveal bases for reversible amyloid fibril formation. Nat. Struct. Mol. Biol..

[32] Gui X (2019). Structural basis for reversible amyloids of hnRNPA1 elucidates their role in stress granule assembly. Nat. Commun..

[33] Burke KA, Janke AM, Rhine CL, Fawzi NL (2015). Residue-by-Residue View of In Vitro FUS Granules that Bind the C-Terminal Domain of RNA Polymerase II. Mol. Cell.

[34] Brady JP (2017). Structural and hydrodynamic properties of an intrinsically disordered region of a germ cell-specific protein on phase separation. Proc. Natl Acad. Sci. USA.

[35] Galvanetto N (2023). Extreme dynamics in a biomolecular condensate. Nature.

[36] Shen Y, Bax A (2013). Protein backbone and sidechain torsion angles predicted from NMR chemical shifts using artificial neural networks. J. Biomol. NMR.

[37] Kay LE, Torchia DA, Bax A (1989). Backbone dynamics of proteins as studied by 15N inverse detected heteronuclear NMR spectroscopy: application to staphylococcal nuclease. Biochemistry.

[38] Wang Y, Jardetzky O (2002). Probability-based protein secondary structure identification using combined NMR chemical-shift data. Protein Sci..

[39] Alberti S, Gladfelter A, Mittag T (2019). Considerations and Challenges in Studying Liquid-Liquid Phase Separation and Biomolecular Condensates. Cell.

[40] van Dijk E, Hoogeveen A, Abeln S (2015). The hydrophobic temperature dependence of amino acids directly calculated from protein structures. PLoS Comput. Biol..

[41] Ciani B, Hutchinson EG, Sessions RB, Woolfson DN (2002). A designed system for assessing how sequence affects alpha to beta conformational transitions in proteins. J. Biol. Chem..

[42] Shinchuk LM (2005). Poly-(L-alanine) expansions form core beta-sheets that nucleate amyloid assembly. Proteins.

[43] Albrecht AN (2004). A molecular pathogenesis for transcription factor associated poly-alanine tract expansions. Hum. Mol. Genet..

[44] Bachetti T (2007). Geldanamycin promotes nuclear localisation and clearance of PHOX2B misfolded proteins containing polyalanine expansions. Int J. Biochem. Cell Biol..

[45] Lilliu E (2018). Polyserine repeats promote coiled coil-mediated fibril formation and length-dependent protein aggregation. J. Struct. Biol..

[46] Gojobori J, Ueda S (2011). Elevated evolutionary rate in genes with homopolymeric amino acid repeats constituting nondisordered structure. Mol. Biol. Evol..

[47] Orr HT, Zoghbi HY (2007). Trinucleotide repeat disorders. Annu. Rev. Neurosci..

[48] Caburet S (2004). A recurrent polyalanine expansion in the transcription factor FOXL2 induces extensive nuclear and cytoplasmic protein aggregation. J. Med. Genet..

[49] Utsch B (2007). Molecular characterization of HOXA13 polyalanine expansion proteins in hand-foot-genital syndrome. Am. J. Med. Genet. A.

[50] Iizuka Y (2021). Toxicity of internalized polyalanine to cells depends on aggregation. Sci. Rep..

[51] Mensah MA (2023). Aberrant phase separation and nucleolar dysfunction in rare genetic diseases. Nature.

[52] Lester E (2023). Cytosolic condensates rich in polyserine define subcellular sites of tau aggregation. Proc. Natl Acad. Sci. USA.

[53] Bravo-Arredondo JM (2018). The folding equilibrium of huntingtin exon 1 monomer depends on its polyglutamine tract. J. Biol. Chem..

[54] Milner-White JE, Watson JD, Qi G, Hayward S (2006). Amyloid formation may involve alpha- to beta sheet interconversion via peptide plane flipping. Structure.

[55] Lu J (2020). CryoEM structure of the low-complexity domain of hnRNPA2 and its conversion to pathogenic amyloid. Nat. Commun..

[56] Blondelle SE, Forood B, Houghten RA, Pérez-Payá E (1997). Polyalanine-based peptides as models for self-associated beta-pleated-sheet complexes. Biochemistry.

[57] Zhang H (2015). RNA Controls PolyQ Protein Phase Transitions. Mol. Cell.

[58] Abedini A, Raleigh DP (2009). A role for helical intermediates in amyloid formation by natively unfolded polypeptides?. Phys. Biol..

[59] Mier P, Elena-Real CA, Cortés J, Bernadó P, Andrade-Navarro MA (2022). The sequence context in poly-alanine regions: structure, function and conservation. Bioinformatics.

[60] Hervás R, Oroz J (2020). Mechanistic Insights into the Role of Molecular Chaperones in Protein Misfolding Diseases: From Molecular Recognition to Amyloid Disassembly. Int J. Mol. Sci..

[61] Kameda T, Takada S (2006). Secondary structure provides a template for the folding of nearby polypeptides. Proc. Natl Acad. Sci. USA.

[62] Bolognesi B (2019). The mutational landscape of a prion-like domain. Nat. Commun..

[63] Marley J, Lu M, Bracken C (2001). A method for efficient isotopic labeling of recombinant proteins. J. Biomol. NMR.

[64] Schneider CA, Rasband WS, Eliceiri KW (2012). NIH Image to ImageJ: 25 years of image analysis. Nat. Methods.

[65] Markley JL (1998). Recommendations for the presentation of NMR structures of proteins and nucleic acids. IUPAC-IUBMB-IUPAB Inter-Union Task Group on the Standardization of Data Bases of Protein and Nucleic Acid Structures Determined by NMR Spectroscopy. J. Biomol. NMR.

[66] Kjaergaard M, Poulsen FM (2011). Sequence correction of random coil chemical shifts: correlation between neighbor correction factors and changes in the Ramachandran distribution. J. Biomol. NMR.

[67] Kjaergaard M, Brander S, Poulsen FM (2011). Random coil chemical shift for intrinsically disordered proteins: effects of temperature and pH. J. Biomol. NMR.

[68] Vuister GW, Delaglio F, Bax A (1993). The use of 1JC alpha H alpha coupling constants as a probe for protein backbone conformation. J. Biomol. NMR.

[69] Lee W, Tonelli M, Markley JL (2015). NMRFAM-SPARKY: enhanced software for biomolecular NMR spectroscopy. Bioinformatics.

[70] Wang IF (2012). The self-interaction of native TDP-43 C terminus inhibits its degradation and contributes to early proteinopathies. Nat. Commun..

[71] Pawley NH, Wang C, Koide S, Nicholson LK (2001). An improved method for distinguishing between anisotropic tumbling and chemical exchange in analysis of 15N relaxation parameters. J. Biomol. NMR.

[72] García de la Torre J, Huertas ML, Carrasco B (2000). HYDRONMR: prediction of NMR relaxation of globular proteins from atomic-level structures and hydrodynamic calculations. J. Magn. Reson.

[73] Güntert P (2004). Automated NMR structure calculation with CYANA. Methods Mol. Biol..

[74] Koradi R, Billeter M, Wüthrich K (1996). MOLMOL: a program for display and analysis of macromolecular structures. J. Mol. Graph..

[75] Laskowski RA, Rullmannn JA, MacArthur MW, Kaptein R, Thornton JM (1996). AQUA and PROCHECK-NMR: programs for checking the quality of protein structures solved by NMR. J. Biomol. NMR.

[76] Kawahara K, Tanford C (1966). Viscosity and density of aqueous solutions of urea and guanidine hydrochloride. J. Biol. Chem..

