# Supplementary material for: Alternative low-populated conformations prompt phase transitions in polyalanine repeat expansions
Source: Nat Commun. 2024 Mar 2;15:1925. doi: 10.1038/s41467-024-46236-5 (PMC10908835; doi:10.1038/s41467-024-46236-5)
Supplement: Supplementary file 1 — Supplementary Information [file 41467_2024_46236_MOESM1_ESM.pdf]

Alternative low-populated conformations prompt phase transitions in polyalanine repeat expansions

Rosa Antón, Miguel Á. Treviño, David Pantoja-Uceda, Sara Félix, María Babu, Eurico J. Cabrita, Markus Zweckstetter, Philip Tinnefeld, Andrés M. Vera, Javier Oroz

Supplementary Figures

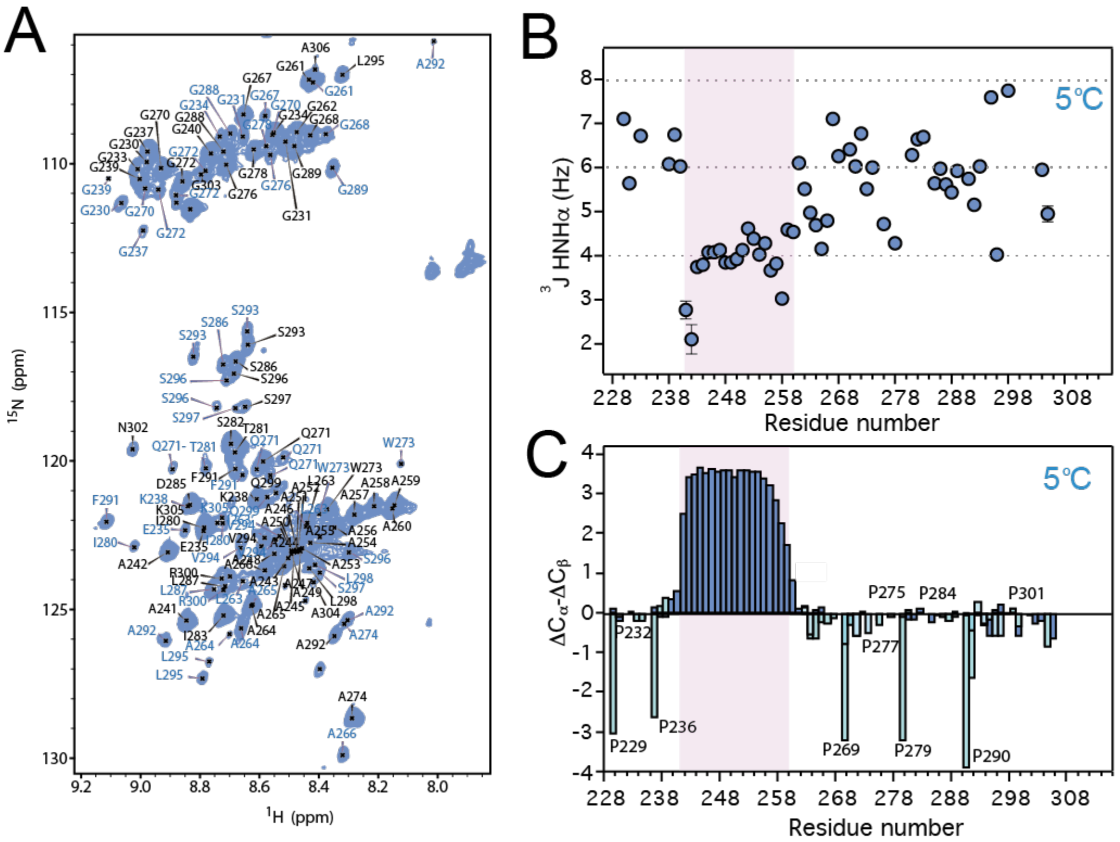

Supplementary Figure 1. XS20 assigned NMR spectra, J-couplings and alternative isomers. A) Assigned ^{15}N-HSQC spectrum of XS20 (0.5 mM) at 5 °C. Alternative conformations are labeled in blue. **B)** J-coupling assessment of the secondary structure propensities for XS20 at 5 °C. Values around 4 Hz indicate α-helix formation, while values around 8 Hz are typical of β-strand structures. Errors are calculated based on NMR S/N ratio. **C)** Secondary chemical shifts for XS20 at 5 °C. In these plots, positive values are representative of α-helical conformations, while negative values correspond to β-strands. Prolines in *cis* isomer conformations (negative bars in the plot, light blue) amount to *circa* 15% and are responsible for the alternative conformations observed in the NMR spectra. The purple shadows in the plots in (**B**) and (**C**) limit the polyAla region. Source data are provided as a Source Data file.

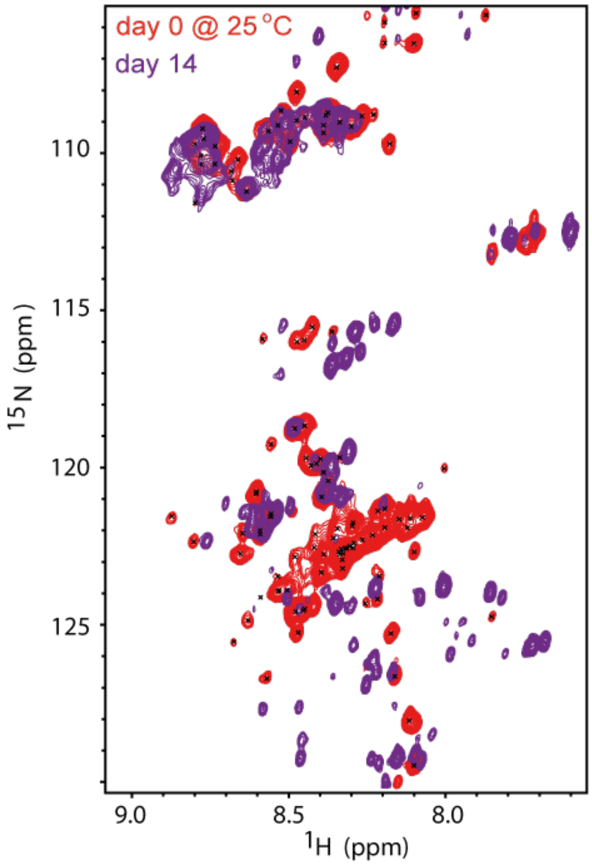

Supplementary Figure 2. ^{15}N HSQC XS20 spectral changes upon long incubation. XS20 ^{15}N-HSQC spectra at day 0 (red) and 14 (purple) of incubation at 25 °C. The polyAla tract appears at 120-125 (^{15}N) and 8.1-8.4 ppm (^{1}H). Protein concentration was 0.5 mM.

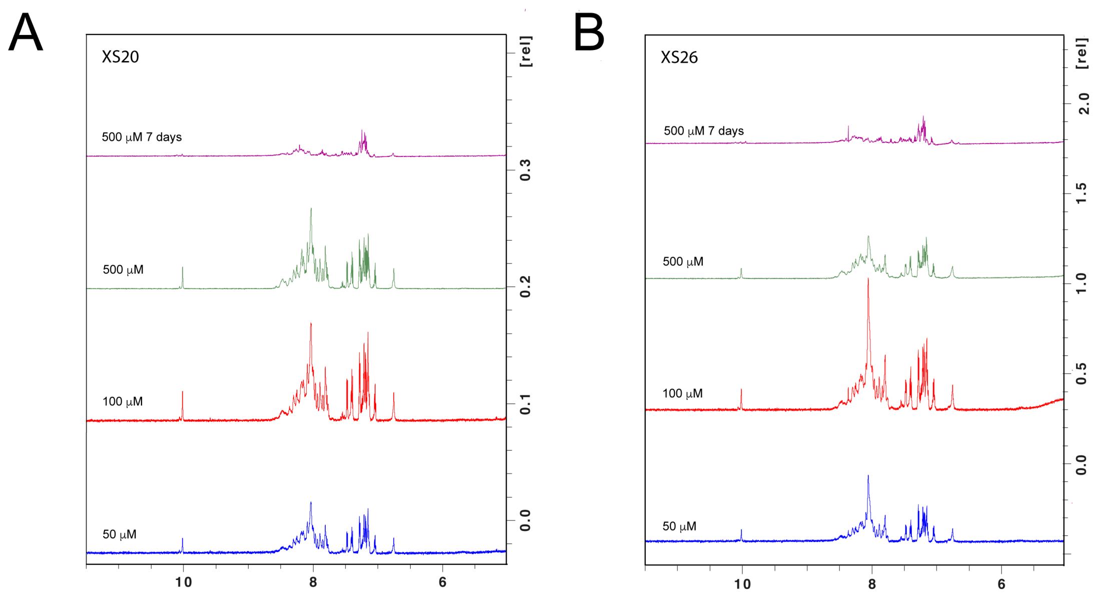

C

	D (m^2s^{-1})	
	0 days	7 days
XS20 50 µM	1,324 E-10	
XS20 100 µM	1,478 E-10	
XS20 500 µM	1,409 E-10	1,81 E-10

D

	D (m^2s^{-1})	
	0 days	7 days
XS26 50 µM	1,432 E-10	
XS26 100 µM	1,370 E-10	
XS26 500 µM	1,451 E-10	2,22 E-10
XS26 100 µM Urea	1,577 E-10	

Supplementary Figure 3. Concentration and incubation dependence of XS20 and XS26 NMR signals and diffusion coefficients. A) 1D NMR spectra for XS20 at 0.05 (blue), 0.1 (red), 0.5 mM (green) and 0.5 mM after 7 days of incubation at 25 °C (magenta). While signal intensity at 0.1 mM increases relative to 0.05 mM, the signal intensity at 0.5 mM decreases, indicating oligomerization. Upon incubation at high protein concentration, the signal intensity significantly decreases due to large oligomer assembly or aggregation. **B)** 1D NMR spectra for XS26 at 0.05 (blue), 0.1 (red), 0.5 mM (green) and 0.5 mM after 7 days of incubation at 25 °C (magenta). The signal intensity decay for XS26 at 0.5 mM (green) is more evident than for XS20 at 0.5 mM (**A**), in agreement with XS26 stronger assembly propensities. **C, D)** Diffusion coefficients for XS20 (**C**) and XS26 (**D**) at 25 °C at the mentioned protein concentrations and incubation time. Values indicate that the observable NMR signals arise from similar assembly species in all conditions tested. Diffusion coefficient in 8 M urea was corrected for the higher solvent viscosity. The sharp peak around 8 ppm in (**A, B**) correspond to the polyAla α-helical tract. No additional sharp peaks appear after incubation, indicating lack of protein degradation. Spectra contained identical magnification.

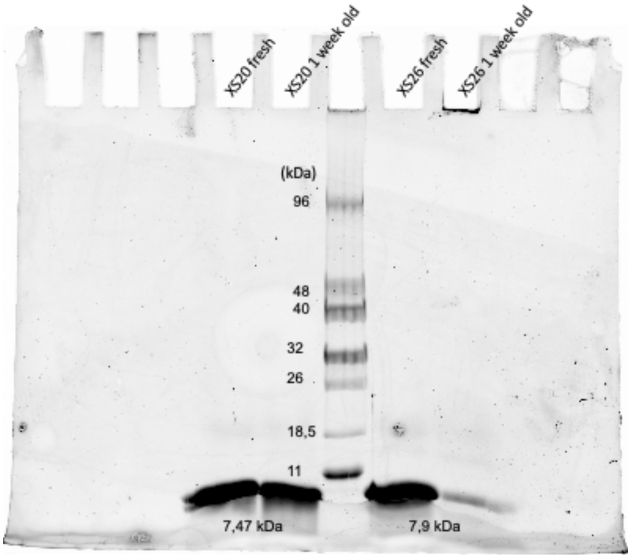

Supplementary Figure 4. SDS-PAGE of incubated samples shows no significant protein degradation. 0.1 mM XS20 and XS26 incubated for 7 days at 25 °C show no apparent degradation. Upon aggregation, a significant portion of incubated XS26 remains in the casted well unable to penetrate in the gel. Source data are provided as a Source Data file.

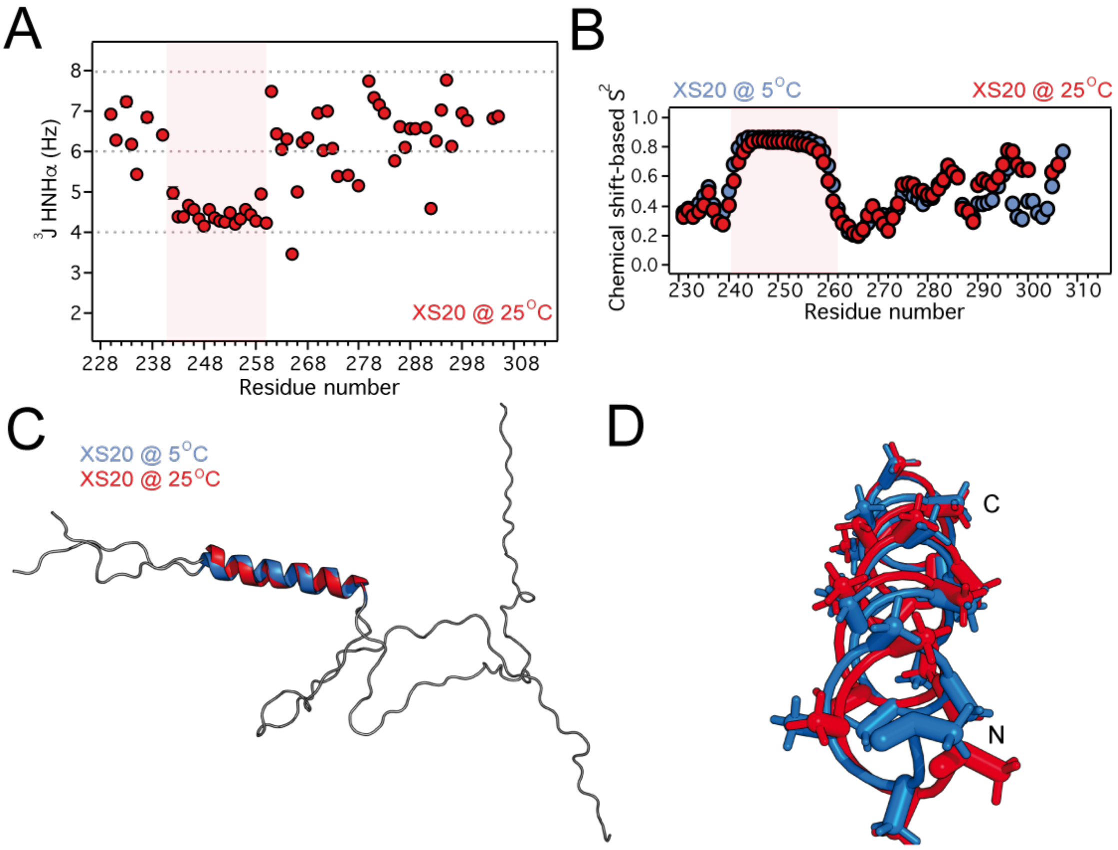

Supplementary Figure 5. XS20 J-couplings and S^2 order parameters at 25 °C. A) J-coupling assessment of the secondary structure propensities of XS20 at 25 °C. **B)** Chemical shift-based S^2 order parameters calculated by TALOS-N[1] for XS20 at 5 °C (blue) and 25 °C (red). **C)** Structural alignment of the lowest energy conformer of XS20 at 5 °C (blue) and 25 °C (red). **D)** Detail of the superimposed α-helices of the polyAla tract from PHOX2B at 5 °C (blue) and 25 °C (red) showing that the sidechain disposition is barely identical. N- and C-terminus are indicated. The red shadows in the plots in (**A**) and (**B**) limit the α-helix adopted by XS20 at 25 °C. Source data are provided as a Source Data file.

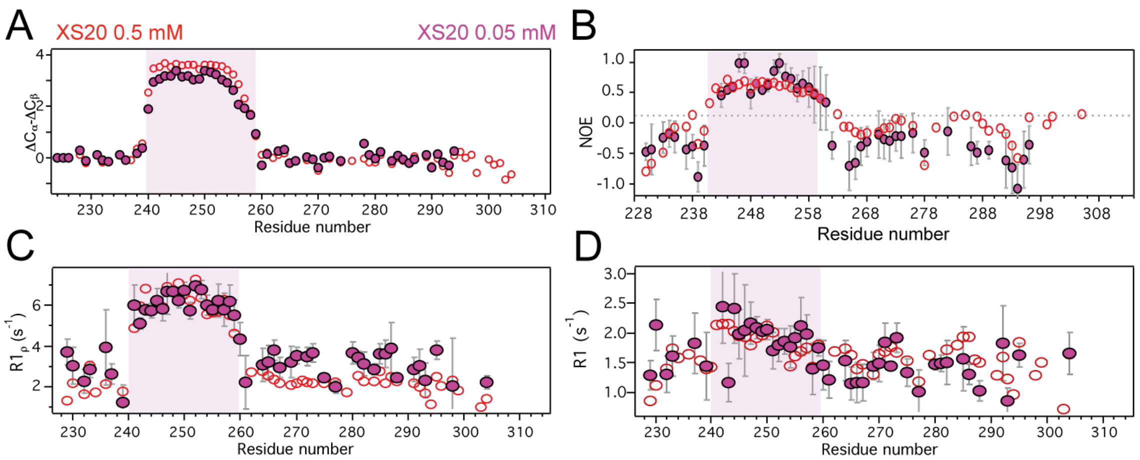

Supplementary Figure 6. Secondary chemical shifts and relaxation parameters for XS20 at lower concentration. A) Secondary chemical shifts plot for XS20 at 0.5 mM (red circles) and 0.05 mM (purple full circles). The color code is maintained in the figure. **B-D)** Heteronuclear NOE (**B**), R1ρ (**C**) and R1 (**D**) relaxation rates for XS20 in the mentioned conditions. Error bars are not included for XS20 at 0.5 mM for simplicity. Purple shade in the plots limit the α-helical region. All data included in this figure was obtained at 25 °C. Source data are provided as a Source Data file.

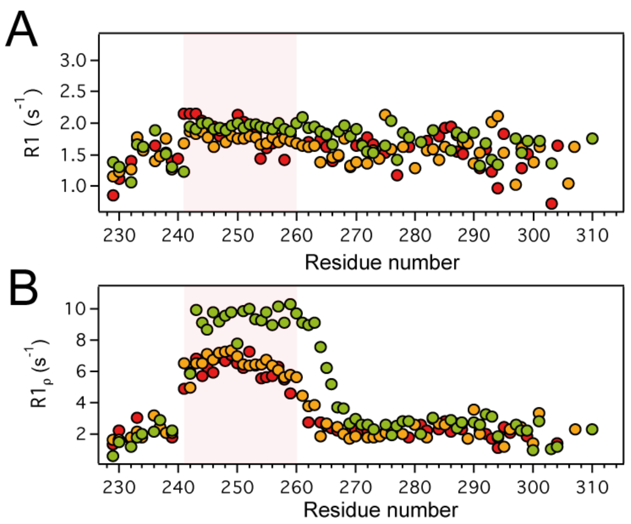

Supplementary Figure 7. PARMs promote intermolecular associations through the extended α-helix. A-B) ^{15}N spin relaxation parameters for XS20 (red), XS23 (orange) and XS26 (green) at 25 °C. Errors are lower than 1 s^{-1} (R1, **A**) and 0.8 s^{-1} (R1ρ, **B**) and error bars are not included for simplicity. The red shadows in the plots limit the α-helix in XS20. Source data are provided as a Source Data file.

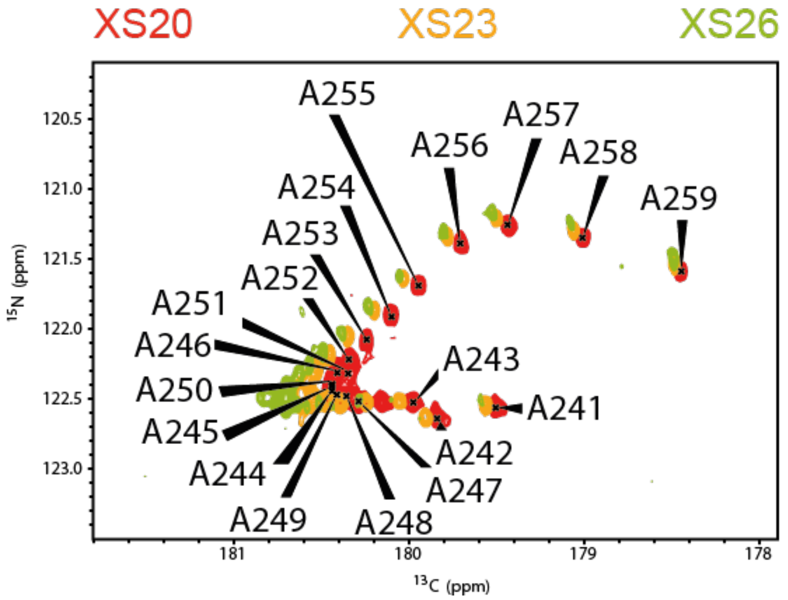

Supplementary Figure 8. The α-helix from the polyAla region is preserved in the PARMs. Shown is an overlay of the CON spectra for XS20 (red), XS23 (orange) and XS26 (green) at 25 °C. The spectra are focused on the polyAla region. Sequence-specific assignments refer to XS20. Spectra are identically referenced. Protein concentration was 0.5 mM.

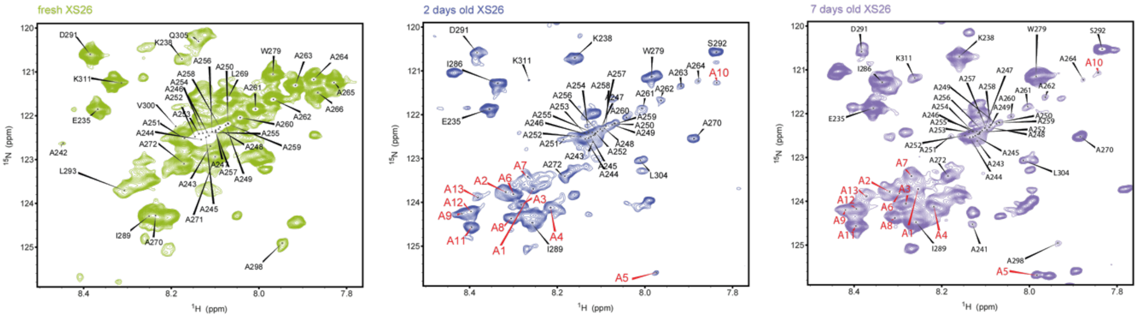

Supplementary Figure 9. The alternative polyAla conformations are reproducible and time-dependent. Detailed ^{15}N HSQC spectra from different XS26 samples (0.5 mM protein concentration) incubated at 25 °C for different times showing that the new Ala moieties (labeled in red) are reproducible and increase with the incubation time. The intensity of the α-helix polyAla crosspeaks decrease with incubation time. The new Ala crosspeaks are numbered arbitrarily. Only nascent Ala crosspeaks are labeled, assignments of additional crosspeaks that appear upon incubation are not labeled for simplicity. Contour levels were increased on the spectra on the right (7 days old XS26) to show the remaining signal intensity of the α-helix polyAla crosspeaks.

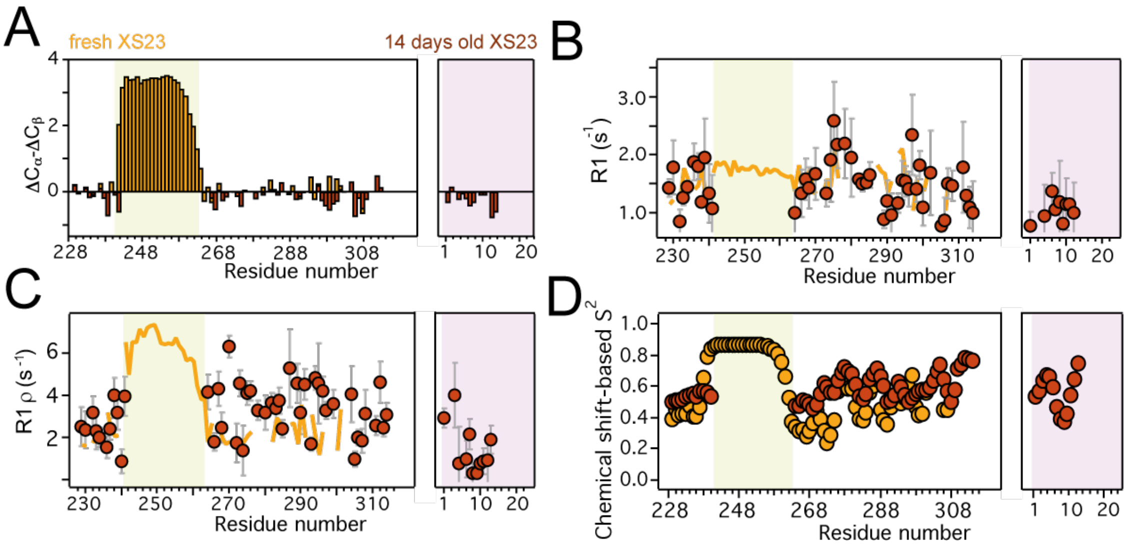

Supplementary Figure 10. PARMs adopt highly dynamic alternative conformations upon incubation. A) Secondary chemical shifts for fresh XS23 (orange bars) and for XS23 incubated for 14 days at 25 °C (brown bars). **B-C)** ^{15}N spin longitudinal (R_1, **B**) and rotating frame ($R_{1\rho}$, **C**) relaxation rates for fresh XS23 (orange line) and XS23 incubated for 14 days at 25 °C (brown markers). Error bars are not included in the orange lines for clarity. **D)** Chemical shift-based S^2 order parameters calculated by TALOS-N for fresh XS23 (orange markers) and XS23 incubated for 14 days at 25 °C (brown markers). The new alanine moieties appearing upon incubation at 25 °C are located on the right of the plots and display disordered conformations. Green shadow limits polyAla α-helix, while purple shadow limits nascent disordered alanine moieties. Protein concentration was 0.5 mM. Source data are provided as a Source Data file.

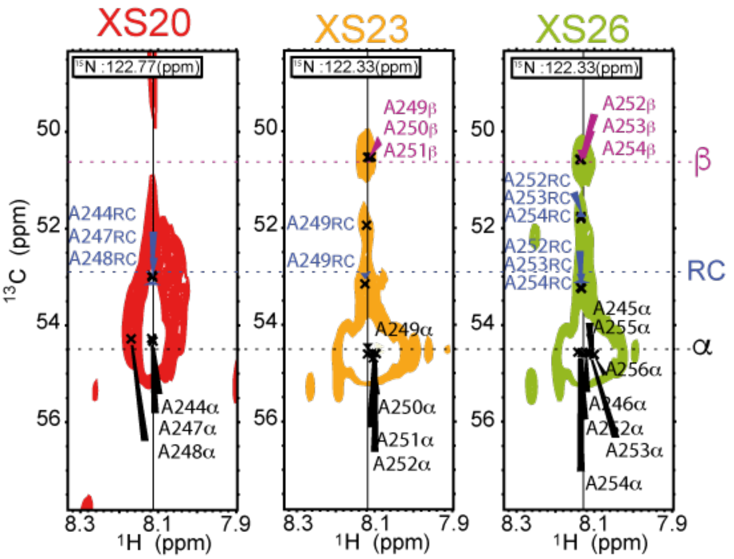

Supplementary Figure 11. PARMs promote structural conversions. Strips from the 3D CBCA(CO)NH spectra at 25 °C for XS20 (red), XS23 (orange) and XS26 (green) centered at the Cα positions. The chemical shifts for the ^{15}N-^{1}H dimensions correspond to alanine resonances belonging to the central segment of the polyAla tract (**Fig. 4A**). Crosspeaks with chemical shifts typical for α-helical conformations are labeled α (black), RC for disordered (for random coil, blue), and β for β-strand conformations (purple). Protein concentration was 0.5 mM.

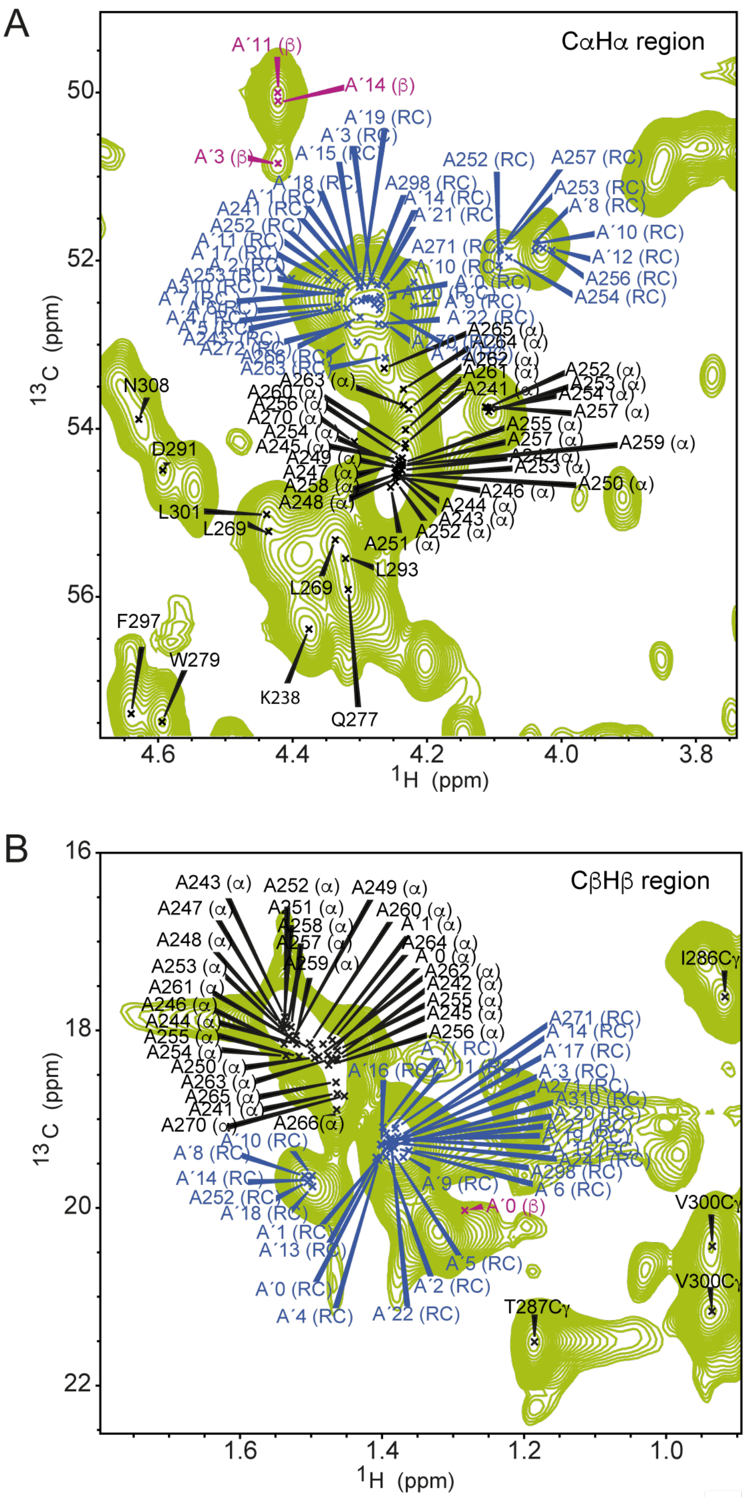

Supplementary Figure 12. ^{13}C-HSQC spectra reveal alternative polyAla conformations. A) Detail of the alanine moieties from the CαHα region of the ^{13}C-HSQC spectrum**. B)** Detail of the alanine moieties from the CβHβ region. α-helical conformations are labeled in black (α), disordered conformations (random coil, RC) are labeled in blue, and β-strand conformations are labeled in magenta (β). Sample was 0.5 mM XS26 after 24 h of incubation at 25 °C. New Alanine moieties are labeled with apostrophe and follow the numbering shown in **Supplementary Figure 9**.

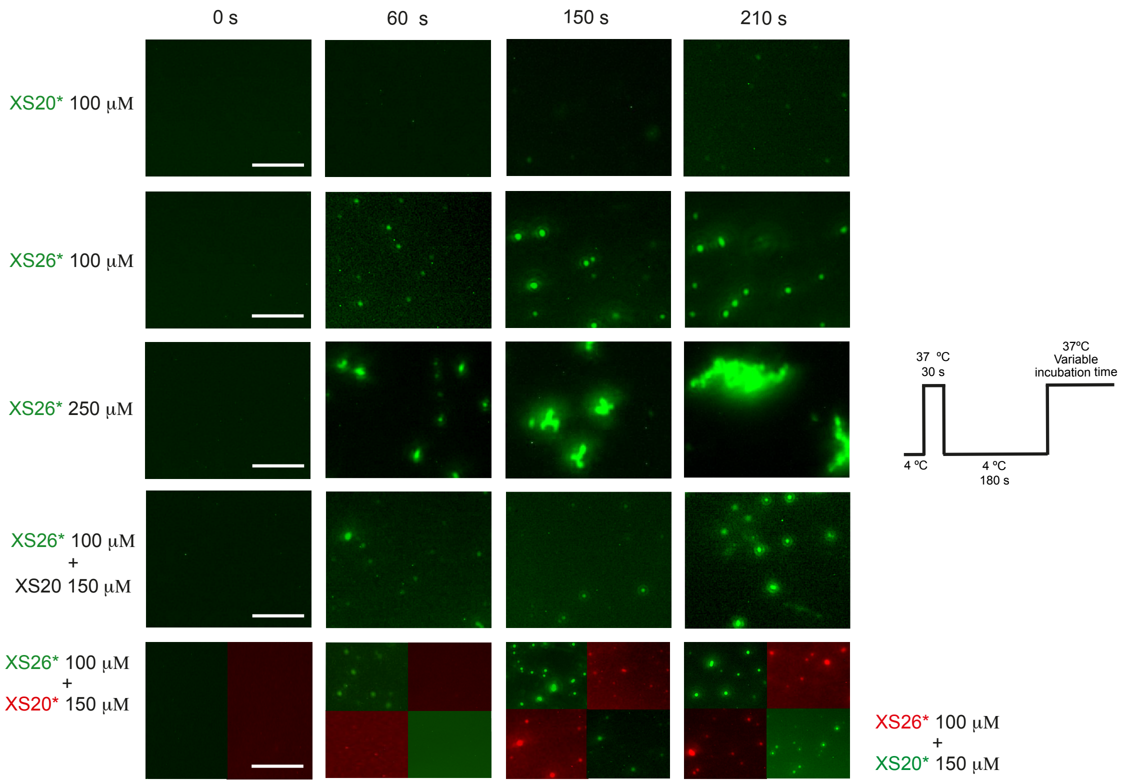

Supplementary Figure 13. XS26 triggers protein de-mixing and recruits XS20 into the condensates. Fluorescent-labeled XS20 and XS26 (labeled in red and green with asterisk where required) were included at 1:400-600 ratios in the mixtures. XS26 at 0.25 mM shows fast amorphous aggregation. Images containing both labeled proteins (lower row) do not correspond to the same sample fields. XS20 is incorporated into the condensates at a slower rate. Incubation procedure is indicated on the right. Scale bars correspond to 10 μm.

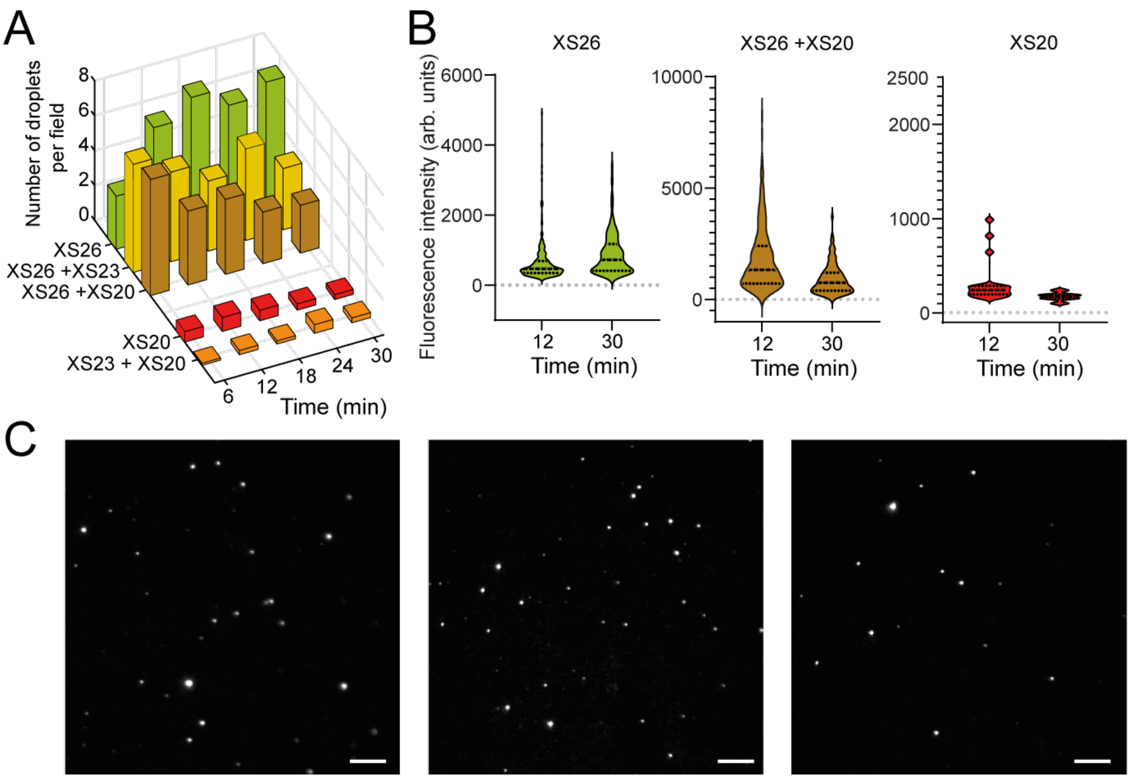

Supplementary Figure 14. Abundance and fluorescence intensity of the condensates. A) Histogram distributions of the number of condensates formed by the different mixtures with respect to the time spent for the microscopic observations. The field was defined by the microscope setup and was limited by an area of 50,070*50,070 μm. $n_{(XS26)}$= 1699; $n_{(XS26+XS23)}$= 1247; $n_{(XS26+XS20)}$= 1917; $n_{(XS20)}$= 66; $n_{(XS23+XS20)}$=66. **B)** Fluorescence intensity of the condensates formed by XS26 (left), XS26+XS20 (center) and XS20 (right) after 12 and 30 minutes of incubation. Protein concentration was 0.1 mM for XS26 and 0.15-0.25 mM for XS20. **C)** Representative images obtained by Nanoimager S microscope of the condensates formed by XS26 and included in the analyses. Scale bars= 5 μm. Source data are provided as a Source Data file.

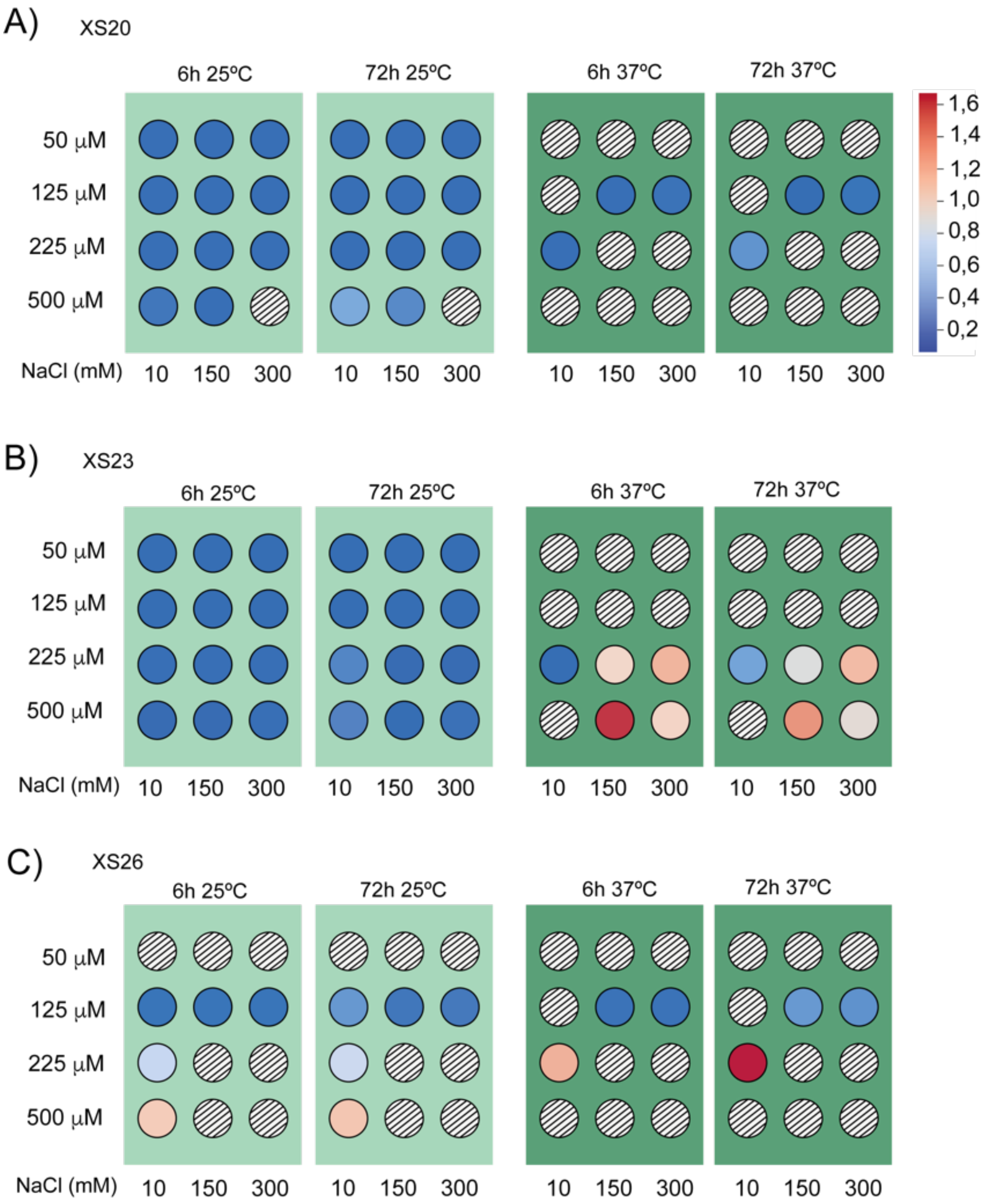

Supplementary Figure 15. Dot blot representation for the dependence of phase transitions on protein concentration, temperature an ionic strength. Heat-map scale on the right represents the average turbidity values for the mentioned conditions. While there is a clear dependence of phase transitions on protein concentration and temperature, there is no correlation with the ionic strength. Absorbance values (600 nm) were acquired after 6 and 72 h of measurement at the specific temperature. Striped blots indicate not acquired values. Source data are provided as a Source Data file.

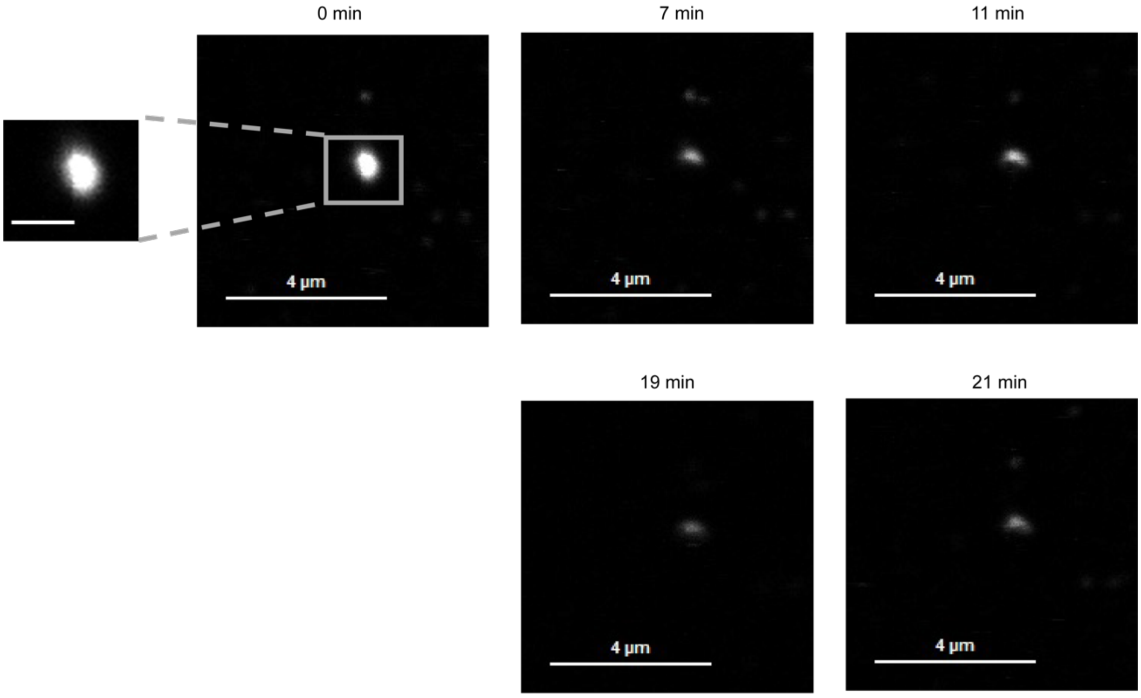

Supplementary Figure 16. Detail of a confocal-based FRAP experiment on a single condensate. No fluorescence intensity was recovered after 21 minutes following photobleaching. This condensate corresponds to XS26 incubated at 0.1 mM. The scale bar on the insert at the left corresponds to 1 μm.

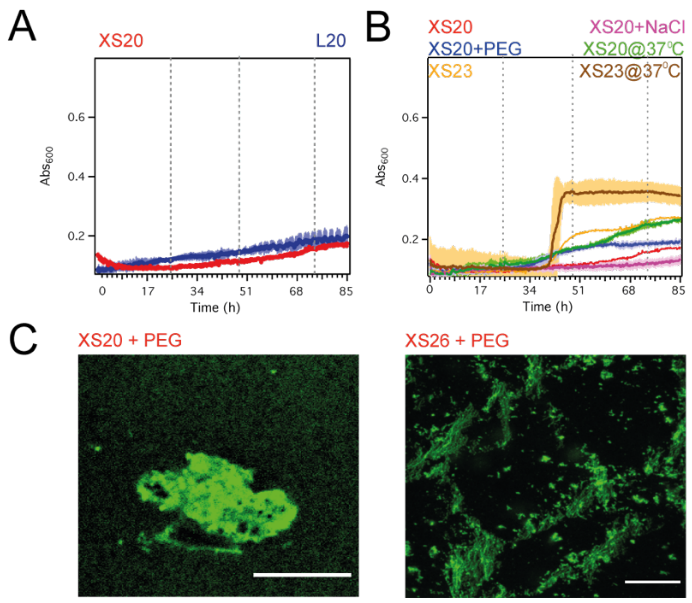

Supplementary Figure 17. Controls for PHOX2B phase transitions. A) XS20 recapitulates longer PHOX2B construct phase transitions. XS20 is represented in red and L20 in dark blue. **B)** Addition of 10 % PEG (w/v) accelerates phase transition kinetics (dark blue). Addition of 0.3 M NaCl has no effect on XS20 phase transitions (magenta), indicating that hydrophobic aggregation is not the driving force underlying phase transitions[2]. Incubation at 37 °C accelerates phase transition kinetics both for XS20 (green) and XS23 (dark brown), likely due to a temperature-dependent destabilization of polyAla α-helical conformation. XS20 (red) and XS23 (orange) turbidity plots are included for comparison. **C)** Representative superresolution images of the amorphous aggregates formed by XS20 (left) and XS26 (right) with 10 % PEG (w/v) after 210 s of incubation following the protocol indicated in **Supplementary Figure 13.** Scale bars= 10 μm. Proteins were labeled with ATTO-488. Protein concentration was 0.1 mM (**C**) and 0.225 mM (**A-B**) and incubation was performed at 25 °C except where noted. Vertical broken lines represent the consecutive days of incubation (**A, B**). Source data are provided as a Source Data file.

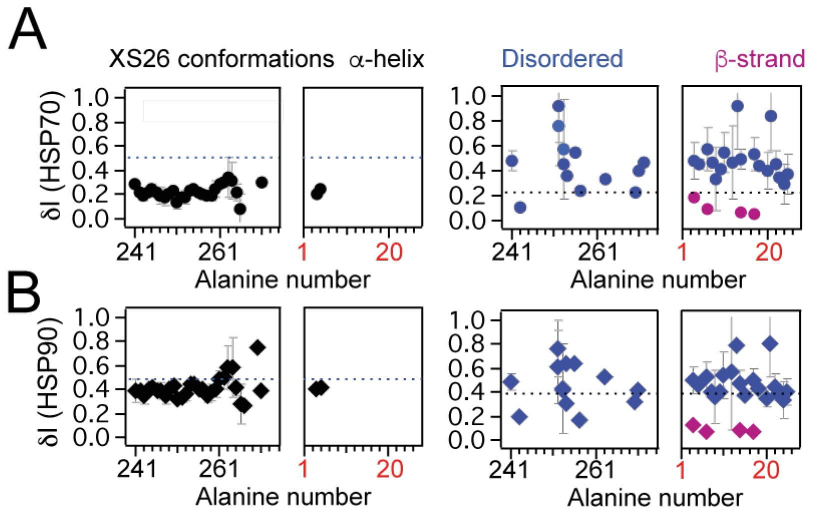

Supplementary Figure 18. Molecular chaperones target preferentially PHOX2B nascent conformations. A-B) Decay in XS26 (0.1 mM) ^{13}C-HSQC signal intensities upon addition of fresh HSP70 (**A**) and HSP90 (**B**, all in 1:1 molar ratios) for the different conformations of XS26 polyAla residues. Plots show α-helical (black), disordered (blue) and β-strand (magenta) polyAla conformations, and include averaged Cα and Cβ crosspeak intensities (**Supplementary Figure 12**). X-axis are arranged based on polyAla residue numbering (241-266) or on the nascent Ala (1-23, in red on the axes). Broken lines in the plots represent the average of the signals contained in the corresponding pairing plot, for comparison. Source data are provided as a Source Data file.

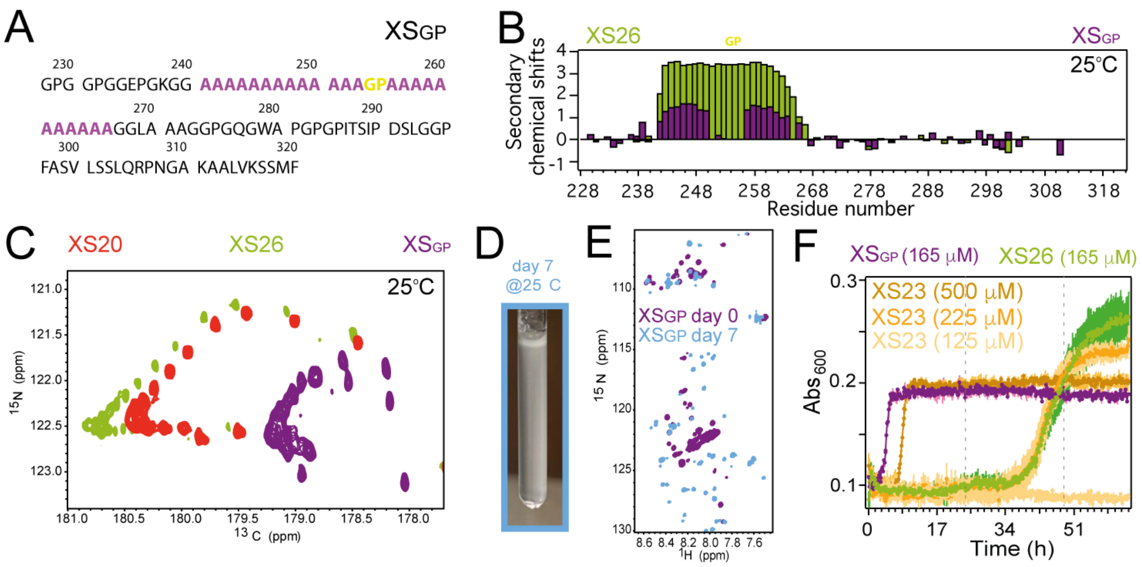

Supplementary Figure 19. Increased disorder by α-helix disruption accelerates phase transitions. A) XS_{GP} sequence. The polyAla tract is highlighted in magenta and the Gly-Pro insertion highlighted in bright green. **B)** Secondary chemical shifts plot for XS26 (green) and XS_{GP} (magenta) at 25 °C. The Gly-Pro insertion (located on top of the plot) disrupts the α-helix and promotes increased protein disorder. **C)** CON spectra for XS20 (red), XS26 (green) and XS_{GP} (magenta) at 25 °C focused on the polyAla tract. Lower CO chemical shifts for XS_{GP} indicate lower α-helical content. **D)** Representative image of a XS_{GP} NMR sample after 7 days of incubation at 25 °C showing turbidity. **E)** ^{15}N HSQC spectra for fresh XS_{GP} at 25 °C (magenta) and after 7 days of incubation at 25 °C (light blue). **F)** Turbidity at 25 °C for XS_{GP} (magenta), XS26 (green) and XS23 (dark to light orange) at the mentioned protein concentrations. XS_{GP} shows faster turbidity than XS26 at identical protein concentrations, and is only comparable to XS23 at significantly higher concentrations. Broken lines represent consecutive days of incubation. Protein concentration in the NMR experiments (**B**-**E**) was 0.5 mM. Source data are provided as a Source Data file.

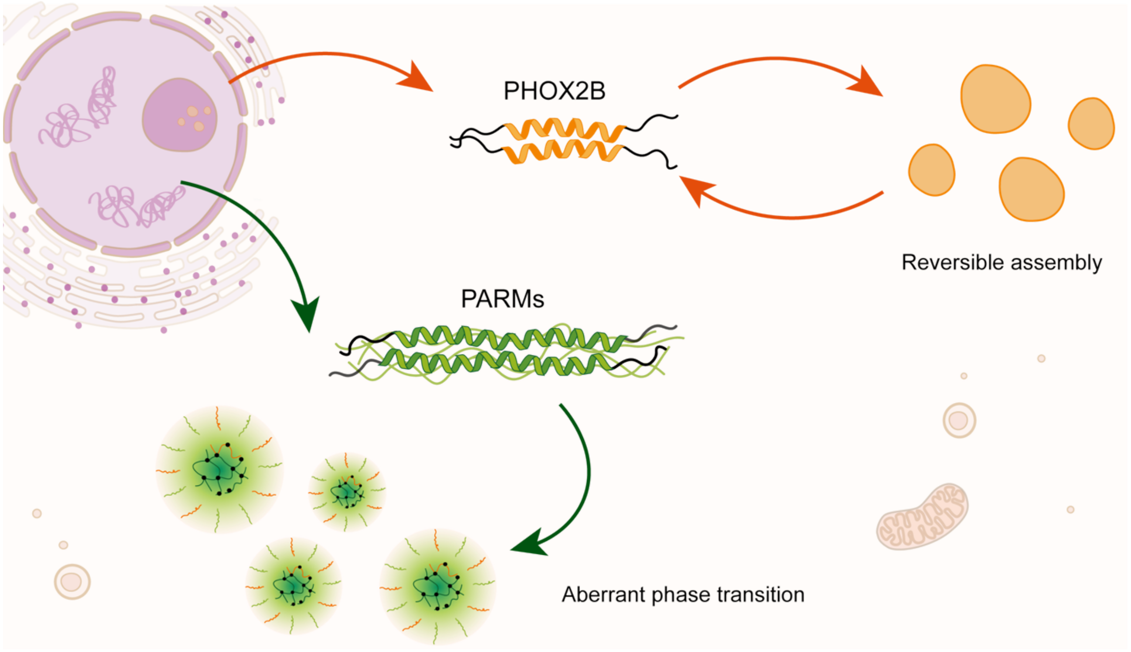

Supplementary Figure 20. Mechanistic model for the role of PARMs in PHOX2B pathogenicity. PHOX2B establishes intermolecular interactions through the polyAla segment which could trigger reversible oligomer formation, both in the cytosol and in the nucleolus[3]. PARMs induce metamorphism giving rise to disordered conformations which prime a fast phase transition into solid condensates. These solid condensates arrest wild-type PHOX2B, leading to PHOX2B loss-of-function and determining a dominant negative phenotype for CCHS. The condensates were depicted following the model proposed in[4].

	R_1 (s^{-1})		R_2 (s^{-1})		HETNOE		τ_C (ns)
	ALL	AAA	ALL	AAA	ALL	AAA	
XS20 (5°C)	1,89 +/- 0,40	1,54 +/- 0,19	6,1 +/- 4,5	11,8 +/- 2,7	0,42 +/- 0,21	0,67 +/- 0,08	6,7 (1,7)
XS20 (25°C)	1,62 +/- 0,31	1,83 +/- 0,25	3,5 +/- 2,0	5,9 +/- 1,2	0,12 +/- 0,40	0,53 +/- 0,18	3,6 (0,9)
XS20 (0.05 mM) (25°C)	NA	1,92 +/- 0,31	NA	6,1 +/- 0,5	NA	0,65 +/- 0,18	3,6
XS23 (25°C)	1,60+/- 0,23	1,73+/- 0,13	3,7+/- 2,1	6,3+/- 1,1	0,11+/- 0,47	0,59+/- 0,18	4,0 (NA)
XS26 (25°C)	1,75+/- 0,24	1,88+/- 0,21	4,9+/- 3,4	8,9+/- 1,9	0,18+/- 0,50	0,65+/- 0,12	4,7 (NA)
XS23i (25°C)	1,40+/- 0,42	1,11+/- 0,34	2,9+/- 1,5	2,1+/- 2,0	-1,18+/- 0,90	-0.77+/- 0,83	-

Supplementary Table 1. Correlation time and relaxation parameters for PHOX2B constructs. Data is presented considering either the whole sequence (ALL) or only the polyAla segment (AAA). Data is shown as calculated value +/- SD. Correlation time (τ_C) is presented based on experimental R_1 and R_2 rates[5] of the polyAla segments, and based on theoretical calculations using HydroNMR[6] on monomeric, experimentally-derived XS20 α-helical structures (for XS20, presented between parenthesis). HydroNMR-based theoretical τ_C were not calculated for XS23 and XS26 due to the lack of atomic structure for these constructs. The difference in τ_C for XS20 at 5 °C and 25 °C is consistent with the increase of solvent viscosity at lower temperatures. However, the disparity between experimental and theoretical τ_C values indicates that PHOX2B is assembling into small oligomeric species, regardless of the temperature and concentration. HydroNMR[6] postulates an increase in τ_C of 0,19 ns for a canonical α-helix upon addition of 3 residues. Therefore, the significant difference in τ_C for the expanded mutants, more especially for XS26, indicates the higher propensity to associate for the PARMs. XS23i refers to the relaxation data for incubated XS23 (**Supplementary Figure 10**). For XS23i, AAA refers to the nascent Ala moieties. Although no τ_C was calculated for XS23i due to the lack of structured elements, relaxation data clearly indicates that the emerging Ala belong to highly flexible fragments. Unless otherwise stated, protein concentration was 0.5 mM.

	Protein
**NMR distance and dihedral constraints**	
Distance constraints	
Total NOE	69
Intra-residue	19
Inter-residue	50
Sequential ($\|i-j\| = 1$)	33
Medium-range ($\|i-j\| < 4$)	5
Long-range ($\|i-j\| > 5$)	12
Intermolecular	
Hydrogen bonds	0
Total dihedral angle restraints	
ϕ	34
ψ	34
**Structure statistics**	
Violations (mean and s.d.)	
Distance constraints (Å)	0.01 ± 0.03
Dihedral angle constraints (º)	0.25 ± 0.13
Max. dihedral angle violation (º)	0.60
Max. distance constraint violation (Å)	0.16
Deviations from idealized geometry	
Bond lengths (Å)	0.007 ± 0.001
Bond angles (º)	1.6 ± 0.2
Impropers (º)	0 ± 0
Average pairwise r.m.s. deviation** (Å)	
Heavy	1.53
Backbone	1.46

**Pairwise r.m.s. deviation was calculated among 20 refined structures (relative to the polyAlanine segment 241-260).

Supplementary Table 2. Structure Calculation Statistics for the 3D NMR XS20 structures at 5 °C

	Protein
**NMR distance and dihedral constraints**	
Distance constraints	
Total NOE	44
Intra-residue	21
Inter-residue	23
Sequential ($\|i-j\|$ = 1)	17
Medium-range ($\|i-j\|$ < 4)	6
Long-range ($\|i-j\|$ > 5)	0
Intermolecular	
Hydrogen bonds	0
Total dihedral angle restraints	
ϕ	24
ψ	24
**Structure statistics**	
Violations (mean and s.d.)	
Distance constraints (Å)	0.00 ± 0.01
Dihedral angle constraints (º)	1.23 ± 0.11
Max. dihedral angle violation (º)	1.36
Max. distance constraint violation (Å)	0.04
Deviations from idealized geometry	
Bond lengths (Å)	0.007 ± 0.001
Bond angles (º)	1.5 ± 0.1
Impropers (º)	0 ± 0
Average pairwise r.m.s. deviation** (Å)	
Heavy	1.21
Backbone	1.15

**Pairwise r.m.s. deviation was calculated among 20 refined structures (relative to the polyAlanine segment 241-260).

Supplementary Table 3. Structure Calculation Statistics for the 3D NMR XS20 structures at 25 °C

Supplementary References

1. Shen Y, Bax A. Protein backbone and sidechain torsion angles predicted from NMR chemical shifts using artificial neural networks. *J Biomol NMR* **56**, 227-241 (2013).

2. Bernacki JP, Murphy RM. Length-dependent aggregation of uninterrupted polyalanine peptides. *Biochemistry* **50**, 9200-9211 (2011).

3. Mensah MA*, et al.* Aberrant phase separation and nucleolar dysfunction in rare genetic diseases. *Nature* **614**, 564-571 (2023).

4. Farag M, Cohen SR, Borcherds WM, Bremer A, Mittag T, Pappu RV. Condensates formed by prion-like low-complexity domains have small-world network structures and interfaces defined by expanded conformations. *Nat Commun* **13**, 7722 (2022).

5. Kay LE, Torchia DA, Bax A. Backbone dynamics of proteins as studied by 15N inverse detected heteronuclear NMR spectroscopy: application to staphylococcal nuclease. *Biochemistry* **28**, 8972-8979 (1989).

6. García de la Torre J, Huertas ML, Carrasco B. HYDRONMR: prediction of NMR relaxation of globular proteins from atomic-level structures and hydrodynamic calculations. *J Magn Reson* **147**, 138-146 (2000).
