# Supplementary material for: Alternative low-populated conformations prompt phase transitions in polyalanine repeat expansions
Source: Nat Commun. 2024 Mar 2;15:1925. doi: 10.1038/s41467-024-46236-5 (PMC10908835; doi:10.1038/s41467-024-46236-5)
Supplement: Supplementary file 3 — Reporting Summary [file 41467_2024_46236_MOESM3_ESM.pdf]

nature portfolio

Corresponding author(s): Dr. Javier Oroz

Last updated by author(s): Feb 12, 2024

Reporting Summary

Nature Portfolio wishes to improve the reproducibility of the work that we publish. This form provides structure for consistency and transparency in reporting. For further information on Nature Portfolio policies, see our Editorial Policies and the Editorial Policy Checklist.

Statistics

For all statistical analyses, confirm that the following items are present in the figure legend, table legend, main text, or Methods section.

n/a	Confirmed	
[ ]	[x]	The exact sample size (*n*) for each experimental group/condition, given as a discrete number and unit of measurement
[ ]	[x]	A statement on whether measurements were taken from distinct samples or whether the same sample was measured repeatedly
[x]	[ ]	The statistical test(s) used AND whether they are one- or two-sided<br>*Only common tests should be described solely by name; describe more complex techniques in the Methods section.*
[x]	[ ]	A description of all covariates tested
[x]	[ ]	A description of any assumptions or corrections, such as tests of normality and adjustment for multiple comparisons
[ ]	[x]	A full description of the statistical parameters including central tendency (e.g. means) or other basic estimates (e.g. regression coefficient) AND variation (e.g. standard deviation) or associated estimates of uncertainty (e.g. confidence intervals)
[x]	[ ]	For null hypothesis testing, the test statistic (e.g. *F*, *t*, *r*) with confidence intervals, effect sizes, degrees of freedom and *P* value noted<br>*Give P values as exact values whenever suitable.*
[x]	[ ]	For Bayesian analysis, information on the choice of priors and Markov chain Monte Carlo settings
[x]	[ ]	For hierarchical and complex designs, identification of the appropriate level for tests and full reporting of outcomes
[x]	[ ]	Estimates of effect sizes (e.g. Cohen's *d*, Pearson's *r*), indicating how they were calculated

Our web collection on statistics for biologists contains articles on many of the points above.

Software and code

Policy information about availability of computer code

Data collection	Topspin 4.1.1., ImageJ
Data analysis	Sparky 3.190, CYANA 3.98.13, ImageJ

For manuscripts utilizing custom algorithms or software that are central to the research but not yet described in published literature, software must be made available to editors and reviewers. We strongly encourage code deposition in a community repository (e.g. GitHub). See the Nature Portfolio guidelines for submitting code & software for further information.

Data

Policy information about availability of data

All manuscripts must include a data availability statement. This statement should provide the following information, where applicable:

- Accession codes, unique identifiers, or web links for publicly available datasets
- A description of any restrictions on data availability
- For clinical datasets or third party data, please ensure that the statement adheres to our policy

UniProt KB Q99453, [https://www.uniprot.org/uniprotkb/Q99453/entry] is included in the Methods section
Data availability statement: All materials are readily available from the corresponding author upon request. Source data are provided with this paper. The structural data generated in this study have been deposited in the PDB database under the accession codes 8PTL [http://doi.org/10.2210/pdb8ptl/pdb] and 8PUI [http://doi.org/10.2210/pdb8pui/pdb] and the chemical shifts data in the BMRB database under the accession codes 51978 [https://dx.doi.org/10.13018/BMR51978] and

51979 [https://dx.doi.org/10.13018/BMR51979].

Research involving human participants, their data, or biological material

Policy information about studies with human participants or human data. See also policy information about sex, gender (identity/presentation), and sexual orientation and race, ethnicity and racism.

Reporting on sex and gender	not applicable
Reporting on race, ethnicity, or other socially relevant groupings	not applicable
Population characteristics	not applicable
Recruitment	not applicable
Ethics oversight	not applicable

Note that full information on the approval of the study protocol must also be provided in the manuscript.

Field-specific reporting

Please select the one below that is the best fit for your research. If you are not sure, read the appropriate sections before making your selection.

- [x] Life sciences
- [ ] Behavioural & social sciences
- [ ] Ecological, evolutionary & environmental sciences

For a reference copy of the document with all sections, see nature.com/documents/nr-reporting-summary-flat.pdf

Life sciences study design

All studies must disclose on these points even when the disclosure is negative.

Sample size	17 proteins were generated in total, turbidimetry studies included 8 conditions, fluorescence microscopy included 9 conditions, NMR was performed on 21 different samples, CD on 3 different samples. This sample size (including variations in temperature, concentration and sequence mutations) is sufficient to provide a full description of the structural impact of PARMs in PHOX2B and its effect on phase transitions. In brief, all conditions are summarized in: turbidimetry conditions (25 degrees) and NMR conditions (5 degrees and 25 degrees, for fresh and incubated samples). Experimental buffer contained 5mM KPi/10mM NaCl/2mM bmercaptoethanol/0,03 % sodium azide [pH 6.8] except in those samples containing molecular chaperones and co-chaperones, where the buffer was 20 mM Hepes/5 mM MgCl2/10 mM KCl/1 mM DTT [pH 6.8]
Data exclusions	none
Replication	Turbidimetry and micrsocopy studies included technical and biological replicates (at least two biological replicates per condition for turbidimetry, at least two biological replicates per condition for microscopy), NMR experiments included several sample conditions and titration points. Each NMR experiment was performed independently. All attempts at replication (turbidimetry and microscopy) were successful. Statements on replication are included in the Methods section.
Randomization	allocation was random
Blinding	not applicable, conditions were predetermined and replicated

Reporting for specific materials, systems and methods

We require information from authors about some types of materials, experimental systems and methods used in many studies. Here, indicate whether each material, system or method listed is relevant to your study. If you are not sure if a list item applies to your research, read the appropriate section before selecting a response.

Materials & experimental systems

n/a	Involved in the study	
x		Antibodies
x		Eukaryotic cell lines
x		Palaeontology and archaeology
x		Animals and other organisms
x		Clinical data
x		Dual use research of concern
x		Plants

Methods

n/a	Involved in the study	
x		ChIP-seq
x		Flow cytometry
x		MRI-based neuroimaging

Plants

Seed stocks	not applicable
Novel plant genotypes	not applicable
Authentication	not applicable
