# Supplementary material for: Alternative low-populated conformations prompt phase transitions in polyalanine repeat expansions
Source: Nat Commun. 2024 Mar 2;15:1925. doi: 10.1038/s41467-024-46236-5 (PMC10908835; doi:10.1038/s41467-024-46236-5)
Supplement: Supplementary file 2 — Peer Review File [file 41467_2024_46236_MOESM2_ESM.pdf]

nature portfolio

Peer Review File

Alternative low-populated conformations prompt phase transitions in polyalanine repeat expansions

Open Access This file is licensed under a Creative Commons Attribution 4.0 International License, which permits use, sharing, adaptation, distribution and reproduction in any medium or format, as long as you give appropriate credit to the original author(s) and the source, provide a link to the Creative Commons license, and indicate if changes were made. In the cases where the authors are anonymous, such as is the case for the reports of anonymous peer reviewers, author attribution should be to 'Anonymous Referee' followed by a clear attribution to the source work. The images or other third party material in this file are included in the article's Creative Commons license, unless indicated otherwise in a credit line to the material. If material is not included in the article's Creative Commons license and your intended use is not permitted by statutory regulation or exceeds the permitted use, you will need to obtain permission directly from the copyright holder. To view a copy of this license, visit http://creativecommons.org/licenses/by/4.0/.

REVIEWER COMMENTS

Reviewer #1 (Remarks to the Author):

In this interesting manuscript, the authors propose that expansions of a polyAla tract in the C-terminal domain of protein PHOX2B trigger phase transitions that are associated with the disease congenital central hyperventilation syndrome. The authors reach this conclusion after analysing how the structural properties of a fragment of this domain depend on the length of the tract by solution nuclear magnetic resonance (NMR) and characterising the protein aggregates formed by this fragment in vitro.

The topic addressed by the authors is interesting, the results obtained are intriguing and, with further work, could indeed lead to an increased understanding of the molecular basis of a relatively rare disease. The work presented is not sufficient to support the main conclusion, however, and I therefore consider this manuscript not yet suitable for publication. In what follows I list several experiments that the authors should carry out to better understand how the length of the polyA tract alters the behaviour of the fragment and suggestions for improvement.

Major points

1 - The first relevant finding of this work is the observation of changes in the spectrum of XS20 after 1 day of incubation at 25 degrees, followed by an increase in turbidity 2 weeks later (Figure 2). Despite indications that XS20 oligomerises (see minor point 3), and despite the relatively slow rate of the process responsible for the changes, the authors did not investigate whether they are due to a conformational change in the monomer or, instead, are due to its oligomerisation. It would therefore be necessary for the authors to measure the rate of change as a function of concentration as well as carry out experiments in parallel to measure the oligomerisation state of the samples in a time-resolved fashion, potentially by diffusion NMR, DLS or, even better, both approaches.

2 - The authors observed that the samples of the fragments with expanded polyA tracts, upon incubation at 25 degrees, became turbid faster than XS20 and, in addition, that their NMR spectra displayed resonances corresponding to disordered Ala residues (Figure 4). As for major point 1, a correct interpretation of these observations requires that the authors determine whether the new resonances stem from monomers that have undergone a slow conformational change, which is unlikely, or whether they instead stem from an oligomeric species, potentially by diffusion NMR/DLS.

3 - The authors end the results part corresponding to the NMR experiments with the sentence “Therefore, PARMs promote alternative minor conformations … “ (lines 266-269). As it is written, this sentence suggests that the monomeric fragment undergoes a conformational change that occurs before oligomerisation/aggregation whereas there are many indications that the conformational change occurs in an oligomeric/aggregated state. The authors should carry out experiments to remove this ambiguity (see points above) and then be clear in their interpretation of the results.

4 - Whether the helical structure of the polyAla tracts is important for the conformational change, oligomerisation/aggregation of the different fragments studied should be addressed, if only to exclude the possibility that the changes are simply a consequence of the increased hydrophobicity caused by the expansion. This could be achieved by repeating some of the NMR and microscopy experiments after disrupting the helix by introducing secondary structure breakers (Pro, Gly).

5 - The time-resolved analysis of the turbidity of solutions of XS20, 23 and 26 indicates, in agreement with the results of fluorescence microscopy, that XS26 may phase separate but that XS20 and 23 do not (Figure 5). The characterisation of the potential phase transition of XS26 is, however, too preliminary. It would be necessary for the authors to characterise how the position of the phase equilibrium depends on solution conditions (protein concentration, temperature, ionic strength). Also, the authors should characterise the ageing process that they mention in line 286 by determining whether the condensates have, initially, the properties of fluids and whether these evolve as the condensates age, as referred to on a few occasions in the manuscript. Of note, the results shown in Extended Fig. 14, on XS20 and XS23, do not address these points as neither peptide appears to phase separate.

Minor points

1 - 102-103: what do the authors mean by “partial structural information” with regard to previous work on polyglutamine sequences ?

2 - 167-168: the authors present as a main result of this work a structure calculation for the polyAla tract based on main chain chemical shifts and NOEs, that shows that the tract is helical. Although useful one could argue that this is a trivial result given that Ala is the residue with the highest helical propensity. The authors should present the structure predicted by AlphaFold and the helical propensity predicted by an algorithm such as Agadir for the fragment studied experimentally.

3 - 159-160: the authors state that the correlation times obtained from an analysis of the relaxation rates suggest that the fragment studied by solution NMR may establish intermolecular interactions. What is the specific rationale for this statement ? In any case, the authors should investigate this hypothesis in detail by measuring NMR parameters (chemical shifts, relaxation rates) at different concentrations: in addition to confirming this hypothesis, this would allow identifying the residues involved in the interactions. This is particularly relevant given the effect that incubation at room temperature and polyAla expansions have on the spectrum of the fragment (see major points 1 and 2).

4 - 193-194: what do the authors mean by “increase in order parameter” in this sentence ?

5 - 219-22: the authors study equivalent fragments but with expanded polyAla tracts and observe, as expected, that the associated helices become longer and appear to have “a stronger propensity to establish length-dependent intermolecular association”. As in minor point 3 this needs to be studied, by NMR, by carrying out experiments at different concentrations.

6 - 230-231: the authors carry out heat denaturations of fragments with increasingly long polyAla tracts and claim that, whereas that of XS20 is reversible, those of XS23 and XS26 are not and instead lead to

the formation of aggregates rich in beta structure. The CD spectrum of XS23 upon refolding differs from that expected for an aggregated rich in beta structure and resembles more that of an alpha helix: the authors should deconvolute the contribution of different secondary structures to the CD spectra.

Reviewer #2 (Remarks to the Author):

The manuscript by Anton et al describes the structural features of a peptide fragment of the Phox2B protein (residues 228-314) that contains a poly-alanine sequence in a wildtype and disease-expanded form. The work shows by NMR that the poly-alanine is helical in both wild-type and disease-associated lengths. The work shows that the peptides self-associate into oligomers and condensates. All this behaviour of polyA is well-established knowledge by others cited by the authors. What appears to be novel however, is that there is an apparent switch from helix to disordered conformation in the long polyA lengths upon incubation, which coincides with a more mechanically rigid condensate state. It is proposed that this disordered condensate state may facilitate a further evolved conformational transition into amyloid structures from within that state. This point, if it is indeed real (as I have some questions on that below), would be an important advance to our understanding of how polyA sequences can lead to disease and help to reconcile some of the controversial and conflicting ideas about alpha-helix vs amyloid states of polyA condensates.

Key comments:

• How unequivocal is the point made from the NMR data there is a loss of helix and gain in disorder? Ie can the authors rule out the loss of helix arises from spectral broadening due to protein aggregation? Can the authors indicate how much of the helix is lost at the expense of disordered structure gained? Extra independent assessments on bulk average secondary structure should be considered, such as FTIR or CD under the same conditions as the NMR.

• The authors conclude that the new resonances seen representing the disordered alanines are highly dynamic. Isn’t this inconsistent with the other conclusion that the aged, expanded phox2B fragment forms solids?

• How specific are the effects of the chaperones to their chaperone activity? It is conceivable that non-specific binding interferes with the condensation, especially with the concentrations of chaperones used. Fig 6: Was there any ATP in the reaction? What happens if another "inert" protein was added, like BSA? What about the J-domain protein co-chaperone that is important for loading protein substrate to the Hsp70?

Other comments:

• With respect to the sentence spanning lines 107-110: I don't think it is a requirement that proteins form a beta-sheet structure when forming a liquid-solid transition. Ref27 suggested that polyA8 forms amyloid - but the simulations were done at high temperature (330 K). At physiological temperatures polyA8 forms an alpha-helix as shown by others and in this manuscript.

• With respect to the sentence spanning lines 224-225: I suggest the authors tone this down as it was

one study that had suggested that polyA can form coiled-coils under certain circumstances.
• With respect to the sentence spanning lines 228-229: I suggest the authors frame the arguments more clearly in context of what ref 7 did. That study looked at different sequences to what was done here.
• Line 345: It is unclear what exact point the authors mean in this sentence for “are stably transmitted to offspring”. Does this refer to the DNA sequence? A protein prion-like aggregate? Either way the relevance is not clearly linked to the logic.
• Line 383 statement “showed negative FRAP”. The logic is unclear since FRAP cannot be negative. Do the authors mean the proteins have no mobility as assessed by FRAP?
• Line 419 – do the authors mean “steady-state” instead of “steady”? Please clarify.
• With reference to the sentence between lines 413-416: The logic of the sentence appears incorrect since polyQ does not become disordered from the helical state - it becomes fibrillar and beta sheet from either a disordered or potentially helical state.
• With reference to the sentence between lines 416-418: Best to rephrase this sentence as the sentence implies prion-like domains refers to polyA.
• The section between lines 428 and 434 do not make sense to me. Wouldn't the logic be more sensical if polyA were stickers? Afterall this work and that of others have shown polyA self assembles into oligomers and larger condensates.
• With reference to the sentence between lines 432-435: The logic is unclear and unexplained as to why the prior observations on polyQ-driven assemblies disagree with the point made about polyA aggregation.
• Line 454: It is unclear why the authors are indicating GPG are stickers – the reference they cite to this effect does not as far as I can read indicate these residues to act as stickers. I would expect that they would be spacers, especially in this context.
• Figure 2C: Is the protein forming a gel halfway up the tube in Fig 2C? It is a little hard to see the details of what is happening in the image. I suggest the authors consider a different way to show the result.
• In Figure 3, I suggest that the temperature used to thermally denature the proteins be stated in the legend. The figure title suggests "irreversible" unfolding. But the methods indicate the results are formed after only a few minutes cool down. Would the alpha-helix return eventually (ie if it re-established equilibrium?)
• Figure 4B: In the graph, is the data showing the chemical shift for the incubated sample to be zero in the helical section? Or are there bars buried behind the green bars?
• For Fig 4D, I suggest the authors split the graph between the left and right parts because they are not plotting the same features on the x-axis. At first glance it appears the residue numbering on the right section applies to the main protein chain sequence rather than the arbitrary alanines denoted in panel A.
• I don't understand what is being plotted for the two colour samples in Extended Fig 11. Why are there not two colours taken for the same field of view?
• Statiscal issues: need to define the error bars in Fig 1E. How many samples were used to calculate the SD in Extended Table 1?

REVIEWER COMMENTS

Reviewer #1 (Remarks to the Author):

In this interesting manuscript, the authors propose that expansions of a polyAla tract in the C-terminal domain of protein PHOX2B trigger phase transitions that are associated with the disease congenital central hyperventilation syndrome. The authors reach this conclusion after analysing how the structural properties of a fragment of this domain depend on the length of the tract by solution nuclear magnetic resonance (NMR) and characterising the protein aggregates formed by this fragment in vitro.

The topic addressed by the authors is interesting, the results obtained are intriguing and, with further work, could indeed lead to an increased understanding of the molecular basis of a relatively rare disease. The work presented is not sufficient to support the main conclusion, however, and I therefore consider this manuscript not yet suitable for publication. In what follows I list several experiments that the authors should carry out to better understand how the length of the polyA tract alters the behaviour of the fragment and suggestions for improvement.

We thank the reviewer for her/his suggestions which indeed helped to improve the study.

Major points

1 - The first relevant finding of this work is the observation of changes in the spectrum of XS20 after 1 day of incubation at 25 degrees, followed by an increase in turbidity 2 weeks later (Figure 2). Despite indications that XS20 oligomerises (see minor point 3), and despite the relatively slow rate of the process responsible for the changes, the authors did not investigate whether they are due to a conformational change in the monomer or, instead, are due to its oligomerisation. It would therefore be necessary for the authors to measure the rate of change as a function of concentration as well as carry out experiments in parallel to measure the oligomerisation state of the samples in a time-resolved fashion, potentially by diffusion NMR, DLS or, even better, both approaches.

The reviewer raised an interesting point which was probably not sufficiently clear in the previous version of the manuscript. Indeed, understanding the nature of the species that give rise to the spectral changes is relevant for the interpretation of the data. In Figure 2, we show changes in the CON spectra of XS20 after 1 day of incubation giving rise to alternative conformations with lower helical content (lower CO chemical shift values, Figure 2B). In addition, both the changes in peak Intensity (Fig. 2D) and secondary chemical shifts (Fig. 2E) indicate that the protein is undergoing a time-dependent oligomerisation (due to the general loss in Intensity, Fig. 2D) and loss of α-helical content in the polyAlanine segment (lower values in Fig. 2E). Both increase in disorder (or loss of structured content, Fig. 2E) and oligomerization (or phase transitions) are coupled, as can be concluded from the new GlyPro mutant data (see major point #4 below) and Figure 6: molecular chaperones impede disordered conformations and block phase transitions. Therefore, the spectral changes observed in Figure 2 require high protein concentration to ensure that XS20 is able to generate sample turbidity (phase transitions). In particular, the changes observed in the incubated XS20 spectrum shown in Fig. 2 correspond to 0.5 mM protein samples. As determined in the phase diagram included in the new version of the manuscript (new Extended Figure 15, see major point #5 below), lower XS20 concentrations do not display phase transitions, and therefore no changes in the NMR spectra would be expected upon incubation if the protein concentration was lower than 0.5 mM. In addition, the CON spectra displayed in Fig. 2B requires a significant amount of protein concentration for detection, limiting the possibility of performing a concentration dependence study.

However, as the reviewer states, it is relevant to determine if the incipient crosspeaks in the NMR spectra from the incubated samples (Fig 2B, Figure 4 for XS26) origin from similar molecular species or from the assembly of larger oligomeric species. To determine the molecular entities producing the new crosspeaks in the NMR spectra, one could use relaxation data to calculate correlation times or, as the reviewer suggests, diffusion NMR to calculate diffusion coefficients and apparent molecular weight. Calculation of the correlation time for such disordered and dynamic structures may be prone to error using the Lipari-Szabo ModelFree formulism (Lipari, G.; Szabo, A. Model-Free Approach to the Interpretation of Nuclear Magnetic Resonance Relaxation In Macromolecules 1. Theory and Range of Validity; J. Am. Chem. Soc. 1982, 104, 4546). Still, as recommended by Nicholson etc (Pawley, N. H., Wang, C., Koide, S., and Nicholson, L. K. (**2001**) An improved method for distinguishing between anisotropic tumbling and chemical exchange in analysis of ^{15}N relaxation parameters J. Biomol. NMR 20, 149– 165) we employed the structured elements (polyAlanine α-helices) contained in the different constructions and calculated correlation times for XS20 at different concentrations and for XS23 and XS26. As shown in Extended Table 1, the correlation times for the ordered α-helical tract do not display a concentration dependence and are rather large (3,6 ns) for a 20 residue α-helix (or 23- and 26-residue helices for XS23 and XS26, respectively), which suggests

that XS20, XS23 and XS26 in solution are mainly not monomeric. Moreover, correlation times increase with the increasing length of the PARM (4,0 and 4,7 ns for XS23 and XS26, respectively), which is in agreement with the higher propensity of the expanded mutants to assemble at identical protein concentration (Fig. 5A).

Still, even considering that fresh XS20 in solution is oligomeric, the nascent NMR crosspeaks appearing upon incubation could belong to larger oligomeric species. As shown in Extended Figure 3 (included below for clarity) XS20 displays a concentration- and time-dependent oligomerization, which can be concluded from the signal decay observed in the 1D NMR projections, in agreement with the intensity decay plot in Figure 2D. In addition, following the reviewer´s suggestion, we performed diffusion NMR measurements on XS20 at different concentrations (0.05, 0.1 and 0.5 mM protein concentration) and after incubation to observe any potential difference in the diffusion coefficients in the assembled species in solution upon incubation, which could indicate large oligomer assembly. Although one should be cautious with the interpretation of the diffusion data since an increase in disorder upon incubation would strongly impact the dynamic radius of the protein affecting the diffusion coefficient, we obtained higher diffusion coefficients upon incubation, which cannot account for oligomer assembly. In addition, measurements in 8M urea (after correction for solvent viscosity), also showed increased diffusion coefficients, which could be interpreted as oligomer disassembly. As shown in the new Extended Figure 3, the diffusion coefficients do not differ substantially in the different XS20 concentrations (which retain identical structure, see below response to minor point #3, page 6). All these observations indicate that the large XS20 oligomeric assemblies lay beyond NMR detection, and therefore the observable NMR moieties (both the main conformation peaks and the nascent crosspeaks, Figs. 2B, 4A, 4C for XS26) belong to species of similar assembly order.

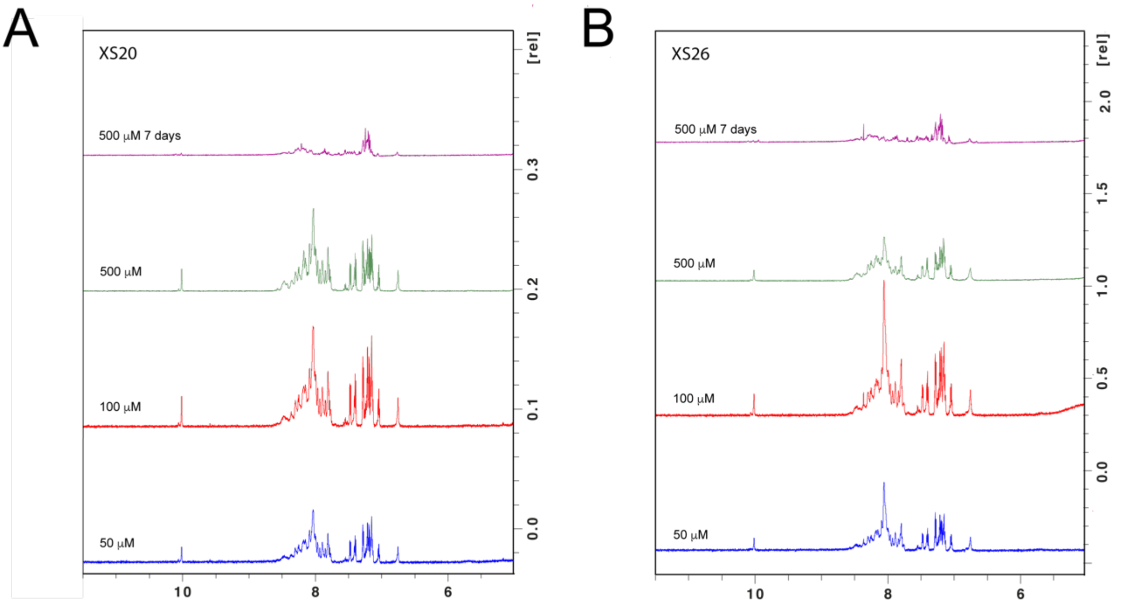

C

	D (m^2s^{-1})	
	0 days	7 days
XS20 50 μM	1,324 E-10	
XS20 100 μM	1,478 E-10	
XS20 500 μM	1,409 E-10	1,81 E-10

D

	D (m^2s^{-1})	
	0 days	7 days
XS26 50 μM	1,432 E-10	
XS26 100 μM	1,370 E-10	
XS26 500 μM	1,451 E-10	2,22 E-10
XS26 100 μM Urea	1,577 E-10	

Extended Figure 3. Concentration and incubation dependence of XS20 and XS26 NMR signals and diffusion coefficients. A) 1D NMR spectra for XS20 at 0.05 (blue), 0.1 (red), 0.5 mM (green) and 0.5 mM after 7 days of incubation at 25 °C (magenta). While signal intensity at 0.1 mM increases relative to 0.05 mM, the signal intensity at 0.5 mM decreases, indicating oligomerization. Upon incubation at high protein concentration, the signal intensity significantly decreases due to large oligomer assembly or aggregation. **B)** 1D NMR spectra for XS26 at 0.05 (blue), 0.1 (red), 0.5 mM (green) and 0.5 mM after 7 days of incubation at 25 °C (magenta). The signal intensity decay for XS26 at 0.5 mM (green) is more evident than for XS20 at 0.5 mM (**A**), in agreement with XS26 stronger assembly propensities. **C, D)** Diffusion coefficients for XS20 (**C**) and XS26 (**D**) at the mentioned protein concentrations and incubation time. Values indicate that the observable NMR signals arise from similar assembly species in all conditions tested. Diffusion coefficient in 8 M urea was corrected for the higher solvent viscosity. The sharp peak around 8 ppm in (**A, B**) correspond to the polyAlanine α-helical tract. No additional sharp peaks appear after incubation, indicating lack of protein degradation. Spectra contained identical magnification.

To our knowledge, this is the first experimental evidence of protein conformational changes coupled to phase transitions. In addition, the particular shifts from structured towards disordered conformations are in agreement with recent studies showing extreme protein dynamics at the interface (Farag M, et al. Condensates formed by prion-like low-complexity domains have small-world network structures and interfaces defined by expanded conformations. Nat Commun. 2022;13(1):7722. doi: 10.1038/s41467-022-35370-7. PMID: 36513655; PMCID: PMC9748015.) and within condensates (Galvanetto N, et al. Extreme dynamics in a biomolecular condensate. Nature. 2023;619(7971):876-883. doi: 10.1038/s41586-023-06329-5. PMID: 37468629).

This new data and the discussion are included in the modified version of the manuscript.

2 - The authors observed that the samples of the fragments with expanded polyA tracts, upon incubation at 25 degrees, became turbid faster than XS20 and, in addition, that their NMR spectra displayed resonances corresponding to disordered Ala residues (Figure 4). As for major point 1, a correct interpretation of these observations requires that the authors determine whether the new resonances stem from monomers that have undergone a slow conformational change, which is unlikely, or whether they instead stem from an oligomeric species, potentially by diffusion NMR/DLS.

This point is closely related to the previous issue raised. Remarkably, relaxation data for XS26 shows that the correlation time calculated for XS26 (which contains a +6 Ala extension) is significantly larger than that for the shorter versions (4,7 ns for XS26 vs. 3,6 ns for XS20, see Extended Table 1), which suggests that XS26 in solution displays a higher tendency to oligomerize. As explained above (Extended Figure 3), assembly into large oligomers significantly broadens NMR signals beyond detection. Therefore, observable NMR signals must origin from similar assembly species. Following the reviewer´s suggestions, we also performed diffusion NMR experiments using XS26 at different concentrations (0.05, 0.1 and 0.5 mM) to observed any potential reduction (due to large oligomer assembly) in the diffusion coefficients upon incubation (Extended Figure 3). As explained in the previous point, all the evidences indicate that NMR signals, including those corresponding to the main and nascent conformations, stem from oligomeric species, as the reviewer suggests.

3 - The authors end the results part corresponding to the NMR experiments with the sentence “Therefore, PARMs promote alternative minor conformations … “ (lines 266-269). As it is written, this sentence suggests that the monomeric fragment undergoes a conformational change that occurs before oligomerisation/aggregation whereas there are many indications that the conformational change occurs in an oligomeric/aggregated state. The authors should carry out experiments to remove this ambiguity (see points above) and then be clear in their interpretation of the results.

From the reviewer´s comments it is apparent that the observations were not exposed in a sufficiently clear manner in the previous version of the manuscript. The NMR data indicates that, even at low concentrations (see Extended Table 1), all the protein variants are oligomeric in solution. The nascent disordered conformations that appear upon incubation are coupled to aggregation and correspond to changes in species of similar assembly order. Therefore, we do not interpret the data as conformational transitions occurring in monomeric fragments before oligomerization, but rather as disordered conformers in small assembled species which are coupled to aggregation. In line with this, following the reviewer´s suggestions we have performed diffusion NMR experiments which has helped to clarify the interpretation of the data (see points above). We have made an effort to clarify these points raised in the modified version of the manuscript and figures.

4 - Whether the helical structure of the polyAla tracts is important for the conformational change, oligomerisation/aggregation of the different fragments studied should be addressed, if only to exclude the possibility that the changes are simply a consequence of the increased hydrophobicity caused by the expansion. This could be achieved by repeating some of the NMR and microscopy experiments after disrupting the helix by introducing secondary structure breakers (Pro, Gly).

This point raised by the reviewer is very interesting. Concluding that the increased aggregation propensities of the expanded Ala mutants is due to increased hydrophobicity would follow a logic rationale. Based on the available knowledge on polyAla repeats, α-helices are expected to follow a coiled-coil scheme of assembly driven by hydrophobicity. However, one of the key messages of the manuscript precisely refutes that paradigmatic aggregation scheme. We state that the phase transitions promoted by the PARMS are not due to the stacking of the polyAla α-helices driven by hydrophobicity, but rather due to an enhanced flexibility of the polyAla tracts that facilitate contacts between the sticking elements in the sequences flanking the polyAla fragment (see response at page 11-12 of this document). Indeed, the reviewer´s suggestion to generate a

mutant that breaks the α-helix and therefore promotes increased disorder clearly demonstrates that disordered conformations trigger phase transitions in PHOX2B.

Using XS26 sequence as template, we have generated a mutant were 2 Ala residues from the middle of the tract were replaced by Gly and Pro residues. Therefore, the $(Ala)_{26}$ tract was replaced by $(Ala)_{13}$-GlyPro-$(Ala)_{11}$. As shown in the figure below (Extended Figure 19), the mutation not only breaks the α-helix, but significantly reduces the population of structured conformers (as observed from the lower values in secondary chemical shift plot and CO chemical shifts in the CON spectra, Extended Figure 19B, C). This increased disorder clearly accelerates phase transitions, observed in turbidity measurements (Extended Figure 19D-F). This figure and its discussion are included in the modified version of the manuscript.

In addition, we have performed additional turbidity measurements at different protein concentrations, ionic strength and temperatures (discussed in detail in the following point). As shown below, there is no clear salt dependence in the phase transitions, which indicates that hydrophobicity is not the major driving force for PARM assembly. Still, the increased turbidity observed at higher temperatures indicates that reduced stability of the α-helical polyAla segment promotes phase transitions.

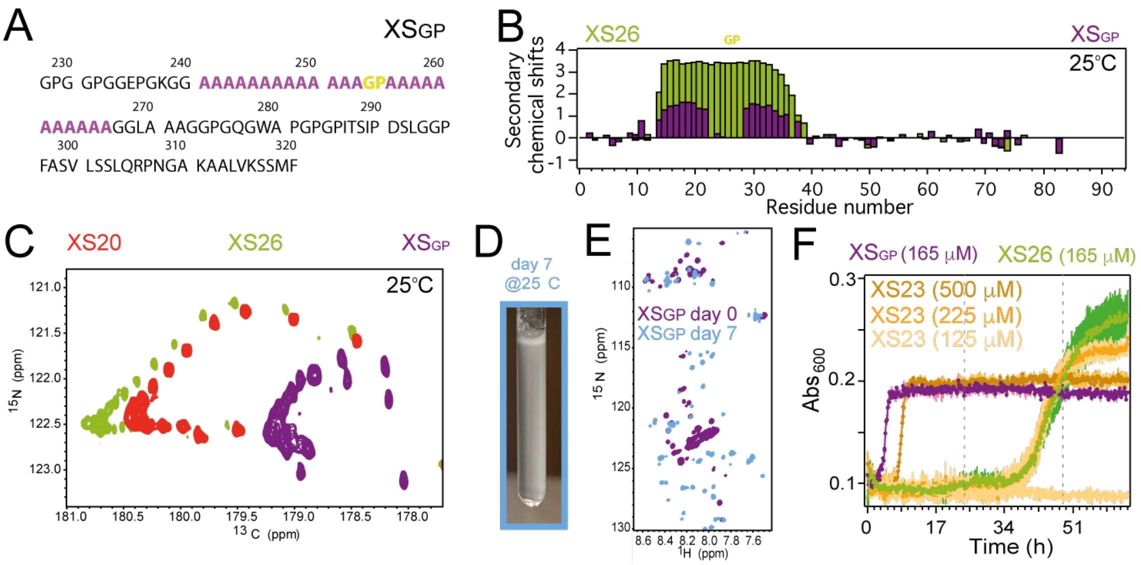

Extended Figure 19. Increased disorder by α-helix disruption accelerates phase transitions. A) XS_{GP} sequence. The polyAlanine tract is highlighted in magenta and the GlyPro insertion highlighted in bright green. **B)** Secondary chemical shifts plot for XS26 (green) and XS_{GP} (magenta) at 25 °C. The GlyPro insertion (located on top of the plot) disrupts the α-helix and promotes increased protein disorder. **C)** CON spectra for XS20 (red), XS26 (green) and XS_{GP} (magenta) at 25 °C focused on the polyAlanine tract. Lower CO chemical shifts for XS_{GP} indicate lower α-helical content. **D)** Representative image of a XS_{GP} NMR sample after 7 days of incubation at 25 °C showing turbidity. **E)** ^{15}N HSQC spectra for fresh XS_{GP} at 25 °C (magenta) and after 7 days of incubation at 25 °C (light blue). **F)** Turbidity at 25 °C for XS_{GP} (magenta), XS26 (green) and XS23 (dark to light orange) at the mentioned protein concentrations. XS_{GP} shows faster turbidity than XS26 at identical protein concentrations, and is only comparable to XS23 at significantly higher concentrations. Broken lines represent consecutive days of incubation. Protein concentration in the NMR experiments (**B**-**E**) was 0.5 mM.

5 - The time-resolved analysis of the turbidity of solutions of XS20, 23 and 26 indicates, in agreement with the results of fluorescence microscopy, that XS26 may phase separate but that XS20 and 23 do not (Figure 5). The characterisation of the potential phase transition of XS26 is, however, too preliminary. It would be necessary for the authors to characterise how the position of the phase equilibrium depends on solution conditions (protein concentration, temperature, ionic strength). Also, the authors should characterise the ageing process that they mention in line 286 by determining whether the condensates have, initially, the properties of fluids and whether these evolve as the condensates age, as referred to on a few occasions in the manuscript. Of note, the results shown in Extended Fig. 14, on XS20 and XS23, do not address these points as neither peptide appears to phase separate.

Following the reviewer´s recommendation, we have monitored in detail the phase transition of XS20, XS23 and XS26 at different protein concentrations, temperature and ionic strength. As shown in the new Extended Figure 15 in the modified version of the manuscript (included below for clarity), there is a clear concentration and temperature dependence in phase transitions. Higher temperatures accelerate phase transitions, while

there is a clear change of phase at high concentration (500 μM) and long incubation times for XS20 (in agreement with the NMR spectral changes discussed above and in Fig. 2). For XS23, the concentration that determines phase transitions is lower (225 μM), significantly accelerated at higher temperatures. Finally, XS26 shows phase transitions at lower concentrations (125 μM), in close agreement with the data showed in Figure 5 and Extended Figure 13. In agreement with the data obtained with the new GlyPro mutant discussed above, our interpretation for the dependence on the temperature is that higher temperatures promote helical instability, which triggers phase transitions. As shown in Figure 2, at low temperatures (5 °C) no turbidity is observed. However, we do not see a clear correlation with ionic strength for any construct, indicating that hydrophobicity is not the main driving force for condensate formation.

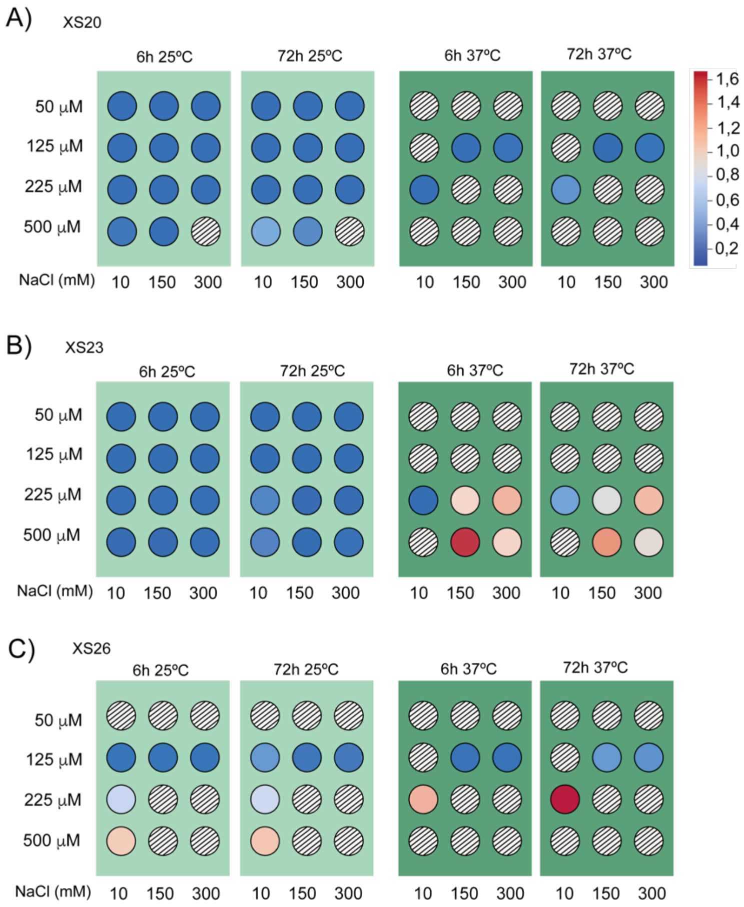

Extended Figure 15. Dot blot representation for the dependence of phase transitions on protein concentration, temperature an ionic strength. Heat-map scale on the right represents the average turbidity values for the mentioned conditions. While there is a clear dependence of phase transitions on protein concentration and temperature, there is no correlation with the ionic strength. Absorbance values (600 nm) were acquired after 6 and 72 h of measurement at the specific temperature. Striped blots indicate not acquired values.

This figure and its discussion are included in the modified version of the manuscript.

Regarding the ageing process of the condensates, we have performed time evolution characterization of the condensates, with image analysis every 6 minutes (detailed in the Material & Methods section). In Figure 5D, we averaged the FRAP values obtained at every time point for the different condensates. Even at time 6 (dead time of our measurements) the condensates formed in all conditions showed absence of FRAP, indicating minimal fluidity. Therefore, we conclude that there is a fast liquid-to-solid transition during condensate formation. We have clarified this point in Figure 5 legend.

Minor points

1 - 102-103: what do the authors mean by "partial structural information" with regard to previous work on polyglutamine sequences ?

A recent study on long polyglutamine polypeptides using selective labeling with fluorinated compounds has shown length-dependent α-helix formation based on NMR variables (Elena-Real, C.A., Sagar, A., Urbanek,

A. *et al.* The structure of pathogenic huntingtin exon 1 defines the bases of its aggregation propensity. *Nat Struct Mol Biol* **30**, 309–320 (2023). https://doi.org/10.1038/s41594-023-00920-0). This conclusion is in agreement with previous data showing dependence on the flanking residues for stabilization of a polyQ α-helix (Escobedo, A., Topal, B., Kunze, M.B.A. *et al.* Side chain to main chain hydrogen bonds stabilize a polyglutamine helix in a transcription factor. *Nat Commun* **10**, 2034 (2019). https://doi.org/10.1038/s41467-019-09923-2). Previously, data obtained at very low pH for a short polyQ tract indicated a pH dependence in the helical stability (Maria Baias, Pieter E. S. Smith, Koning Shen, Lukasz A. Joachimiak, Szymon Żerko, Wiktor Koźmiński, Judith Frydman, and Lucio Frydman. Structure and Dynamics of the Huntingtin Exon-1 N-Terminus: A Solution NMR Perspective. *Journal of the American Chemical Society* **2017** *139* (3), 1168-1176. DOI: 10.1021/jacs.6b10893). Nonetheles, despite these relevant studies, there is still no atomic level structure for any polyQ-containing protein. Indeed, the structures reported in our study represent, to our knowledge, the first protein homorepeats atomic structures described. Regarding the reviewer´s comment, we have replaced “partial structural information” with “limited structural information”, for clarity.

2 - 167-168: the authors present as a main result of this work a structure calculation for the polyAla tract based on main chain chemical shifts and NOEs, that shows that the tract is helical. Although useful one could argue that this is a trivial result given that Ala is the residue with the highest helical propensity. The authors should present the structure predicted by AlphaFold and the helical propensity predicted by an algorithm such as Agadir for the fragment studied experimentally.

We agree with the reviewer when stating that the α-helical structure for the polyAlanine tract could be predicted. However, the structures included in the study are the first experimental structures of protein homorepeats ever described, and were crucial for the interpretation of the relaxation data. This type of information demonstrates unambiguously that PARMS propagate identical structures in terms of conformation and rigidity, which is different from the polyQ case (see Elena-Real, C.A., Sagar, A., Urbanek, A. *et al.* The structure of pathogenic huntingtin exon 1 defines the bases of its aggregation propensity. *Nat Struct Mol Biol* **30**, 309–320 (2023). https://doi.org/10.1038/s41594-023-00920-0). As requested, we include below the AlphaFold structure predicted for XS20 and the Agadir prediction. We decided not to include these predictions in the manuscript due to the high amount of figures already included.

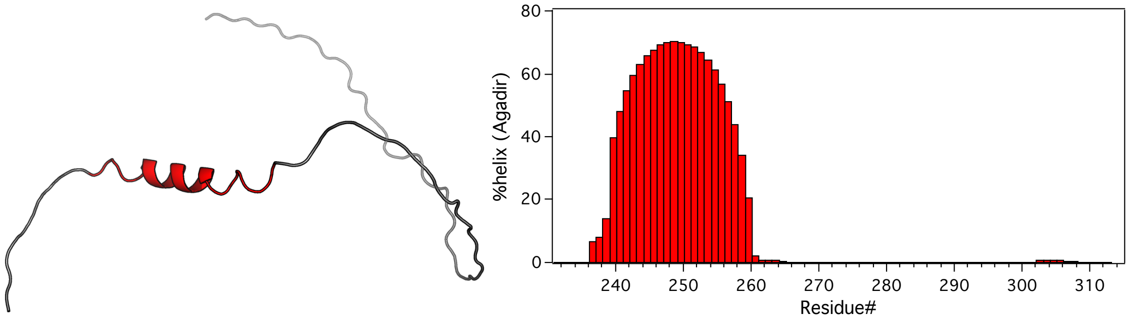

AlphaFold (left) and Agadir (right) predictions for XS20 at the NMR experimental conditions (25 °C). The polyAlanine tract is colored in red in the AlphaFold predicted structure.

3 - 159-160: the authors state that the correlation times obtained from an analysis of the relaxation rates suggest that the fragment studied by solution NMR may establish intermolecular interactions. What is the specific rationale for this statement ? In any case, the authors should investigate this hypothesis in detail by measuring NMR parameters (chemical shifts, relaxation rates) at different concentrations: in addition to confirming this hypothesis, this would allow identifying the residues involved in the interactions. This is particularly relevant given the effect that incubation at room temperature and polyAla expansions have on the spectrum of the fragment (see major points 1 and 2).

As explained in Extended Table 1 legend, we calculated the correlation times for the polyAlanine fragments included in each construction. Therefore, the correlation times correspond to a 20-Alanine stretch for XS20, 23 for XS23 and 26 for XS26. The flanking residues were excluded from the analysis due to their high intrinsic dynamics (heteronuclear NOEs values <0.5). Using our XS20 atomic structure, we calculated the theoretical correlation times for a monomeric conformation of the polyAlanine tract using HydroNMR. Our experimental values differ significantly from the theoretical ones (3.6 vs. 0.9 ns, respectively), which led us to conclude that XS20 is assembled into small oligomers in solution. Interestingly, the increase in correlation time for the 23 and 26 polyAlanine tracts cannot be explained by the simple expansion of the tract (+3 and +6 Alanines respectively, considering an increase in correlation time of 0.19 ns per every 3 Alanines in α-helical conformation, according to HydroNMR). If XS20 and XS26 were in the same assembled state in solution, assuming a correlation time of 3.6 ns for XS20, XS26 should show 3.98 ns, instead of the observed 4.7 ns. This discrepancy clearly indicates a higher tendency of XS26 for oligomer assembly.

Following the reviewer´s suggestion, we additionally obtained NMR parameters for XS20 at a significantly lower concentration (0.05 mM). As shown below (**Extended Figure 6**), the secondary chemical shits and relaxation parameters indicate that XS20 retains its structure, rigidity and assembly state at lower concentrations.

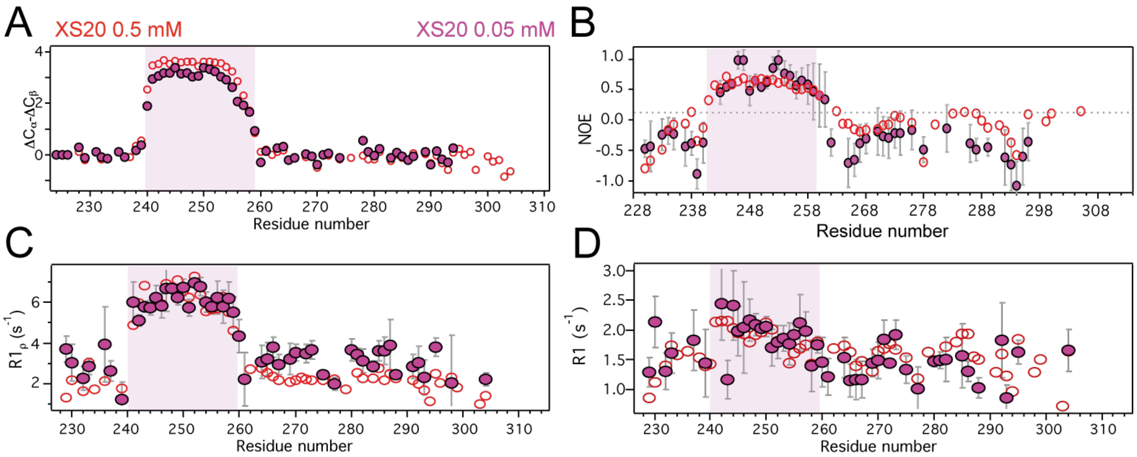

Extended Figure 6. Secondary chemical shifts and relaxation parameters for XS20 at lower concentration. A) Secondary chemical shifts plot for XS20 at 0.5 mM (red circles) and 0.05 mM (purple full circles). The color code is maintained in the figure. **B-D)** Heteronuclear NOE (**B**), R1ρ (**C**) and R1 (**D**) relaxation rates for XS20 in the mentioned conditions. Error bars are not included for XS20 at 0.5 mM for simplicity. Purple shade in the plots limit the α-helical region. All data included in this figure was obtained at 25 °C.

Therefore, XS20 at 0.5 mM is self-assembling into large oligomers which lay beyond NMR detection and the NMR signals observed (irrespective of the protein concentration and incubation) belong to species of similar assembly order. This figure and its discussion are included in the modified version of the manuscript.

4 - 193-194: what do the authors mean by “increase in order parameter” in this sentence ?

Decay in NMR signal intensity (Fig. 2D) can be due to self-association (increase in correlation time), presence of conformational exchange in the micro-to millisecond timescale and decrease of local flexibility (increase of order parameter S^2). Since the decrease in intensity is not clearly localized in a region of the protein but rather general, we conclude that the protein is undergoing self-association processes upon incubation generating species which are not detectable by NMR. Moreover, since the polyAlanine α-helix is not increasing its rigidity upon oligomerization (hetNOE values for XS20 vs XS26 are identical, Fig. 3B), the additional decay observed in the polyAlanine α-helical region (Fig. 2D) could be adscribed to slow conformational exchange between ordered and disordered conformations in the polyAlanine tract upon incubation.

5 - 219-22: the authors study equivalent fragments but with expanded polyAla tracts and observe, as expected, that the associated helices become longer and appear to have “a stronger propensity to establish length-dependent intermolecular association”. As in minor point 3 this needs to be studied, by NMR, by carrying out experiments at different concentrations.

We believe this issue has been discussed above in our response to Major point #1-3, Minor point #3.

6 - 230-231: the authors carry out heat denaturations of fragments with increasingly long polyAla tracts and claim that, whereas that of XS20 is reversible, those of XS23 and XS26 are not and instead lead to the formation of aggregates rich in beta structure. The CD spectrum of XS23 upon refolding differs from that expected for an aggregated rich in beta structure and resembles more that of an alpha helix: the authors should deconvolute the contribution of different secondary structures to the CD spectra.

We have deconvoluted the CD spectra using the BeStSel software (https://bestsel.elte.hu/index.php; Micsonai et al., Proc. Natl. Acad. Sci. USA (2015) doi:10.1073/pnas.1500851112.) and obtained the following values:

	XS20 fresh	XS20 refolding	XS23 fresh	XS23 refolding	XS26 fresh	XS26 refolding
α-helix	42,2	40,4	50,1	29,2	80,7	2,9
Antiparallel β	23,4	21	20,5	3,5	19,3	12
Parallel β	0	0	0	12,5	0	41,3
turn	0,1	1	0	3,2	0	6,9
other	34,4	37,5	29,3	51,5	0	37

The increase in β- and disordered structures in XS23 and XS26 upon thermal unfolding is evident from the deconvoluted data.

Reviewer #2 (Remarks to the Author):

The manuscript by Anton et al describes the structural features of a peptide fragment of the Phox2B protein (residues 228-314) that contains a poly-alanine sequence in a wildtype and disease-expanded form. The work shows by NMR that the poly-alanine is helical in both wild-type and disease-associated lengths. The work shows that the peptides self-associate into oligomers and condensates. All this behaviour of polyA is well-established knowledge by others cited by the authors. What appears to be novel however, is that there is an apparent switch from helix to disordered conformation in the long polyA lengths upon incubation, which coincides with a more mechanically rigid condensate state. It is proposed that this disordered condensate state may facilitate a further evolved conformational transition into amyloid structures from within that state. This point, if it is indeed real (as I have some questions on that below), would be an important advance to our understanding of how polyA sequences can lead to disease and help to reconcile some of the controversial and conflicting ideas about alpha-helix vs amyloid states of polyA condensates.

We appreciate the positive comments from the reviewer. In brief, in our manuscript we show fast liquid-to-solid transitions into small condensates that do not evolve into amyloids. In our experimental conditions, our data is in close agreement with work from Hatters´ (Polling, S. et al. Polyalanine expansions drive a shift into α-helical clusters without amyloid-fibril formation. *Nat Struct Mol Biol* **22**, 1008-15 (2015)) and Hnisz´s labs (Mensah, M.A. et al. Aberrant phase separation and nucleolar dysfunction in rare genetic diseases. *Nature* **614**, 564-571 (2023).), which showed no amyloid aggregates for polyAlanine expansions. As mentioned in the manuscript, endogenous PHOX2B is not able to form visible aggregates and only induced overexpression of the longer Alanine expansions promote PHOX2B protein deposition in cultured cells. In vitro, PHOX2B fragments were shown to form fibrillar aggregates after very long incubation times, but the integrity of the protein was not assessed after such periods in that study (Pirone, L., Caldinelli, L., Di Lascio, S., Di Girolamo, R., Di Gaetano, S., Fornasari, D., Pollegioni, L., Benfante, R. and Pedone, E. (2019), Molecular insights into the role of the polyalanine region in mediating PHOX2B aggregation. FEBS J, 286: 2505-2521. https://doi.org/10.1111/febs.14841). Therefore, we believe that our observations are in agreement with the proposed pathogenic mechanisms based on aberrant phase transitions for PARMs (Mensah, M.A. et al. Aberrant phase separation and nucleolar dysfunction in rare genetic diseases. *Nature* **614**, 564-571 (2023).

Key comments:

• How unequivocal is the point made from the NMR data there is a loss of helix and gain in disorder? Ie can the authors rule out the loss of helix arises from spectral broadening due to protein aggregation? Can the authors indicate how much of the helix is lost at the expense of disordered structure gained? Extra independent assessments on bulk average secondary structure should be considered, such as FTIR or CD under the same conditions as the NMR.

We have discussed above that loss of NMR signals (Fig. 2D) are due to self-association and conformational exchange. Therefore, PHOX2B constructs and undergoing time-dependent aggregation leading to species that lay beyond NMR detection. In addition, however, Fig. 2E shows a time-dependent decrease in α-helical content based on 3D NMR experiments (CBCA(CO)NH, in particular), which will show conformational-dependent averaged chemical shift values for each residue. Therefore, the decay in α-helical content observed in Fig. 2E is irrespective of spectral broadening but refers to conformational exchange towards disordered conformations. Due to the significant signal overlap for the residues from the core of the α-helix and, in general, for the nascent Alanine moieties in disordered conformations, calculating shifts in conformation populations based on NMR signal intensities may be prone to error. However, the reviewer makes a valid point suggesting doing CD under the same conditions as NMR. As shown below, we have acquired CD data using 0.5 mM XS26 (as in the NMR experiments) and performed thermal denaturation. The deconvolution of the data using BeStSel software produces the following values:

	XS26 fresh (0.5 mM)	XS26 refolding
α-helix	94,2	2,3
Antiparallel β	5,8	22,8
Parallel β	0	16
turn	0	18,1
other	0	40,8

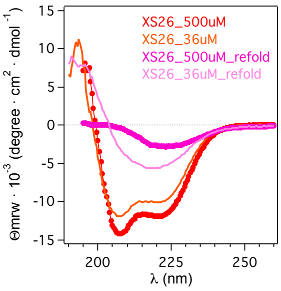

Therefore, our data suggest that PARMs promote disordered conformations which are coupled to aggregation (discussed in a previous paragraph).

• The authors conclude that the new resonances seen representing the disordered alanines are highly dynamic. Isn't this inconsistent with the other conclusion that the aged, expanded phox2B fragment forms solids?

Relaxation data shows that the incipient Alanine moieties are highly dynamic and origin from small oligomeric species (see previous responses). The condensates formed, although small in size, lay beyond NMR detection. Therefore, the novel conformations observed belong to species which originate prior to condensates, but are coupled to their formation. This increase in disorder is in agreement with recent studies showing extreme dynamics in the condensates (Farag M, et al. Condensates formed by prion-like low-complexity domains have small-world network structures and interfaces defined by expanded conformations. Nat Commun. 2022;13(1):7722. doi: 10.1038/s41467-022-35370-7. PMID: 36513655; PMCID: PMC9748015.; Galvanetto N, et al. Extreme dynamics in a biomolecular condensate. Nature. 2023;619(7971):876-883. doi: 10.1038/s41586-023-06329-5. PMID: 37468629) and rapid liquid-to-solid transitions promoted by PARMs in several proteins (Basu, S. et al. Unblending of Transcriptional Condensates in Human Repeat Expansion Disease. *Cell* **181**, 1062-1079.e30 (2020).).

• How specific are the effects of the chaperones to their chaperone activity? It is conceivable that non-specific binding interferes with the condensation, especially with the concentrations of chaperones used. Fig 6: Was there any ATP in the reaction? What happens if another "inert" protein was added, like BSA? What about the J-domain protein co-chaperone that is important for loading protein substrate to the Hsp70?

We thank the reviewer for raising this interesting point. We have performed additional titrations including the co-chaperone DNAJB1 in the presence of ATP and show, as could be expected, that the presence of the co-chaperone strongly suppressed the disordered conformations. Chaperone targeting to disordered conformations would induce signal broadening and, therefore, the decay in signal intensity is a direct measure of the decrease in population of polyAlanine disordered conformations in the presence of chaperones. In particular, HSP70 (in presence of ATP) decreased from 30-50% of the population of disordered conformations. Remarkably, the addition of the HSP70:DNAJB1 complex significantly impeded 80% of the disordered conformations in the polyAlanine tract, thus indicating selective targeting of disordered conformations as the basis for the chaperone-induced inhibition of PARM phase transitions. The data is included in the new Fig. 6D in the modified manuscript (included below for clarity).

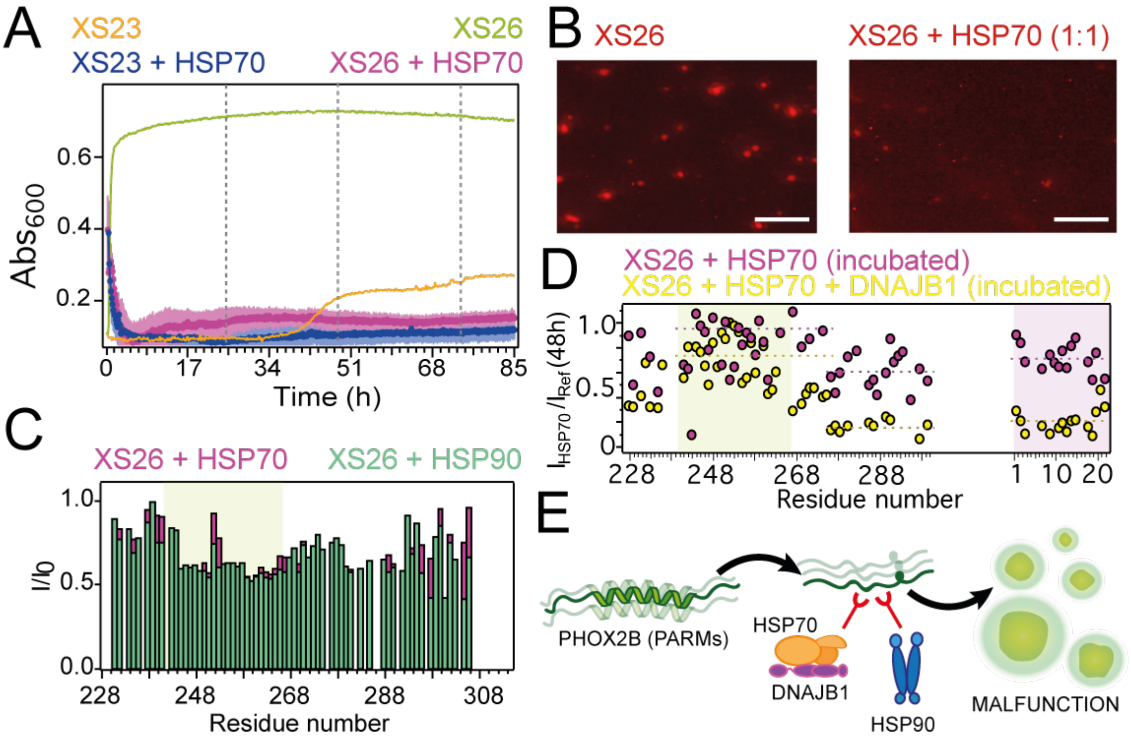

Figure 6. Chaperones suppress PHOX2B de-mixing arresting nascent conformations. A) Turbidity at 25°C for 0.225 mM XS23 or XS26 in presence of 0.1 mM HSP70 (blue and magenta, respectively). Plots for the unaccompanied PHOX2B constructs are included. **B)** Fluorescence microscopy images for XS26 (0.1 mM, labeled with ATTO-565) in the absence and presence of HSP70. Scale bars= 10 mm. **C)** ^{15}N-HSQC-based signal intensity plots for XS26 major conformation (0.07 mM) in complex with HSP70 (magenta) and HSP90 (green), all in 1:2 molar ratios. Green shadow limits polyAla α-helix. **D)** XS26 (0.225 mM) CACO signal intensity decay upon incubation (2 days at 25°C) in presence of HSP70 (1:2 equivalents, in magenta) and HSP70:DNAJB1 (1:2:1 equivalents, in yellow). Green shadow limits polyAla α-helix, while purple shadow limits nascent disordered alanine moieties. Broken lines mark the average intensity for the different segments. **E)** PARMs promote disorder in PHOX2B polyAla segment triggering fast liquid-to-solid phase transitions. Chaperones arrest nascent disordered conformations blocking phase separation.

Other comments:

• With respect to the sentence spanning lines 107-110: I don't think it is a requirement that proteins form a beta-sheet structure when forming a liquid-solid transition. Ref27 suggested that polyA8 forms amyloid - but the simulations were done at high temperature (330 K). At physiological temperatures polyA8 forms an alpha-helix as shown by others and in this manuscript.

We fully agree with the reviewer. Indeed, increasing evidence indicate that polyAla tracts do not transition to β-structure, and only β-type aggregation was obtained for short synthetic polyAlanine peptides and at extreme conditions. It is remarkably that very recent studies clearly showed that proteins in condensates remain highly disordered in condensate environments (Farag M, et al. Condensates formed by prion-like low-complexity domains have small-world network structures and interfaces defined by expanded conformations. Nat Commun. 2022;13(1):7722. doi: 10.1038/s41467-022-35370-7. PMID: 36513655; PMCID: PMC9748015; Galvanetto N, et al. Extreme dynamics in a biomolecular condensate. Nature. 2023;619(7971):876-883. doi: 10.1038/s41586-023-06329-5. PMID: 37468629).

Along these lines, our study represents, to our knowledge, the first experimental evidence of active transitions from a structured element towards disorder, since previous studies merely showed that disordered proteins retained disordered conformations in the condensates (Burke, K.A., Janke, A.M., Rhine, C.L. & Fawzi, N.L. Residue-by-Residue View of In Vitro FUS Granules that Bind the C-Terminal Domain of RNA Polymerase II. *Mol Cell* **60**, 231-41 (2015); Brady, J.P. et al. Structural and hydrodynamic properties

of an intrinsically disordered region of a germ cell-specific protein on phase separation. *Proc Natl Acad Sci U S A* **114**, E8194-E8203 (2017)).

• With respect to the sentence spanning lines 224-225: I suggest the authors tone this down as it was one study that had suggested that polyA can form coiled-coils under certain circumstances

Following the reviewer´s suggestions, we have rephrased the sentence.

• With respect to the sentence spanning lines 228-229: I suggest the authors frame the arguments more clearly in context of what ref 7 did. That study looked at different sequences to what was done here.

Following the reviewer´s suggestions, we have rephrased the sentence.

• Line 345: It is unclear what exact point the authors mean in this sentence for “are stably transmitted to offspring”. Does this refer to the DNA sequence? A protein prion-like aggregate? Either way the relevance is not clearly linked to the logic.

We agree with the reviewer that the statement “are stably transmitted to offspring” is not clearly linked to the arguments presented in that paragraph. We have removed the statement in the modified version of the manuscript.

• Line 383 statement “showed negative FRAP”. The logic is unclear since FRAP cannot be negative. Do the authors mean the proteins have no mobility as assessed by FRAP?

In agreement with the reviewer suggestion, we have rephrased the sentence now reading “showed absence of FRAP”. The reviewer´s interpretation is correct: proteins show negligible mobility as assessed by FRAP.

• Line 419 – do the authors mean “steady-state” instead of “steady”? Please clarify.

We are grateful to the reviewer for her/his comment and have corrected the statement to “steady state” in the modified version of the manuscript.

• With reference to the sentence between lines 413-416: The logic of the sentence appears incorrect since polyQ does not become disordered from the helical state - it becomes fibrillar and beta sheet from either a disordered or potentially helical state.

In agreement with the reviewer´s suggestion, we have removed the statement in the modified version of the manuscript.

• With reference to the sentence between lines 416-418: Best to rephrase this sentence as the sentence implies prion-like domains refers to polyA.

In agreement with the reviewer´s suggestion, we have modified the statement, now reading “protein chains”.

• The section between lines 428 and 434 do not make sense to me. Wouldn't the logic be more sensical if polyA were stickers? Afterall this work and that of others have shown polyA self assembles into oligomers and larger condensates.

This point raised by the reviewer is indeed very interesting. Intriguingly, Mier and coworkers recently observed that Gly and Pro are systematically found in the proximities of polyAla tracts (Pablo Mier, Carlos A Elena-Real, Juan Cortés, Pau Bernadó, Miguel A Andrade-Navarro, The sequence context in poly-alanine regions: structure, function and conservation, *Bioinformatics*, Volume 38, Issue 21, 1 November 2022, Pages 4851–4858, https://doi.org/10.1093/bioinformatics/btac610). If one runs XS26 sequence in a predictor of phase separation propensities such as FuzDrop (Vendruscolo M, Fuxreiter M. Sequence Determinants of the Aggregation of Proteins Within Condensates Generated by Liquid-liquid Phase Separation**.** *J Mol Biol. (2021);167201-167212* 10.1016/j.jmb.2021.167201), the result clearly indicates that the polyAla tract is not driving phase separation, but rather the flanking sequences, rich in Gly and Pro residues.

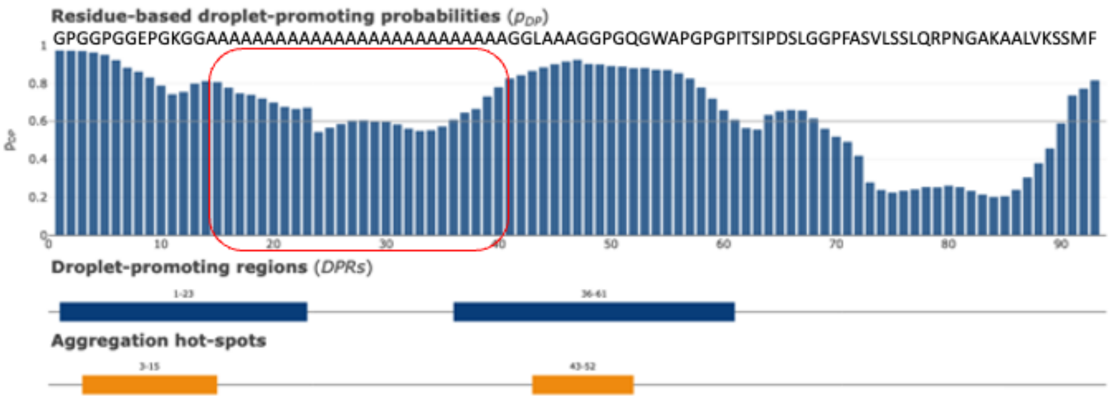

FuzDrop prediction of XS26 phase separation propensities. Values higher than 0.6 in the P_{CP} plot indicate strong propensity to phase separate. XS26 sequence is indicated on top. The red box limits the polyAla fragment. The sequences flanking the polyAla tract, in particular containing the sequences GPGGPGGEPGKGG and GGLAAAGGPGQGWAPGPG are predicted to drive phase separation.

• With reference to the sentence between lines 432-435: The logic is unclear and unexplained as to why the prior observations on polyQ-driven assemblies disagree with the point made about polyA aggregation.

In their study, Zhang and coworkers (Zhang, H. et al. RNA Controls PolyQ Protein Phase Transitions. *Mol Cell* **60**, 220-30 (2015)) showed that the disordered long polyQ tract found in the protein Whi3 from *Ashbya gossypii* is key facilitating multiple contacts between the RRM domain and mRNA chains, which promote phase separation *in vivo*. Thus, increased flexibility in this particular segment enables sticking contacts during phase transition, in agreement with the proposed destabilizing effect for PARMs in PHOX2B. To avoid misleading, we have rephrased the statement in the manuscript, now reading "Therefore, it follows that conformational changes and increased disorder in the hydrophobic polyAla spacer would facilitate contacts within the stickers in the dense phases[28]. This is somehow in disagreement with the premise stating that polyAla expansions trigger protein aggregation through coiled coil stabilization[7], but is in accordance with the role of disordered polyQ segments supporting Whi3 phase separation[56]".

• Line 454: It is unclear why the authors are indicating GPG are stickers – the reference they cite to this effect does not as far as I can read indicate these residues to act as stickers. I would expect that they would be spacers, especially in this context.

We are grateful to the reviewer for raising this issue. To avoid misleading, and in relation to the previous explanation of the sticker patterning in PHOX2B, we have rephrased the statement, now reading: "Considering that the polyAla-flanking stickers in PHOX2B display significant polymorphism due to proline *cis-trans* isomerization, enriched disorder in the polyAla tract would increase networking during phase transitions".

• Figure 2C: Is the protein forming a gel halfway up the tube in Fig 2C? It is a little hard to see the details of what is happening in the image. I suggest the authors consider a different way to show the result.

The picture in Fig. 2C shows that the NMR sample is becoming turbid upon incubation. A similar effect is shown in the new **Extended Figure 19D.**

• In Figure 3, I suggest that the temperature used to thermally denature the proteins be stated in the legend. The figure title suggests "irreversible" unfolding. But the methods indicate the results are formed after only a few minutes cool down. Would the alpha-helix return eventually (ie if it re-established equilibrium?).

Following the reviewer´s suggestion, we have modified Fig. 3 legend. In addition, we have repeated the thermal denaturation experiments now extending the equilibration time for 2h, and the results replicated the irreversible unfolding described in the manuscript.

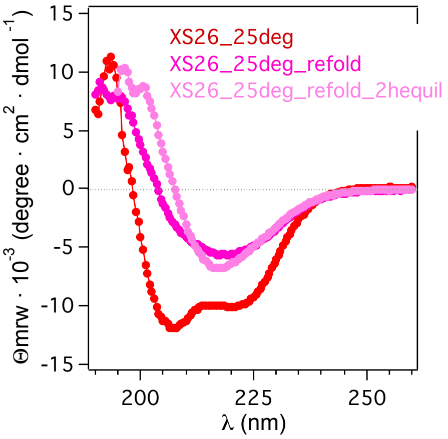

Extended equilibration does not re-establish refolding for PARMs. XS26 (in red) was heat denatured at 95 °C for 15 minutes and cooled down to 25 °C for 6 minutes (magenta). Spectra were additionally recorded after 2h equilibration at 25 °C (light pink), showing absence of α-helical refolding.

• Figure 4B: In the graph, is the data showing the chemical shift for the incubated sample to be zero in the helical section? Or are there bars buried behind the green bars?

The plot in Fig. 4B shows the secondary chemical shits obtained from 3D CBCA(CO)NH spectra. The polyAla α-helical regions, including residues Ala241-Ala266 are broadened beyond detection in the 3D spectra, and no secondary chemical shifts could be obtained. Only the alternative Ala moieties show signals in these spectra, which contain disordered conformations according to their secondary chemical shift values.

• For Fig 4D, I suggest the authors split the graph between the left and right parts because they are not plotting the same features on the x-axis. At first glance it appears the residue numbering on the right section applies to the main protein chain sequence rather than the arbitrary alanines denoted in panel A.

Following the reviewer´s suggestion, we have modified Fig. 4 and Extended Figure 10 in the manuscript.

• I don't understand what is being plotted for the two colour samples in Extended Fig 11. Why are there not two colours taken for the same field of view?

Final panels in Extended Figure 11 show incubations with both proteins attached to different dyes, and in both combinations (XS26 green + XS20 red and XS26 red + XS20 green). The images show that XS20 is incorporated at a slower rate into the condensates. The images were not obtained on the same field of view due to technical limitations.

• Statiscal issues: need to define the error bars in Fig 1E. How many samples were used to calculate the SD in Extended Table 1?

The error bars included in Fig. 1E (relaxation data) were defined in the Materials & Methods section: "Relaxation values and uncertainties were calculated by fitting an exponential decay to the data. Het-NOEs were calculated from the ratio of cross peak intensities in spectra collected with and without amide proton saturation during the recycle delay. Uncertainties in peak heights were determined from the standard deviation of the intensity distribution in signal-less spectral regions". In Extended Table 1, data for each construct and condition include average +/- SD. The SD refers to the statistical variability between the values obtained for the protein sequences considered (either the full construct sequence or only the polyAla tract).

REVIEWER COMMENTS

Reviewer #1 (Remarks to the Author):

The authors have conducted experiments to address the oligomerisation state of the species detected by NMR.

It now seems clear that the NMR signals correspond to an oligomer, that its oligomerisation state does not change substantially during the study, that the majority of the protein molecules are not observed in the experiment and, finally, that the chemical shift changes observed are due to a structural change in the oligomer.

This result can explain why such changes occur at a very slow timescale: one would expect conformational changes in a monomeric protein, especially rich in intrinsic disorder, to be fast but substantially slower in an oligomer.

A first remaining problem of this manuscript is that it claims to have characterised the conformational changes responsible for a liquid-to-solid phase transition in a condensate, whereas this is not the case: the authors have observed by NMR a conformational change promoted by polyA expansion in an oligomeric state that they have detected but not characterised in any way.

A second problem of this manuscript is the liquid-to-solid transition that the authors is claimed is associated with the polyA expansion is not characterized: the main difference between the proteins with normal and expanded tracts is that the latter phase separate whereas the former does not and none of the condensates to have the properties of liquids at any stage.

In summary, the manuscript presents interesting NMR evidence of a conformational change occurring in oligomers, favoured by polyAla expansion, evidence that expansion also increases aggregation rate and a speculation on the possibility that the solid character of the aggregates is due to a conformational change equivalent to that observed in the oligomers.

The mechanistic model that the authors have presented in Extended Data Figure 20 is possible, even likely, but the substantial experimental challenges that the authors have encountered in this work have as a consequence that the claims, in the current manuscript, are not sufficiently supported by the data.

If the authors were to soften their claims I would be happy to support publication of this work.

Reviewer 1 report
Comments on how the authors have addressed my concerns
*****

The authors have conducted experiments to address the oligomerisation state of the species detected by NMR.

It now seems clear that the NMR signals correspond to an oligomer, that its oligomerisation state does not change substantially during the study, that the majority of the protein molecules are not observed in the experiment and, finally, that the chemical shift changes observed are due to a structural change in the oligomer.

We agree with the reviewer´s interpretation. All the data indicated that the proteins are initially oligomeric and that the conformational changes towards disordered species, which are coupled to solid condensate formation, occur in similar oligomeric species. It is unsurprising that signals from the proteins within the solid condensates are not visible in solution NMR. Therefore, the new set of signals observed in the NMR spectra do not belong to solid condensates but rather to precursor (small oligomeric) species.

This result can explain why such changes occur at a very slow timescale: one would expect conformational changes in a monomeric protein, especially rich in intrinsic disorder, to be fast but substantially slower in an oligomer.

We fully agree with the reviewer. Unfortunately, considering the large content in disorder in the proteins, determining the oligomeric state of the visible species from the experimental data (either from light scattering or NMR data, as performed here) would be prone to error, especially considering that there is a significant conformational change (from α-helix to disorder) involved in the process. Still, this uncertainty in the initial oligomeric state of the proteins (which is discussed in detail below) does not affect the main conclusions of the work, namely:

- The atomic structures of the C-terminal domain of human PHOX2B shown in our manuscript represent, to our knowledge, the first atomic structures described for protein homorepeats. Contrary to what is established, the rigid polyAlanine α-helices are not significantly assembled into coiled coil arrangements, at least at higher temperatures.
- Using an integrative approach that comprises state-of-the-art solution NMR spectroscopy and superresolution optical microscopy, we show that disease-causing PARMs do not alter PHOX2B main structural conformation. Alternatively, pathogenic PARMs promote nascent metamorphs that prime fast phase transitions into significantly small, solid biomolecular condensates that capture wild-type PHOX2B.
- We demonstrate that disordered metamorphs induced by PARMs are the template structures that prompt phase transitions, in agreement with recent theoretical studies. To our knowledge, our study represents the first experimental observation of structural conversions coupled to phase transitions in high resolution.
- We show that molecular chaperones, known to be directly involved in the clearance of pathogenic PHOX2B, selectively arrest nascent conformations promoted by PARMS suppressing phase transitions. This selective recognition provides a direct link between structural polymorphism and phase transitions and presents small solid condensates as novel pathogenic species in human diseases caused by expanded homorepeat mutations.

A first remaining problem of this manuscript is that it claims to have characterised the conformational changes responsible for a liquid-to-solid phase transition in a condensate, whereas this is not the case: the authors have observed by NMR a slow conformational change,

promoted by polyA expansion, in an oligomer that they have detected by NMR for which they do not know the oligomerization state.

We agree with the reviewer and apologize for the misleading: We do not claim to characterize "liquid-to-solid phase transition in a condensate", as the reviewer states. Firstly, because the condensates observed formed rapidly and showed only solid character. Therefore, we rather characterize fast phase transitions towards formation of small, solid condensates promoted by the PARMs, as shown in **Figure 5** and in **Extended Figures 13-17**. This is in agreement with the reviewer when she/he claims "none of the condensates to have the properties of liquids at any stage", as shown in the next paragraph. We agree that corrections needed to be included in the manuscript to avoid misleading and the attached version highlights that all the "liquid-to-solid transition" statements in the previous versions were modified to "phase transitions to small solid condensates".

We also agree with the reviewer in the uncertainties on the oligomerization state of the species visible by NMR. Considering the high dynamic nature and small size of the proteins, the best ways to address the oligomerization state of the proteins are NMR-based. We first attempted to determine the multimeric state by relaxation experiments (**Extended Table 1**). Here, the correlation times could only be obtained for the structured regions (meaning the polyAla α-helical tracts) using the Lipari-Szabo ModelFree formulism (Lipari, G.; Szabo, A. (1982) J. Am. Chem. Soc. 104, 4546) and Nicholson's recommendations (Pawley, N. H., Wang, C., Koide, S., and Nicholson, L. K. (2001) J. Biomol. NMR 20, 149– 165). Applying these methods for the rest of the disordered segments included in the proteins would induce significant artifacts since the Stoke's law cannot be applied and the effective hydrodynamic radius cannot be estimated. As shown in **Extended Table 1**, the correlation times for the ordered α-helical tract do not display a concentration dependence and are rather large (3,6 ns) for a 20 residue α-helix. Following the equation:

$$Mw = 1.493\ Tc + 1.1187$$

from (Rossi P. et al. (2010) J. Biomol. NMR 46(1):11-22), obtained based on measurements at 25 ºC, the Mw corresponding to a correlation time of 3,6 ns would be 6,49 kDa. Considering a nominal molecular weight for a monomeric 20-alanine peptide of 1,4 kDa, one could argue that the protein seems to engage in tetrameric species. Following the same logic, the 4,0 ns and 4,7 ns of correlation times obtained for the 23- and 26-alanine segments (**Extended Table 1**) would correspond to 7,09 and 8,13 kDa, respectively. If we consider a nominal molecular weight of 1,65 and 1,86 kDa for the respective monomeric fragments, we could conclude that the proteins are tetrameric in origin.

Moreover, the theoretical correlation time obtained by HydroNMR on a 20-alanine α-helix is 0,9 ns, compared to the 3,6 ns experimental value. This difference could be explained if we assume the tetrameric organization of the polyAla α-helical tract. The theoretical correlation times are not calculated on a 23 residue α-helix nor a 26-residue α-helix due to the lack of experimental structures for the fragments. However, if we assume an increase of 0,19 ns for a canonical α-helix of 3 alanine residues, it would lead to a theoretical value of 1,09 ns for a monomeric 23 alanine α-helix (0,9+0,19 ns), and 1,28 ns for a monomeric 26 alanine α-helix. Significantly, the experimental correlation times were 4,0 and 4,7 ns for 23- and 26-alanine α-helices, respectively. Again, the disparity between the expected correlation times and the experimentally obtained values could correspond to tetrameric arrangements of all the studied proteins in solution. This is in close agreement to the reviewer´s statement:"… that its oligomerization state does not change substantially during the study".

Nonetheless, we observed that there is an increase in disordered conformations in the alanine tract with time (**Extended Figure 11**), which could impact the spectral density terms and, thus, the calculation of the correlation times (Kay, L. E. et al. (1989) Biochemistry 28 (23): 8972-9).Therefore, and in agreement with the reviewer´s suggestion, we decided to address the oligomerization state also by diffusion NMR. Contrary to other methods which can inherently lead to high errors in the calculation of radius of gyration or hydrodynamic parameters such as SAXS or light scattering methods coupled to size-exclusion chromatography, the hydrodynamic radius derived from the diffusion coefficient could unambiguously characterize the protein conformational state (Dudás & Bodor, (2019) Anal. Chem. 91: 4929-33). However, measurements of diffusion coefficients related to peptides with different masses would have systematic errors if the structure of the peptides changed

significantly for different molecular weights (Danielsson et al. (2002) Mag. Resn. Chem. 40: S89-97). Because the proteins have a mixture of folded and disordered fragments, with increasing amounts of disorder in the case of the PARMs (**Extended Figures 11, 12**), one should be cautious when calculating effective hydrodynamic radii from the diffusion coefficients (Dudás & Bodor, (2019) Anal. Chem. 91: 4929-33). The diffusion coefficients obtained are: 1,409 E-10 and 1,451 E-10 m^2s^{-1} for 20-alanine and 26-alanine constructs, respectively (**Extended Figure 3C-D**). Following the approximations presented in (Dudás & Bodor, (2019) Anal. Chem. 91: 4929-33), these values could roughly represent hydrodynamic radius of 13 Å for a pure disordered protein and 14 Å for a folded protein. For the pure disordered protein, these diffusion coefficients could correspond to approximately 2 kDa peptides, while they would correspond to a folded protein of 3,16-3,5 kDa. In addition, these diffusion constants are in line with those presented for monomeric Aβ peptides at similar pH values (Danielsson et al. (2002) Mag. Resn. Chem. 40: S89-97).

One could argue that relaxation experiments, being more time consuming than diffusion, could reflect more closely the precise oligomerization state where the conformational changes are observed (**Extended Figure 9**). Nonetheless, the inconsistencies in the calculated molecular masses from the NMR observables (relaxation vs. diffusion) highlight the inherent inaccuracy when extracting the oligomeric state of a small protein with large disordered regions and a tendency to undergo conformational changes in solution. Therefore, we believe that providing a quantitative analysis of the oligomerization state is too speculatory and prone to miscalculation, and only qualitative information should be obtained from the observables: conformational changes promoted by PARMs occur in similar order species which are precursors to the fast phase transition into solid condensates (the latter being invisible in NMR). In the manuscript, we present the experimental data rather than the estimations.

In addition, we provide significant evidences showing that the "conformational change promoted by polyA expansion in an oligomer" is specifically towards the dismantling of the α-helix into disordered conformations in the polyAla tract (**Figure 4** and **Extended Figures 9-12**). Moreover, we provide compelling evidence showing that these novel disordered conformations are coupled to the fast phase transitions into small, solid condensates (**Figure 6** and **Extended Figures 18-19**).

A second remaining problem of this manuscript is that the liquid-to-solid transition that the authors is claimed is associated with the polyA expansion is not characterized: the main difference between the proteins with normal and expanded tracts is that the latter phase separate whereas the former do not and none of the condensates to have the properties of liquids at any stage.

We fully agree with the reviewer and, again, apologize for the misleading. The reviewer´s interpretation is correct: wild type protein does not phase separate, while the expanded mutant shows a rapid phase separation into solid condensates. This is shown in **Figure 5** and **Extended Figure 13**, and is already presented in the abstract: "*However, polyalanine expansions in PHOX2B additionally promote nascent homorepeat conformations that trigger length-dependent phase transitions into solid condensates that capture wild-type PHOX2B".* This is different than stating that the condensates formed by the wild type remained liquid whereas those from the expanded mutant transitioned into solid condensates, as could be inferred from the reviewer´s comment. If the reviewer´s statement were to be true, we would agree that additional evidence were required. However, the main statement regarding this aspect should read "*Pathogenic PARMs trigger rapid phase transitions"* as it appears in the modified version of the manuscript. Since this misunderstanding could arise from the "liquid-to-solid transition" term present several times in the previous version of the manuscript, we removed it from the modified version.

In summary, the manuscript presents interesting NMR evidence of a conformational change occurring in oligomers, favoured by polyAla expansion, evidence that expansion leads to condensation/aggregation and a speculation on the possibility that the solid character of the aggregates is due to a conformational change equivalent to that observed by NMR in the oligomers.

We thank the reviewer for the positive comments. We agree with the conclusions of the referee except for the one stating that "the solid character of the aggregates is due to a conformational change". We do not claim that in the manuscript. According to our data, the conformational change is responsible for the phase transition into solid condensates, but if the protein did not change in conformation (like the wild type) it would not phase separate. This is different than saying that the condensates formed by the protein not showing the conformational change would remain liquid (as opposite to solid), which would go in line with the reviewer´s statement. This is a misunderstanding of the claims presented in the manuscript and appears several times in the reviewer´s comments. Therefore, we have modified the manuscript to avoid misleading.

In brief, proteins are initially oligomeric, PARMs promote incipient disordered species which prime a fast and irreversible phase transition into small solid condensates. The wild type protein does not promote these novel conformations and does not phase separate. We trust these statements are now clear in the modified manuscript.

The mechanistic model that the authors have presented in Extended Data Figure 20 is possible, even likely, but the substantial experimental challenges that the authors have encountered in this work have as a consequence that the claims, in the current manuscript, are not sufficiently supported by the data.

We disagree with the reviewer and believe that the claims in the manuscript (summarized above) are strongly supported by the data. Extended Data Figure 20 shows an interpretation of our novel data in the light of the available bibliography: PHOX2B is shown to reversibly phase separate, at least in the nucleus (Basu S, et al. Cell. 2020 181(5):1062-1079. doi: 10.1016/j.cell.2020.04.018), although we do not see phase separation from the isolated construct. In turn, expanded mutants rapidly phase separate into solid, irreversible and small condensates. Although we believe the figure could help interpret our data in light of the available evidences, if the reviewer believes that the Extended Data Figure 20 is misleading we would happily remove it from the manuscript.

If the authors were to at least estimate the oligomerization state of the oligomers as well as soften their claims and better differentiate between their conclusions and their speculations I would be happy to support publication of this work.

We appreciate the comments by the reviewer and the positive feedback. We believe we have adequately discussed the oligomerization state of the proteins and clarified some issues raised due to misleading. We also believe that all our conclusions are supported by the data and we have corrected this misleading and toned down the claims in the modified version of the manuscript.

We therefore trust that the points raised by the reviewer are now straightforward.

Comments on how the authors have addressed the concerns of reviewer 2
****

The concerns of this reviewer about the interpretation of the results of the NMR experiments have been mainly addressed in the revised version.

We thank the reviewer for the positive comments.

The authors also addressed well the request to investigate the specificity of the interaction between the disordered conformation of the oligomer and molecular chaperones.

We thank the reviewer for the positive comments. We believe this is a key point of the manuscript.

The other requests of the reviewer, mainly minor changes and requests for clarification, have also been addressed.

We thank the reviewer for the positive comments.

REVIEWERS' COMMENTS

Reviewer #1 (Remarks to the Author):

The authors have made changes to the text. It now better reflects their results. They provide a very detailed explanation of why it is difficult to know the oligomerization state from NMR. This is very interesting and true but does not take into account the existence of other techniques that can be used to determine the oligomerization state such as analytical ultracentrifugation. Despite this I support publication of this paper in Nature Communications.
